# Inherited human ITK deficiency impairs IFN-γ immunity and underlies tuberculosis

**DOI:** 10.1084/jem.20220484

**Published:** 2022-11-03

**Authors:** Masato Ogishi, Rui Yang, Rémy Rodriguez, Dominic P. Golec, Emmanuel Martin, Quentin Philippot, Jonathan Bohlen, Simon J. Pelham, Andrés Augusto Arias, Taushif Khan, Manar Ata, Fatima Al Ali, Flore Rozenberg, Xiao-Fei Kong, Maya Chrabieh, Candice Laine, Wei-Te Lei, Ji Eun Han, Yoann Seeleuthner, Zenia Kaul, Emmanuelle Jouanguy, Vivien Béziat, Leila Youssefian, Hassan Vahidnezhad, V. Koneti Rao, Bénédicte Neven, Claire Fieschi, Davood Mansouri, Mohammad Shahrooei, Sevgi Pekcan, Gulsum Alkan, Melike Emiroğlu, Hüseyin Tokgöz, Jouni Uitto, Fabian Hauck, Jacinta Bustamante, Laurent Abel, Sevgi Keles, Nima Parvaneh, Nico Marr, Pamela L. Schwartzberg, Sylvain Latour, Jean-Laurent Casanova, Stéphanie Boisson-Dupuis

**Affiliations:** 1 St. Giles Laboratory of Human Genetics of Infectious Diseases, Rockefeller Branch, Rockefeller University, New York, NY; 2 The David Rockefeller Graduate Program, Rockefeller University, New York, NY; 3 Laboratory of Lymphocyte Activation and Susceptibility to EBV Infection, INSERM UMR1163, Paris, France; 4 Imagine Institute, University of Paris Cité, Paris, France; 5 Cell Signaling and Immunity Section, Laboratory of Immune System Biology, National Institute of Allergy and Infectious Diseases, National Institutes of Health, Bethesda, MD; 6 Laboratory of Human Genetics of Infectious Diseases, Necker Branch, INSERM U1163, Paris, France; 7 Primary Immunodeficiencies Group, University of Antioquia UdeA, Medellin, Colombia; 8 School of Microbiology, University of Antioquia UdeA, Medellin, Colombia; 9 Department of Immunology, Research Branch, Sidra Medicine, Doha, Qatar; 10 Department of Virology, Cochin Hospital, University of Paris, Paris, France; 11 Department of Dermatology and Cutaneous Biology, Sidney Kimmel Medical College, Philadelphia, PA; 12 Jefferson Institute of Molecular Medicine, Thomas Jefferson University, Philadelphia, PA; 13 Laboratory of Clinical Immunology and Microbiology, Division of Intramural Research, National Institute of Allergy and Infectious Diseases, National Institutes of Health, Bethesda, MD; 14 Pediatric Immunology and Hematology Department, Necker Hospital for Sick Children Assistance Publique-Hôpitaux de Paris (AP-HP), Paris, France; 15 Clinical Immunology Department, Saint Louis Hospital, AP-HP Université de Paris, Paris, France; 16 INSERM UMR1126, Institut de Recherche Saint-Louis, Université de Paris, Paris, France; 17 Clinical Tuberculosis and Epidemiology Research Center, National Research Institute of Tuberculosis and Lung Diseases, Shahid Beheshti University of Medical Sciences, Tehran, Iran; 18 Department of Microbiology and Immunology, Laboratory of Clinical Bacteriology and Mycology, KU Leuven, Leuven, Belgium; 19 Department of Pediatric Pulmonology, Necmettin Erbakan University, Meram Medical Faculty, Konya, Turkey; 20 Division of Pediatric Infectious Diseases, Department of Pediatrics, Selcuk University Faculty of Medicine, Konya, Turkey; 21 Department of Pediatric Hematology, Meram School of Medicine, Necmettin Erbakan University, Konya, Turkey; 22 Division of Pediatric Immunology and Rheumatology, Department of Pediatrics, Dr. von Hauner Children’s Hospital, University Hospital, Ludwig-Maximilians-Universität München, Munich, Germany; 23 Center for the Study of Primary Immunodeficiencies, Necker Hospital for Sick Children Assistance Publique-Hôpitaux de Paris (AP-HP), Paris, France; 24 Division of Pediatric Allergy and Immunology, Necmettin Erbakan University, Meram Medical Faculty, Konya, Turkey; 25 Division of Allergy and Clinical Immunology, Department of Pediatrics, Tehran University of Medical Sciences, Tehran, Iran; 26 Research Center for Immunodeficiencies, Tehran University of Medical Sciences, Tehran, Iran; 27 College of Health and Life Sciences, Hamad Bin Khalifa University, Doha, Qatar; 28 Department of Pediatrics, Necker Hospital for Sick Children, Paris, France; 29 Howard Hughes Medical Institute, New York, NY

## Abstract

Inborn errors of IFN-γ immunity can underlie tuberculosis (TB). We report three patients from two kindreds without EBV viremia or disease but with severe TB and inherited complete ITK deficiency, a condition associated with severe EBV disease that renders immunological studies challenging. They have CD4^+^ αβ T lymphocytopenia with a concomitant expansion of CD4^−^CD8^−^ double-negative (DN) αβ and Vδ2^−^ γδ T lymphocytes, both displaying a unique CD38^+^CD45RA^+^T-bet^+^EOMES^−^ phenotype. Itk-deficient mice recapitulated an expansion of the γδ T and DN αβ T lymphocyte populations in the thymus and spleen, respectively. Moreover, the patients’ T lymphocytes secrete small amounts of IFN-γ in response to TCR crosslinking, mitogens, or forced synapse formation with autologous B lymphocytes. Finally, the patients’ total lymphocytes secrete small amounts of IFN-γ, and CD4^+^, CD8^+^, DN αβ T, Vδ2^+^ γδ T, and MAIT cells display impaired IFN-γ production in response to BCG. Inherited ITK deficiency undermines the development and function of various IFN-γ–producing T cell subsets, thereby underlying TB.

## Teaser

Inherited ITK deficiency shows incomplete penetrance for severe EBV disease and can underlie severe tuberculosis due to insufficient IFN-γ production by innate-like adaptive (Vδ2^+^ γδ T and MAIT) and purely adaptive αβ T lymphocytes.

## Graphical Abstract

**Figure:**
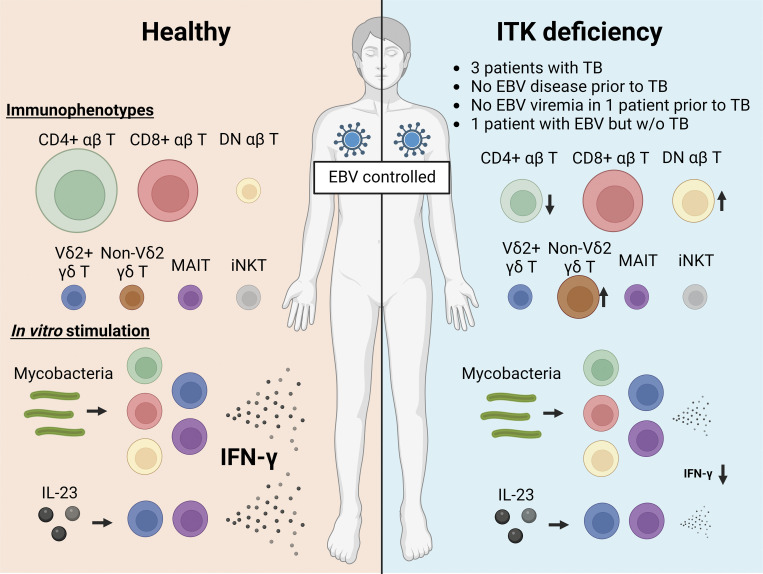

## Introduction

Tuberculosis (TB) is caused by virulent mycobacterial species from the *Mycobacterium tuberculosis* complex (MTBC), including *Mycobacterium tuberculosis* (*M. tb*) and *Mycobacterium bovis*. TB remains endemic in many countries (Houben and Dodd, 2016). The World Health Organization has estimated that there were ∼9 million new cases and ∼1.2 million deaths globally among HIV-negative individuals in 2019 (WHO, 2020). However, only ∼5–10% of individuals infected with *M. tb* develop TB in their lifetime (Vynnycky and Fine, 2000). Classical genetic studies performed from 1909 onward suggested that human genetic determinants strongly influence whether individuals develop TB or remain asymptomatic following infection (Kallmann and Reisner, 1943; Comstock, 1978; Bellamy, 1998; Van Der Eijk et al., 2007; Abel et al., 2014, 2018; Boisson-Dupuis et al., 2015; Casanova and Abel, 2022). However, the molecular basis of susceptibility to TB has long remained unknown (McHenry et al., 2020). The study of Mendelian susceptibility to mycobacterial disease (MSMD), a rare condition that selectively predisposes ∼1/50,000 individuals to severe disease caused by weakly virulent mycobacteria (i.e., Bacille Calmette-Guérin [BCG] vaccine substrain and environmental mycobacteria), has identified IFN-γ as a key component of human antimycobacterial immunity (Bustamante, 2020). Indeed, 18 of the 19 known monogenic etiologies of MSMD impair IFN-γ production (variants of *IFNG*, *IL12B*, *IL12RB1*, *IL12RB2*, *IL23R*, *TYK2*, *ISG15*, *RORC*, and *TBX21*), cellular responses to IFN-γ (*IFNGR1*, *IFNGR2*, *STAT1*, *JAK1*, *USP18*, and *CYBB*), or both (*IKBKG*, *IRF8*, and *SPPL2A*; Bustamante, 2020; Kerner et al., 2020; Yang et al., 2020; Martin-Fernandez et al., 2022). The mechanism of disease in patients with *ZNFX1* mutations has yet to be determined (Le Voyer et al., 2021)*.* Patients with these disorders are also prone to TB, which may even be the only clinical phenotype for genotypes displaying incomplete penetrance for MSMD. Multiple patients with autosomal recessive (AR) complete TYK2 and IL-12Rβ1 deficiencies have been diagnosed with TB (Altare et al., 2001; Kreins et al., 2015; Boisson-Dupuis and Bustamante, 2021; Ogishi et al., 2022). Moreover, homozygosity for *TYK2* P1104A, which selectively impairs IL-23–dependent IFN-γ production, is a common and highly penetrant genetic etiology of TB, but a rare and weakly penetrant etiology of MSMD (Boisson-Dupuis et al., 2018; Kerner et al., 2019, 2021; Casanova and Abel, 2022). Finally, we recently described a Turkish child with AR PD-1 deficiency and life-threatening TB who had not suffered from MSMD (Ogishi et al., 2021). His TB probably resulted from impaired, but not abolished, IFN-γ production due to low levels of innate-like adaptive T lymphocytes and dysfunction of the residual T and natural killer (NK) lymphocytes. Other inborn errors of immunity (IEI), such as *STAT3* gain-of-function (GOF) and *PIK3CD* GOF, appear to predispose humans to TB by hitherto unexplained mechanisms (Haapaniemi et al., 2015; Boisson-Dupuis and Bustamante, 2021; Qiu et al., 2022). In this context, we studied three patients from two unrelated kindreds with unexplained severe TB and no prior history of MSMD.

## Results

### Three patients with disseminated TB

**Figure 1.**
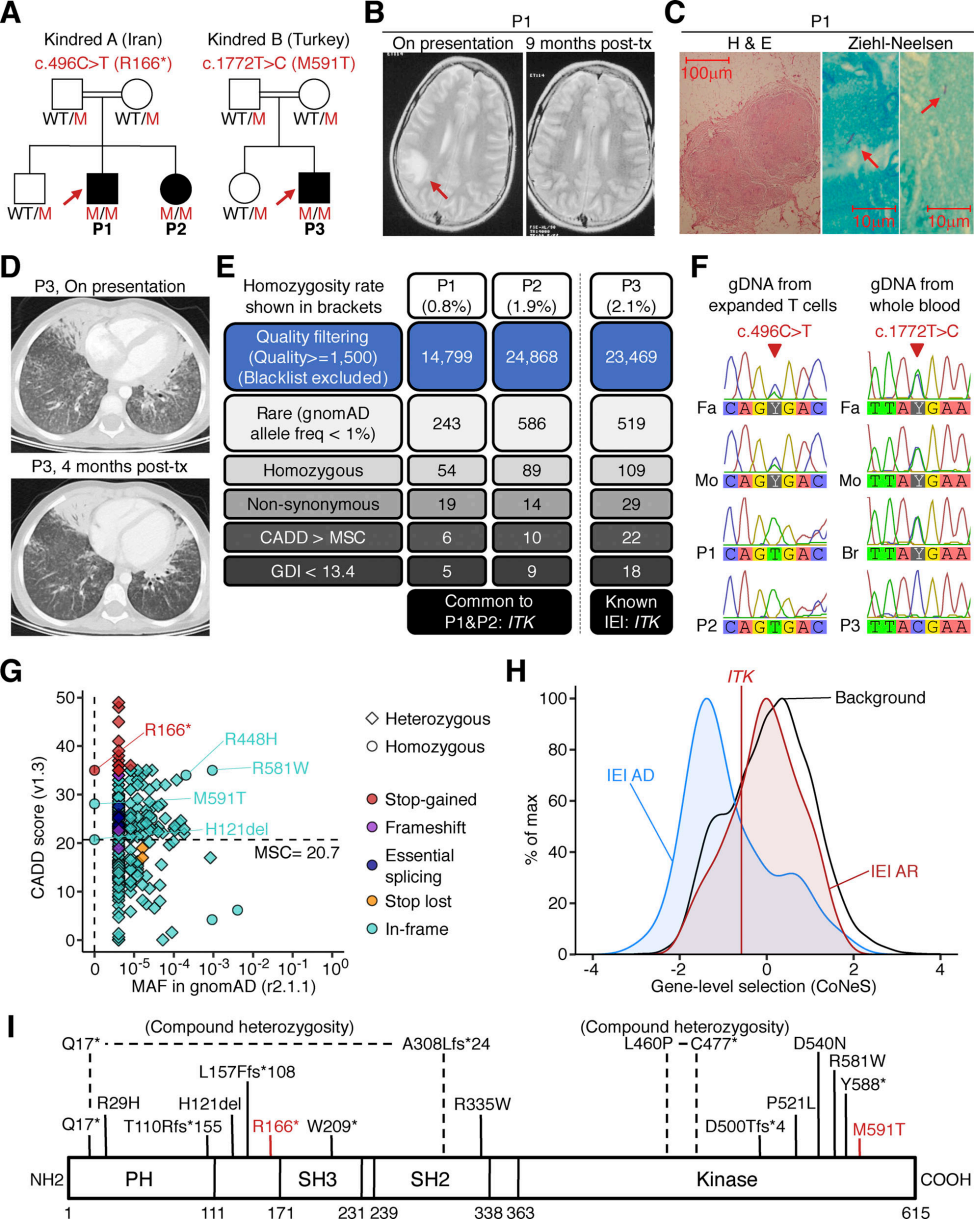
**AR ITK deficiency with TB. (A)** Pedigree of the kindreds. Black symbols indicate affected individuals. Arrows indicate the probands. Genotypes for *ITK* are also shown. M, mutated. **(B)** Cranial T2-weighted MRI for P1. The high-intensity lesion in the right temporal lobe disappeared after 9 mo of anti-TB therapy (post-tx). **(C)** Surgical biopsy of an enlarged mesenteric lymph node of P1 showing central caseation on H&E staining and acid-fast bacilli (arrows) on Ziehl–Neelsen staining. **(D)** A thoracic CT scan of the lung of P3 showing tree-in-bud signs. Radiological improvement was observed after 4 mo of anti-TB therapy. **(E)** Variants detected by WES. **(F)** Sanger sequencing of the *ITK* variants. **(G)** Population genetics of *ITK*. The minor allele frequency (MAF) and CADD scores for all non-synonymous variants reported in the gnomAD database are shown. Three homozygous variants found in our private cohort are also shown. The CADD score of 35.0 for the c.496C>T (R166*) allele is well above the MSC of 20.7 (Kircher et al., 2014; Itan et al., 2016; horizontal dotted line). **(H)** Gene-level negative selection. Like other genes with mutations underlying AR IEI, *ITK* is not under negative selection, as shown by CoNeS (Rapaport et al., 2021). **(I)** Schematic representation of the ITK protein. PH, pleckstrin homology domain; SH, Src homology domain. Previously reported homozygous or compound heterozygous mutations are also shown.

**Figure S1.**
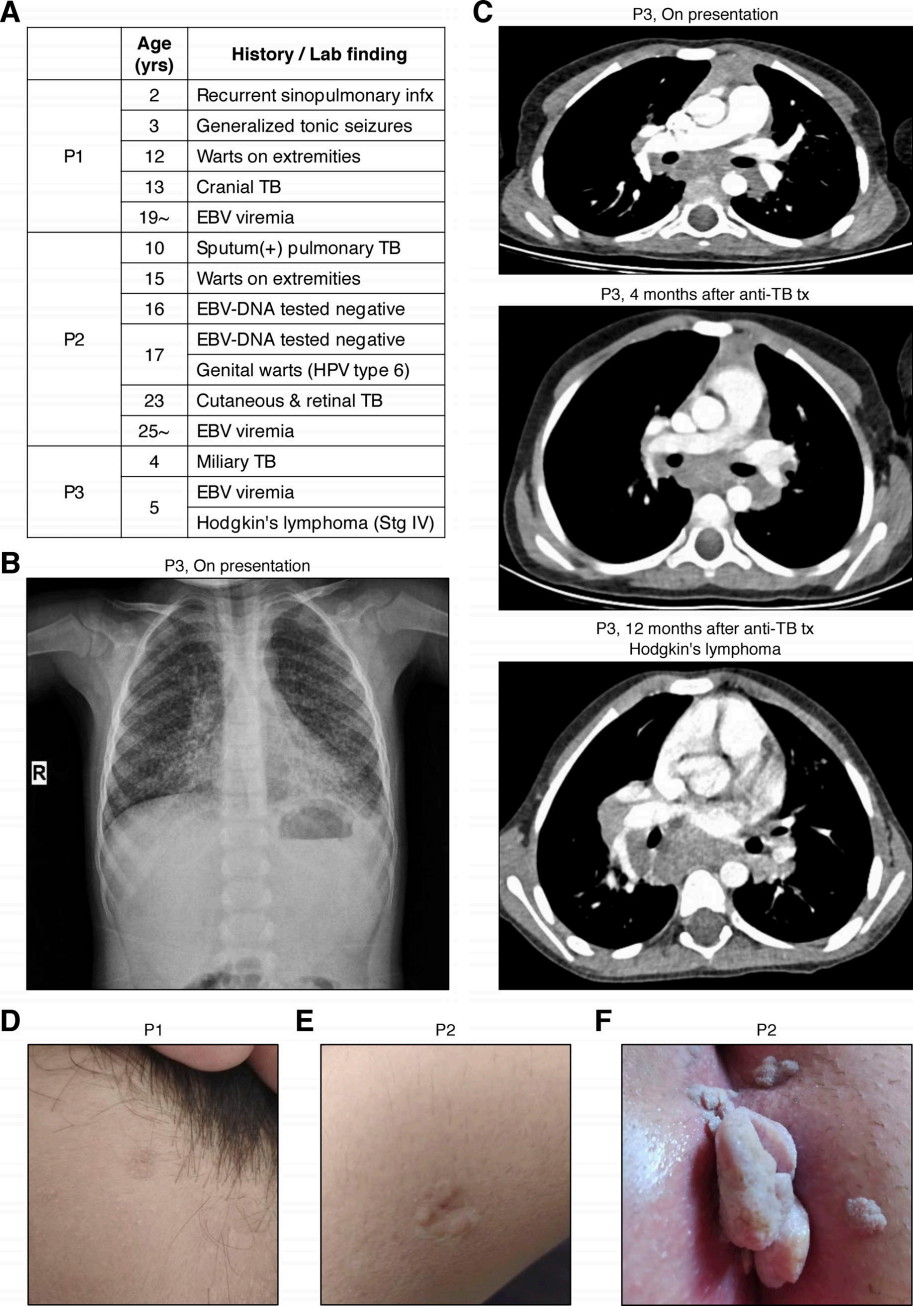
**Clinical findings. (A)** A table summarizing medical history and key laboratory findings related to infectious diseases in the three patients studied. **(B)** Chest x ray of P3 on presentation. **(C)** Thoracic CT scans of P3. **(D)** A common wart on the forehead of P1. **(E)** A common wart on the extremity of P2. **(F)** A genital wart in P2. Infx, infections; Stg, stage.

We studied two siblings from a consanguineous Iranian family and another patient from a consanguineous Turkish family (Fig. 1 A, and Fig. S1 A, Tables S1 and S2; and case reports in Materials and methods). The Iranian proband (P1) had disseminated TB (abdominal abscess, mesenteric lymphadenopathies, and cerebral abscess) caused by virulent *M. bovis* (different from BCG substrains, as proved by substrain-specific PCR) at the age of 13 yr (Fig. 1, B and C). Standard four-drug anti-TB treatment together with the subcutaneous injection of recombinant human IFN-γ resulted in complete remission. This patient is now 28 yr old and healthy. P1’s sister (P2) had sputum-confirmed pulmonary TB caused by *M. tb* at the age of 10 yr. She recovered on standard anti-TB therapy plus subcutaneous IFN-γ injections, but experienced cutaneous and retinal TB at the age of 23 yr. She is now 25 yr old and still on biweekly IFN-γ treatment. The Turkish patient (P3) developed miliary TB at the age of 4 yr. His chest computed tomography (CT) scans showed mediastinal lymphadenopathy and bilateral tree-in-bud lesions (Fig. 1 D and Fig. S1, B and C). Radiological and clinical improvement was observed after 12 mo of standard anti-TB therapy (Fig. 1 D). None of the three patients had experienced any adverse events after BCG vaccination (at the age of 3 d for P1 and P2 and 2 mo for P3).

### Virological manifestations

**Figure S2.**
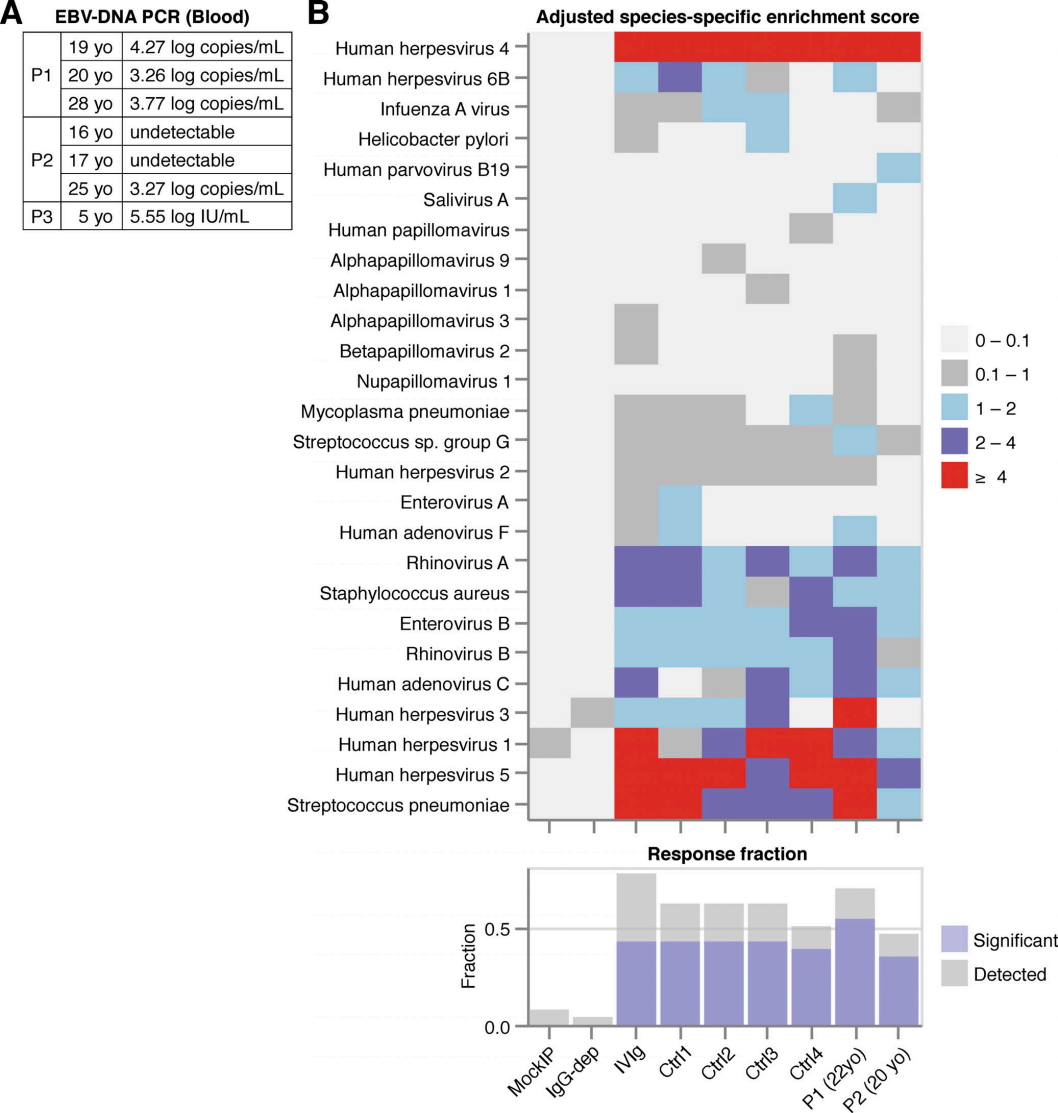
**Virological analysis. (A)** EBV-DNA viral load in the serum samples of P1, P2, and P3. yo, years old. **(B)** VirScan analysis of the serum samples of P1 and P2. Species are annotated based on the National Center for Biotechnology Information Taxonomy database (Schoch et al., 2020).

P1 and P2 had had common cutaneous warts on their foreheads and extremities since the ages of 12 and 15 yr, respectively (Fig. S1, D and E), whereas P3 had no warts. P2 had also had genital warts caused by human papillomavirus (HPV) type 6 since the age of 17 yr (Fig. S1 F). These cutaneous and genital warts responded to cryotherapy but recurred frequently. All patients had high levels of EBV DNA in the blood at their most recent examinations, at the ages of 28, 25, and 5 yr, respectively. P2 tested negative for EBV-DNA at the ages of 16 and 17 yr, whereas all the available samples from P1 and P3, taken from the ages of 19 and 5 yr onward, respectively, tested positive (Fig. S2 A). The ages of these two patients at primary infection with EBV are unknown. P1 and P2 were positive for VCA-IgG but negative for VCA-IgM and EBNA, whereas P3 was negative for VCA-IgM/IgG and EBNA. VirScan analysis for P1 and P2 (performed at the ages of 22 and 20 yr, respectively) also confirmed serological responses to common pathogens, including EBV (Fig. S2 B). P1 and P2 remained free from EBV-related disease at their most recent follow-up visits, at the ages of 28 and 25 yr, respectively, whereas P3 developed Hodgkin’s lymphoma at the age of 5 yr and recovered after receiving chemotherapy (Fig. S1 C).

### Private homozygous *ITK* mutations

We performed whole-exome sequencing (WES) for the three patients, hypothesizing an AR mode of inheritance with complete penetrance. Principal component analysis was consistent with the Iranian and Turkish ancestries of these patients. The homozygosity rates were 0.8% for P1, 1.9% for P2, and 2.1% for P3. Among the rare homozygous non-synonymous variants detected, the private nonsense *ITK* variant (c.496C>T, p.R166*) was the only variant common to P1 and P2, and the private missense *ITK* variant (c.1772T>C, p.M591T) was the only variant of a gene known to underlie IEI in P3 (Fig. 1 E). Sanger sequencing confirmed these *ITK* variants (Fig. 1 F). All healthy family members were heterozygous (Fig. 1, A and F). The variants were not found in the gnomAD database. The combined annotation-dependent depletion (CADD) scores were 35.0 and 28.1, well above the mutation significance cutoff (MSC) of 20.7 (Kircher et al., 2014; Itan et al., 2016; Fig. 1 G). *ITK* is not under strong negative selection according to CoNeS, a sequence-based metric for quantifying gene-level selection (Rapaport et al., 2021), like other genes with variants underlying AR IEI (Fig. 1 H). IL-2–inducible T cell kinase (ITK) protein, encoded by *ITK*, is a TEC (tyrosine kinase expressed in hepatocellular carcinoma) family tyrosine kinase expressed in T and NK lymphocytes and mast cells (Berg et al., 2005; Khurana et al., 2007; Iyer and August, 2008). The R166* variant is predicted to eliminate the SH2, SH3, and kinase domains, whereas the M591T variant is predicted to disrupt the kinase domain of ITK (Fig. 1 I). Patients with AR ITK deficiency present various hematological disorders due to uncontrolled EBV infection, including fatal infectious mononucleosis, lymphoma, lymphoproliferative disease, lymphomatoid granulomatosis, hemophagocytic lymphohistiocytosis, and dysgammaglobulinemia (Huck et al., 2009; Stepensky et al., 2011; Linka et al., 2012; Mansouri et al., 2012; Ghosh et al., 2014; Ghosh et al., 2018; Alme et al., 2015; Bienemann et al., 2015; Cipe et al., 2015; Çaǧdaş et al., 2017; Mo et al., 2019; Youssefian et al., 2019; Eken et al., 2019; Fang et al., 2019; Howe et al., 2019). We have also reported siblings with epidermodysplasia verruciformis caused by uncontrolled β-HPV infection (Youssefian et al., 2019). ITK-deficient patients presenting with mycobacterial diseases have never been reported, but a recent study of Itk-deficient mice showed impaired bacterial clearance and aggravated lung disease after intranasal *M. tb* infection (Huang et al., 2020). Collectively, familial and population genetics and the function of ITK in humans and mice suggested that AR ITK deficiency was the genetic etiology of TB in these three patients.

### Complete ITK deficiency

**Figure 2.**
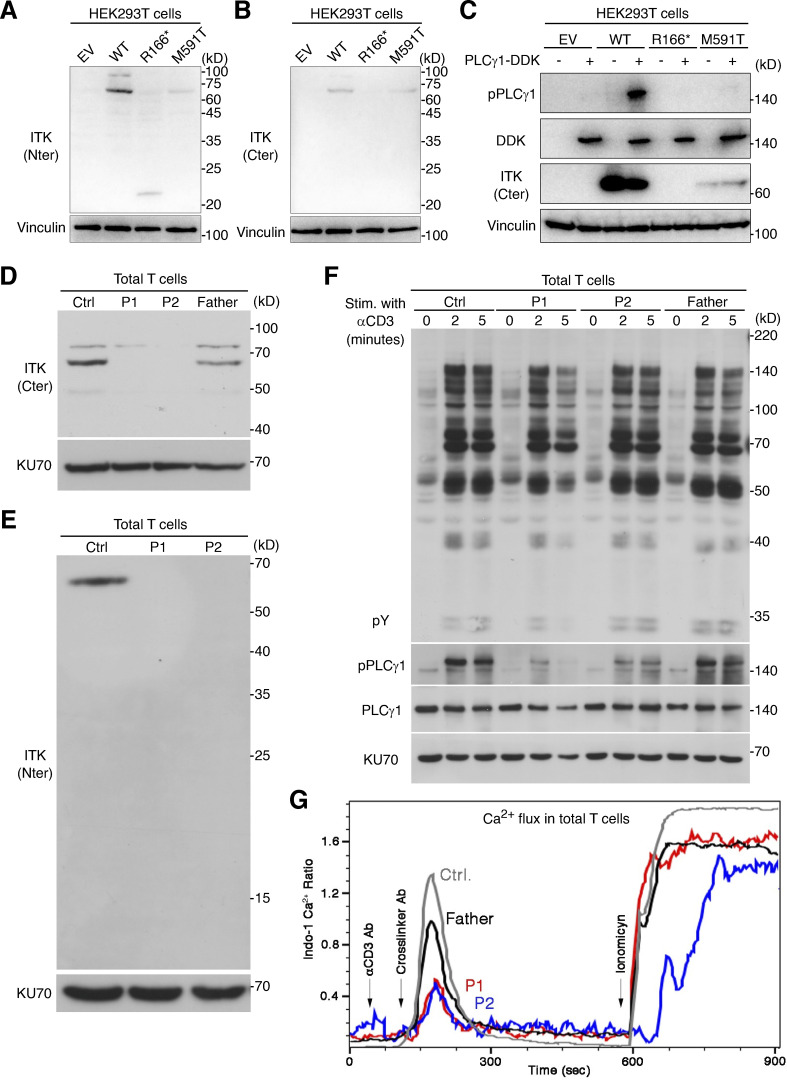
**Analysis of ITK expression and function. (A–C)** Studies of *ITK* alleles in an overexpression system. **(A and B)** Immunoblotting of the ITK protein with two different mAbs. **(C)** Phosphorylation of phospholipase C-γ1. **(D and E)** Immunoblotting for endogenous ITK protein with two different antibodies, on total T lymphocytes from P1, P2, their father, and one healthy control (Ctrl). **(F)** TCR signaling assay. Total T lymphocytes from P1, P2, their father, and one healthy control were analyzed by immunoblotting for general tyrosine phosphorylation (pY) and for the phosphorylation of phospholipase C-γ1 without stimulation or after 2 or 5 min of stimulation with anti-CD3 mAb. **(G)** Calcium (Ca^2+^) influx assay. Total T lymphocytes from P1, P2, their father, and one healthy control were stimulated by TCR crosslinking (with anti-CD3 and anti-IgG mAbs) or ionomycin. Intracellular Ca^2+^ concentration was determined with Indo-1. Representative results from at least two independent experiments are shown. Source data are available for this figure: SourceData F2.

**Figure S3.**
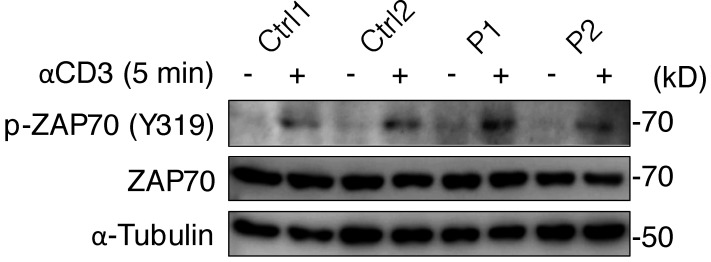
**Phosphorylation of ZAP70.** Total T lymphocytes from P1, P2, and two healthy controls (Ctrl) were analyzed by immunoblotting for phosphorylated ZAP70 (Y319) after 5 min of stimulation with anti-CD3 mAb. A representative result from two independent experiments is shown. Source data are available for this figure: SourceData FS3.

We first investigated the expression and function of the ITK protein in an overexpression system. Immunoblotting with two anti-ITK mAbs revealed that both mutants were produced in smaller amounts than the WT protein and that the protein encoded by the nonsense variant was truncated, with a molecular weight of 23 kD rather than 72 kD (Fig. 2, A and B). Phospholipase C gamma 1 (PLCγ1), a substrate directly phosphorylated by ITK, was phosphorylated by the WT protein but not by the variants, demonstrating a complete loss-of-function (LOF) for the patients’ alleles (Fig. 2 C). We then studied primary T lymphocytes from P1 and P2. Immunoblotting with two anti-ITK mAbs targeting N-terminal and C-terminal epitopes detected no full-length or truncated ITK protein (Fig. 2, D and E). The cells of the heterozygous father of P1 and P2 had ITK protein levels of about 50% of those in WT individuals (Fig. 2 D). Moreover, PLCγ1 phosphorylation levels in total T lymphocytes stimulated with anti-CD3 mAb were low for P2 and almost null for P1 (Fig. 2 F). By contrast, general levels of tyrosine residue phosphorylation and the levels of ZAP70 phosphorylation were similar in all individuals tested, suggesting that ITK deficiency selectively impairs PLCγ1 activation downstream from the crosslinked TCR complex without affecting TCR activation more broadly (Fig. 2 F and Fig. S3). Furthermore, the calcium flux of total T lymphocytes from P1 and P2 was low after TCR crosslinking with an anti-CD3 mAb (Fig. 2 G). Consistent with the ∼50% decrease in ITK protein levels observed on immunoblots, the T lymphocytes of the father of P1 and P2 also presented a partial decrease in calcium flux (Fig. 2 G). Finally, clinical investigations consistently revealed low levels of proliferation upon stimulation with PHA and anti-CD3 mAb in T lymphocytes from P1 and P2 (Table S1). Overall, these findings suggested that the three patients had AR complete ITK deficiency.

### Impaired T cell development in patients with inherited ITK deficiency

**Figure 3.**
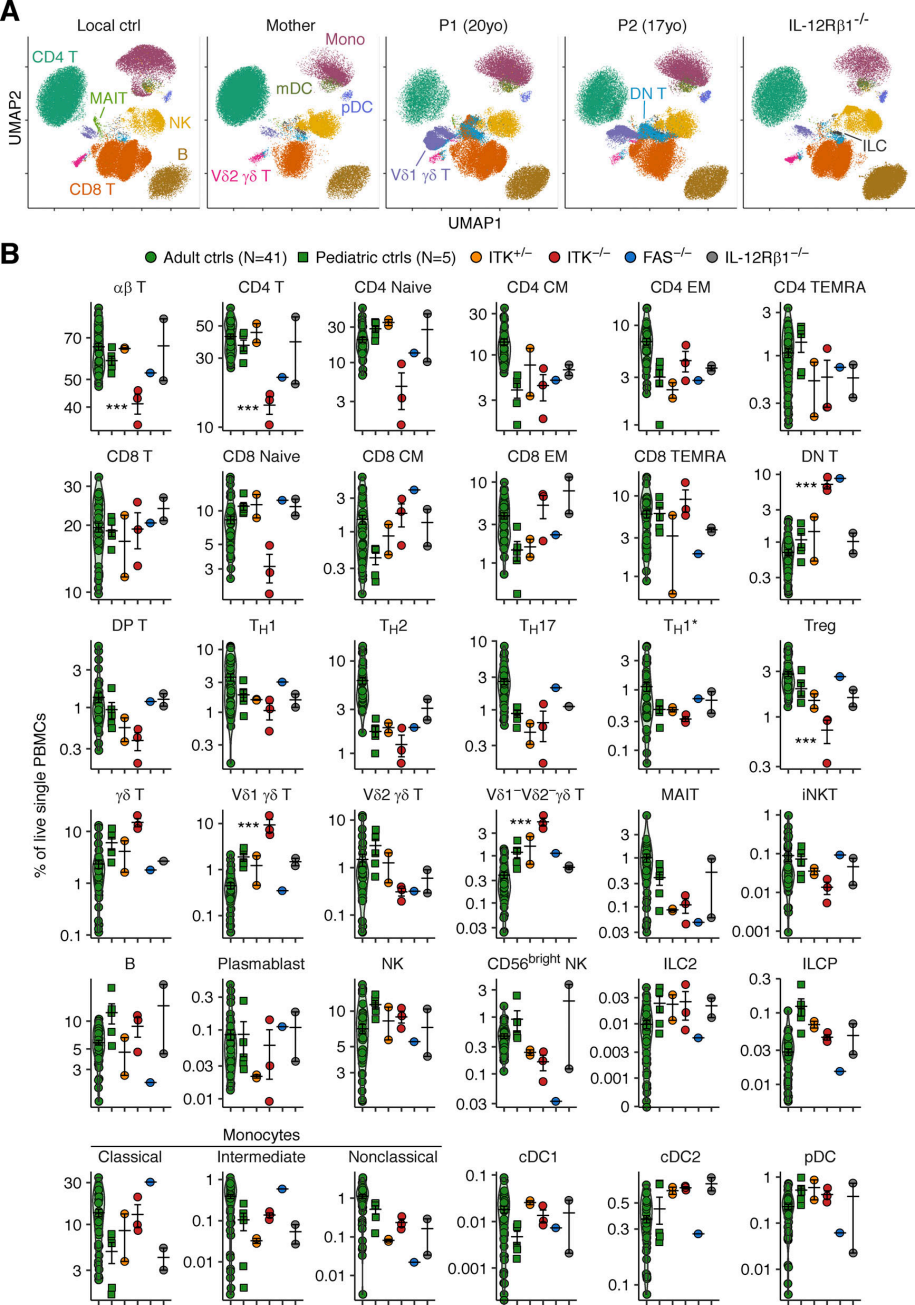
**Immunophenotyping of ITK-deficient leukocytes.** Freshly thawed PBMCs from P1, P2, P3 (obtained at the ages of 20, 17, and 4 yr, respectively), healthy members of their families, and adult and pediatric controls, together with two IL-12Rβ1-deficient patients and one FAS-deficient patient, were immunophenotyped by spectral flow cytometry. **(A)** UMAP visualization of cellular composition for 50,000 cells per individual. Cellular identity was determined by manual gating in FlowJo. **(B)** Abundance of leukocyte subsets. Experiments from two batches of experiments were compiled. Bars represent the mean and SEM. The statistical significance of differences was determined for comparisons of ITK-deficient patients vs. adult, pediatric, and familial controls combined. ***, P < 0.001, two-tailed Wilcoxon’s rank sum tests with FDR adjustment.

ITK expression is restricted to T and NK cells in the lymphoid lineage and mast cells in the myeloid lineage, in both humans and mice (Berg et al., 2005; Khurana et al., 2007; Iyer and August, 2008). We, therefore, hypothesized that ITK deficiency might impair anti-TB immunity by impeding the development of IFN-γ–producing T and NK lymphocytes. We comprehensively characterized the impact of inherited ITK deficiency on the development of leukocyte subsets by performing deep immunophenotyping on peripheral blood mononuclear cells (PBMCs) from P1, P2, and P3 (obtained at the ages of 20, 17, and 4 yr, respectively) and the heterozygous members of their families (Fig. 3). Blood samples from P2 tested negative for EBV-DNA at the ages of 16 and 17 yr, suggesting that any immunological abnormalities in this patient resulted from ITK deficiency rather than uncontrolled EBV viremia or related hematological disorders (Fig. S2 A). Mononuclear myeloid cells (monocytes and dendritic cells) and non-T lymphocytes (i.e., B cells, NK cells, and innate lymphoid cells) were present in normal numbers. Among innate-like adaptive T lymphocytes, the Vδ1^+^ and Vδ1^−^Vδ2^−^ γδ T lymphocyte populations displayed significant expansion, whereas Vδ2^+^ γδ T and mucosal-associated invariant T (MAIT) cells were unaffected. The numbers of invariant NK T (iNKT) lymphocytes were within the range of adult and pediatric controls, albeit at the lower end of this range, contrary to a previous report of a near-complete absence of iNKT lymphocytes (Huck et al., 2009). Despite the significantly lower numbers of blood CD4^+^ αβ T lymphocytes, consistent with the routine immunophenotyping data (Table S1 and S2), no decrease was observed in the counts for CD4^+^ T-helper subsets (Fig. 3 B). CD8^+^ αβ T lymphocyte counts were also normal, but naive CD8^+^ αβ T lymphocyte counts were moderately low, as in Itk-deficient mice (Broussard et al., 2006). Conversely, the CD4^−^CD8^−^ double-negative (DN) αβ T lymphocyte population was expanded (∼5% of circulating leukocytes), similar to the FAS-deficient patient simultaneously studied who manifested as an autoimmune lymphoproliferative syndrome (ALPS). Itk-deficient mice present CD4^+^ αβ T lymphocytopenia, with a concomitant expansion of the DN αβ T lymphocyte population in peripheral lymph nodes (Liao and Littman, 1995) and an expansion of γδ T lymphocytes in the thymus, spleen, and liver (Felices et al., 2009). Overall, human ITK deficiency impairs the development of CD4^+^ αβ T lymphocytes while facilitating the development of DN αβ T and Vδ1^+/−^Vδ2^−^ γδ T lymphocytes.

### Atypical phenotypes of aberrantly expanded ITK-deficient DN αβ T lymphocytes

**Figure 4.**
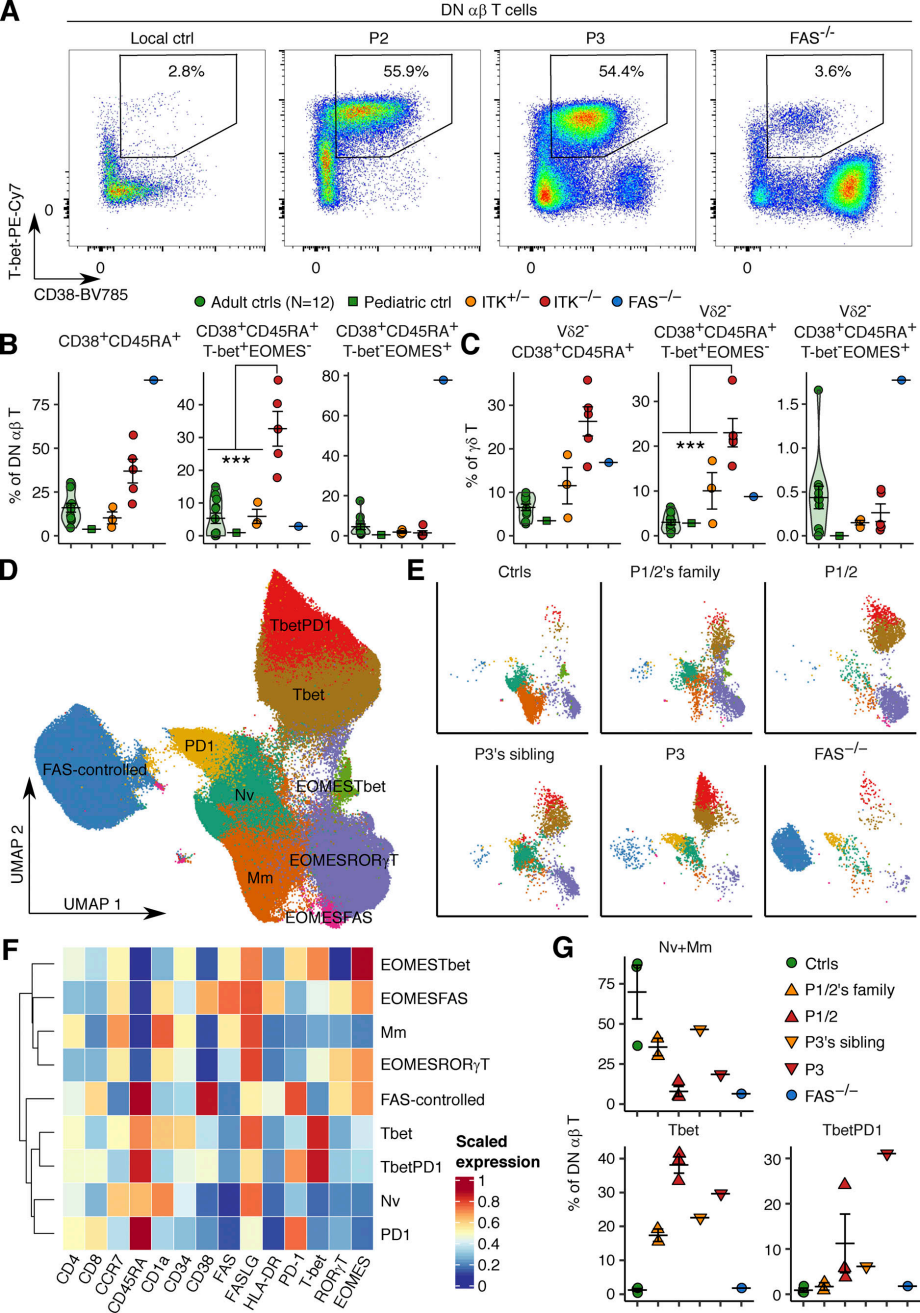
**Atypical phenotypes of ITK-deficient DN αβ and γδ T lymphocytes.** PBMCs from P1 (obtained at the ages of 18 and 19 yr), P2 (obtained at the age of 16 yr, free from EBV viremia), P3 (at the age of 4 yr), heterozygous members of their families, one patient with a homozygous LOF (R29C) mutation of *ITK* with EBV viremia but no history of TB, and one FAS-deficient patient were studied by flow cytometric immunophenotyping. **(A)** A representative plot of CD38 and T-bet expression. **(B and C)** Phenotypes of (B) DN αβ T and (C) Vδ2^−^ γδ T lymphocytes. Results from three batches of experiments are compiled. **(D–G)** FlowSOM-guided clustering analysis. **(D and E)** UMAP plots of (D) all DN αβ T lymphocytes and (E) 2,500 cells per group. Equal numbers of cells were randomly sampled from each individual in each group. **(F)** Heatmap of scaled median expression levels. **(G)** Cluster abundance. In B, C, and G, the bars represent the mean and SEM. In B and C, statistical significance was determined for differences between ITK-deficient patients and adult, pediatric, and familial controls combined. ***, P < 0.001, two-tailed Wilcoxon’s rank sum tests with FDR adjustment.

We investigated the origin of the expanded DN αβ T lymphocytes by performing T cell–focused immunophenotyping on PBMCs from P1 (obtained at the ages of 18 and 19 yr), P2 (at the age of 16 yr, free from EBV viremia), P3 (at the age of 4 yr), and the heterozygous members of their families. We also analyzed a patient homozygous for the R29C variant, a well-characterized LOF mutant of *ITK* (Rigby et al., 2009), who had EBV viremia in the absence of hematological disorders or a history of TB (P4). By studying this patient, we were able to explore the phenotypic alterations caused by ITK deficiency in the absence of a history of TB disease. We also studied one FAS-deficient ALPS patient, because an ITK-deficient patient with ALPS-like clinical manifestations has been reported (Wallace et al., 2020). We found a unique CD38^+^CD45RA^+^T-bet^+^EOMES^−^ phenotype in the DN αβ T lymphocytes in all five samples from four ITK-deficient patients, different from the previously reported CD38^bright^CD45RA^+^T-bet^-^EOMES^+^ phenotype in FAS-deficient DN αβ T lymphocytes (Rensing-Ehl et al., 2014; Maccari et al., 2020; Fig. 4, A and B). The Vδ2^−^ γδ T lymphocytes of the ITK-deficient patients also had the same CD38^+^CD45RA^+^T-bet^+^EOMES^−^ phenotype (Fig. 4 C). We further investigated the role of ITK in governing the development of DN αβ T lymphocytes by analyzing manually gated DN αβ T lymphocytes by unsupervised clustering with FlowSOM (Fig. 4, D–F). DN αβ T lymphocytes from the FAS-deficient patient formed a distinct cluster characterized by a unique CD38^bright^CD45RA^+^PD-1^+^T-bet^-^RORγT^+^EOMES^+^ phenotype. Conversely, ITK-deficient DN αβ T lymphocytes formed another distinct cluster, which was in turn subdivided into two subclusters “Tbet” and “TbetPD1” (Fig. 4, E and G). These two subclusters shared high levels of T-bet expression (Fig. 4 F). In addition, the “TbetPD1” and “Tbet” subclusters had CD34^dim^CD38^+^CD1a^−^ and CD34^+^CD38^dim^CD1a^+^ phenotypes, thus resembling the DN2 and DN3 stages of human T cell development in the thymus, respectively (Dik et al., 2005). Collectively, our analysis indicates that inherited ITK deficiency diverts the development of normal CD4^+^ αβ T lymphocytes to atypical DN αβ T lymphocytes, which are different from the pathogenic DN αβ T lymphocytes seen in FAS-deficient individuals.

### Expansion of γδ and CD4^−^CD8^−^ αβ T lymphocytes in Itk-deficient mice

**Figure 5.**
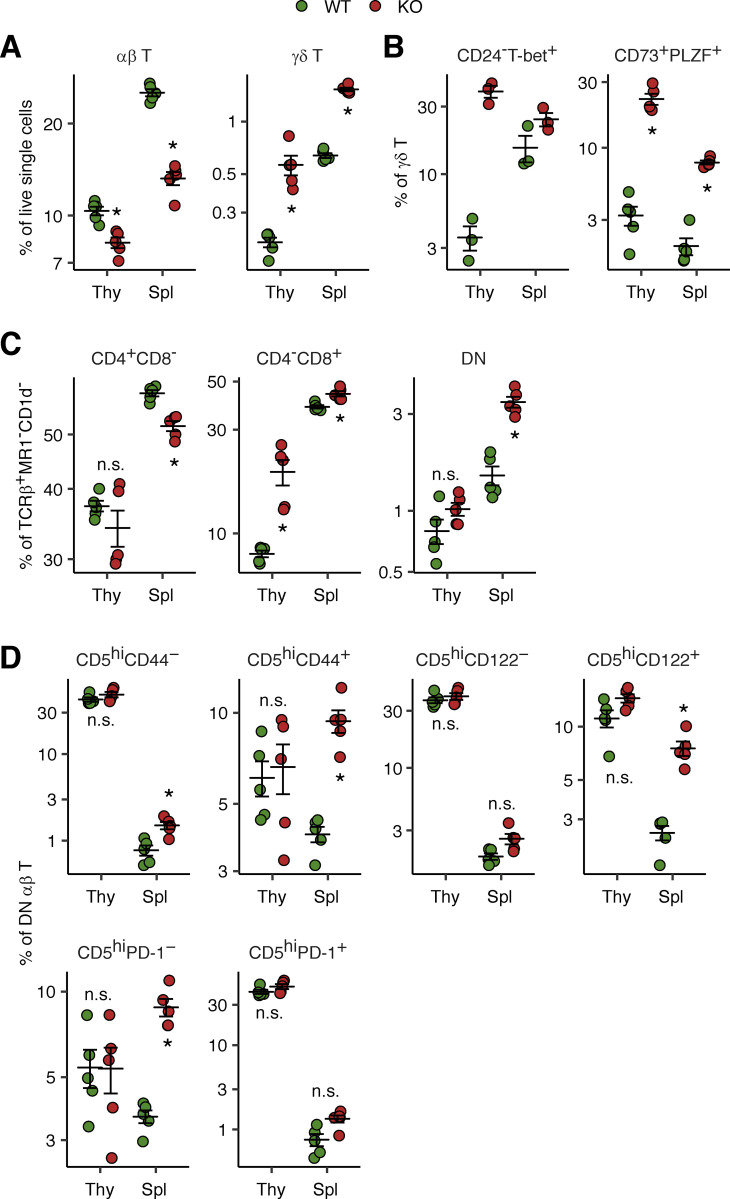
**Expansion of the γδ and DN αβ T cell populations in Itk-deficient mice.** Five WT and Itk-deficient mice (C57BL/6 background) were studied in two batches of experiments. **(A)** Frequencies of αβ and γδ T cells in the thymus (Thy) and spleen (Spl). **(B)** Phenotypes of γδ T cells. CD24 staining was performed for only three of the five mice. **(C)** αβ T cell subsets. **(D)** Phenotypes of DN αβ T cells. Bars represent the mean and SEM. Statistical significance was determined for differences between WT and Itk-deficient mice. n.s., not significant. *, P < 0.05, two-tailed Wilcoxon’s rank sum tests with FDR adjustment.

We investigated the origin of the DN αβ T and Vδ2^−^ γδ T lymphocyte populations expanded in the peripheral bloodstream of ITK-deficient patients by studying the thymus and spleen of Itk-deficient mice. We found high CD3^+^γδTCR^+^ γδ T lymphocyte counts in the thymus and low CD3^+^TCRβ^+^ αβ T lymphocyte counts in the spleen of Itk-deficient mice (Fig. 5 A). Thymic γδ T lymphocytes from Itk-deficient mice had a more mature (CD24^−^) phenotype with high levels of T-bet expression, whereas this phenotype was not evident in the spleen (Fig. 5 B). Furthermore, Itk-deficient γδ T lymphocytes in the thymus and spleen had an enhanced CD73^+^PLZF^+^ phenotype (Fig. 5 B). CD73 is a marker of thymic CD4^−^CD8^−^ progenitors committed to a γδ T cell fate, whereas PLZF is a transcription factor responsible for the innate-like phenotype of subsets of both αβ and γδ T lymphocytes (Kreslavsky et al., 2009; Coffey et al., 2014). An expansion of DN αβ T lymphocytes (excluding MR1- and CD1d-tetramer–positive cells) was observed in the periphery (spleen), but not in the thymus of Itk-deficient mice (Fig. 5 C). We found that the DN αβ T lymphocytes in the spleen of Itk-deficient mice displayed enhanced CD5 expression, and these CD5^hi^ cells had a CD44^+^CD122^+^PD-1^−^ phenotype (Fig. 5 D). CD5 is a marker of postselection thymocytes that is also expressed on CD4^−^CD8^−^ postselection intraepithelial lymphocyte precursors (IELPs) in the thymus. The CD44^+^CD122^+^ phenotype is linked to a mature and activated state in thymocytes (Hanke et al., 1994; Gangadharan, et al., 2006; McDonald et al., 2014), and this phenotype is particularly marked in CD8^+^ T lymphocytes in Itk-deficient mice (Broussard et al., 2006). CD122^+^ IELP-like cells are also present in the spleen (McDonald et al., 2014). Unlike γδ T lymphocytes, Itk-deficient DN αβ T cells from the thymus and spleen displayed no alteration to T-bet expression. Collectively, inherited ITK deficiency promotes γδ lineage commitment and maturation in the thymus and, therefore, the accumulation of γδ T cells in the periphery, also causing DN αβ T cells resembling thymic IELPs to accumulate in the periphery.

### Impaired IFN-γ production by ITK-deficient T lymphocytes

**Figure 6.**
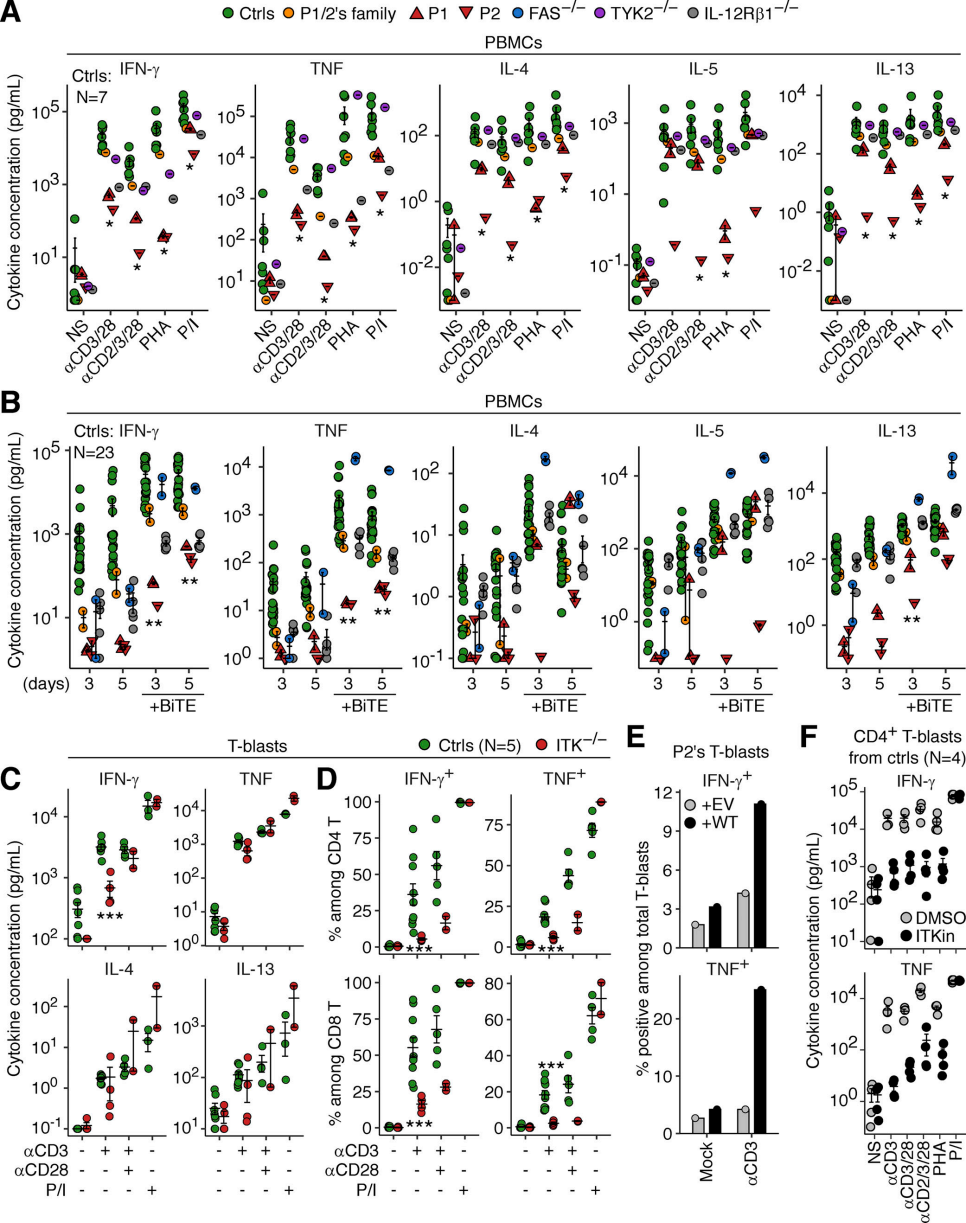
**Impaired production of IFN-γ and TNF by ITK-deficient T lymphocytes. (A and B)** PBMCs from P1 and P2 (obtained at the ages of 19 and 18 yr, respectively) and their father were stimulated for (A) 18 h or (B) 3 or 5 d with IL-2. Secreted cytokine levels were determined in LEGENDplex assays. Technical duplicates were prepared for (A) P1 or (B) P1, P2, and their father (except P2 for 3 d). **(C and D)** Expanded T cell blasts (T-blasts) from P1 and P2 were stimulated for 2 h with various reagents and then for an additional 5 h in the presence of a secretion inhibitor. Secreted cytokines were determined with the LEGENDplex assay, and intracellular cytokine levels were determined by flow cytometry. Technical duplicates were prepared for the unstimulated and anti-CD3 mAb-conjugated bead-stimulated samples. **(E)** Lentiviral rescue. P2’s T-blasts underwent lentiviral transduction with the EV or the WT *ITK* CDS. Cells were selected on puromycin. Cells were either left unstimulated or stimulated with anti-CD3 mAb-conjugated beads for 5 h. Cytokine production was assessed by flow cytometry. **(F)** Pharmacological ITK inhibition. Magnetically enriched CD4^+^ T-blasts from four healthy donors were incubated for 1 h with DMSO or an ITK inhibitor (BMS509744) and were then stimulated for 6 h. Secreted cytokines were determined in LEGENDplex assays. In A–D and F, bars represent the mean and SEM. In A–D, statistical significance was determined for differences between the two ITK-deficient patients on the one hand, and local and familial controls on the other. *, P < 0.05; **, P < 0.01; ***, P < 0.001, and two-tailed Wilcoxon’s rank sum tests with FDR adjustment.

**Figure S4.**
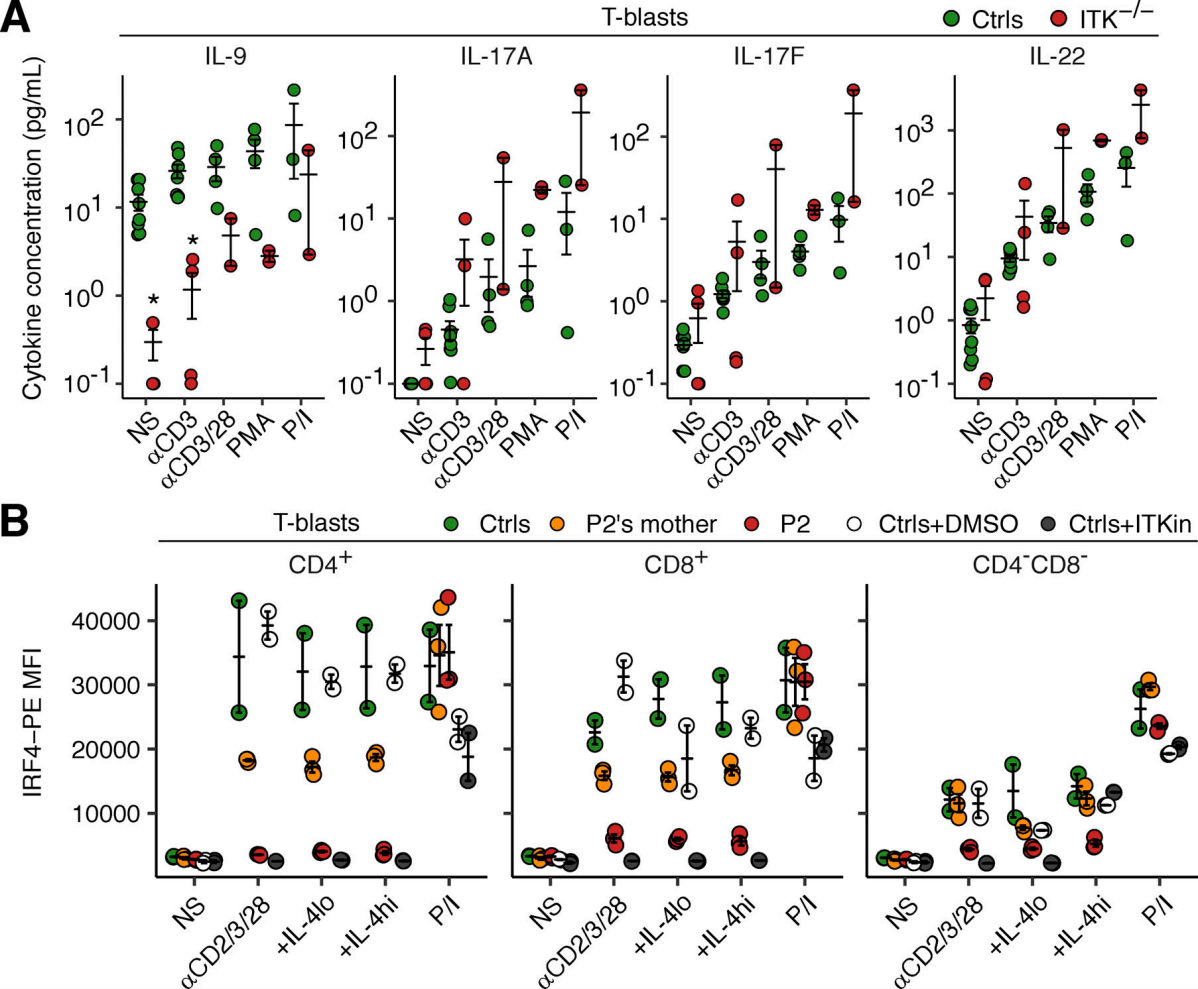
**Impaired production of IL-9 by ITK-deficient T lymphocytes. (A)** T-blasts from P1 and P2 were stimulated for 2 h with various reagents. Secreted cytokines were determined with the LEGENDplex assay. Technical duplicates were prepared for the unstimulated and anti-CD3 mAb-conjugated bead-stimulated samples. **(B)** Expression of IRF4. T-blasts from P2, her mother, and local controls were either left unstimulated or were stimulated with an anti-CD2/CD3/CD28 mAb cocktail for 24 h and analyzed by flow cytometry. For pharmacological ITK inhibition, T-blasts from controls were cultured in the presence of DMSO or an ITK inhibitor (BMS509744). Bars represent the mean and SEM. Statistical significance was determined for differences between the two ITK-deficient patients on the one hand, and local and familial controls on the other. *, P < 0.05, two-tailed Wilcoxon’s rank sum tests with FDR adjustment. Representative results from two independent experiments are shown.

We characterized the molecular basis of TB in ITK-deficient patients by studying PBMCs from P1 and P2 (obtained at the ages of 19 and 18 yr, respectively) and their father, and from FAS-deficient, TYK2-deficient, and IL-12Rβ1-deficient patients. P1’s leukocytes displayed impaired secretion of IFN-γ and tumor necrosis factor (TNF) upon stimulation with anti-CD3/CD28 mAb-conjugated beads, anti-CD2/CD3/CD28 mAb cocktail, and PHA, whereas PMA and ionomycin (referred to hereafter as P/I) induced normal levels of secretion, as shown by multiplex ELISA (Fig. 6 A). The defects of P2’s cells were more profound than those of P1’s cells. Leukocytes from P1 and P2 displayed impaired secretion of IFN-γ and TNF following stimulation with blinatumomab, an artificial CD3/CD19-bispecific T lymphocyte engager (BiTE) enforcing immunological synapse formation between autologous T and B lymphocytes (Fig. 6 B). Secretion of the T_H_2 cytokines IL-4/5/13 was also reduced, but to a lesser extent than that of IFN-γ and TNF. To confirm the observation of impaired IFN-γ and TNF secretion, we then studied expanded T cell blasts (T-blasts) from P1 and P2. Multiplex ELISA revealed an impairment of IFN-γ secretion, but not of the secretion of TNF, IL-4, IL-13, IL-17A, and IL-17F, by T-blasts from P1 and P2 in response to anti-CD3 mAb-conjugated beads (Fig. 6 C and Fig. S4 A). IL-9 secretion was also reduced, consistent with a previous report demonstrating the essential role of ITK in the TCR crosslinking-triggered induction of IRF4 and the differentiation of T_H_9 cells (Gomez-Rodriguez et al., 2016; Fig. S4 B). Flow cytometry showed that the production of IFN-γ and TNF was impaired (Fig. 6 D). Furthermore, the lentiviral transduction of T-blasts from P2 with WT *ITK*, but not with an empty vector (EV), enhanced the anti-CD3 antibody-stimulated production of IFN-γ and TNF, as determined by flow cytometry (Fig. 6 E). Finally, the pharmacological inhibition of ITK reduced the secretion of IFN-γ and TNF in CD4^+^ T-blasts from healthy donors stimulated with anti-CD3, anti-CD3/CD28, anti-CD2/CD3/CD28 mAbs, and PHA, as shown by ELISA, whereas P/I-induced secretion was normal (Fig. 6 F). Thus, ITK deficiency partially impairs, but not abolishes, IFN-γ production by T lymphocytes.

### Impaired IFN-γ production by ITK-deficient lymphocytes in response to mycobacteria

**Figure 7.**
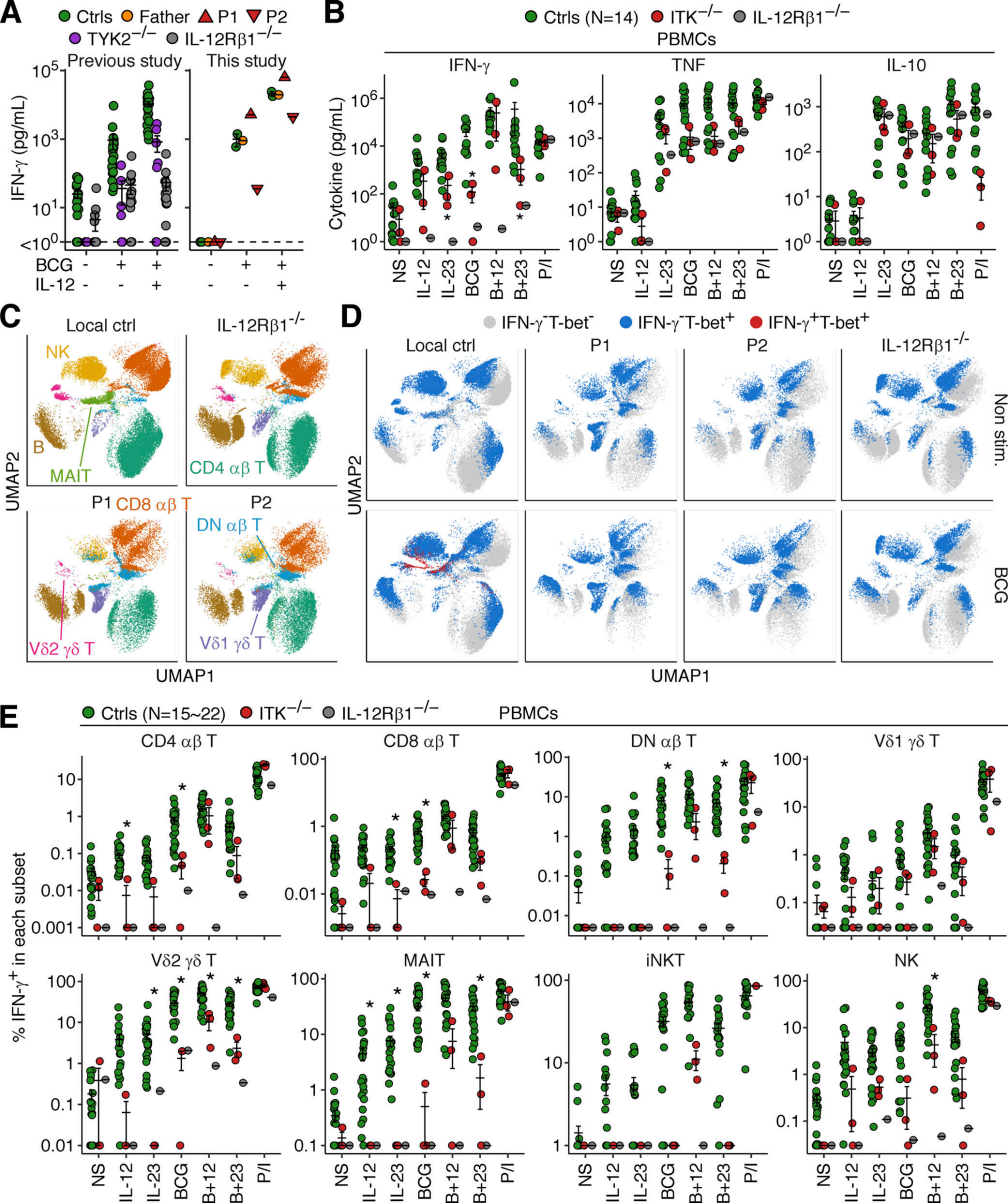
**Impaired IFN-γ production in response to mycobacteria.** The blood samples of P1 and P2 were obtained at the ages of 19 and 16 yr, respectively. **(A)** Whole-blood BCG infection assay. One local control and one travel control were grouped as controls. Secreted IFN-γ levels were determined by ELISA. For comparison, data from a previous study (Boisson-Dupuis et al., 2018) are also shown. **(B–E)** PBMC BCG infection assay. Freshly thawed PBMCs from P1, P2, P4, one IL-12Rβ1-deficient patient, and healthy local controls were stimulated for 40 h with various combinations of IL-12, IL-23, and BCG, and were then incubated with a cytokine secretion inhibitor for 8 h. **(B)** Secreted cytokine levels, as determined in LEGENDplex assays. **(C and D)** UMAP visualization of (C) manually gated cell subsets without stimulation and (D) IFN-γ and T-bet expression in non-stimulated and BCG-stimulated cells, as determined by flow cytometry. Results for 10,000 randomly downsampled cells per sample are visualized. **(E)** IFN-γ production by lymphocyte subsets. In A, B, and E, bars represent the mean and SEM. In B and E, statistical significance was determined for comparisons of the three ITK-deficient patients with local controls. *, P < 0.05, two-tailed Wilcoxon’s rank sum tests with FDR adjustment.

**Figure S5.**
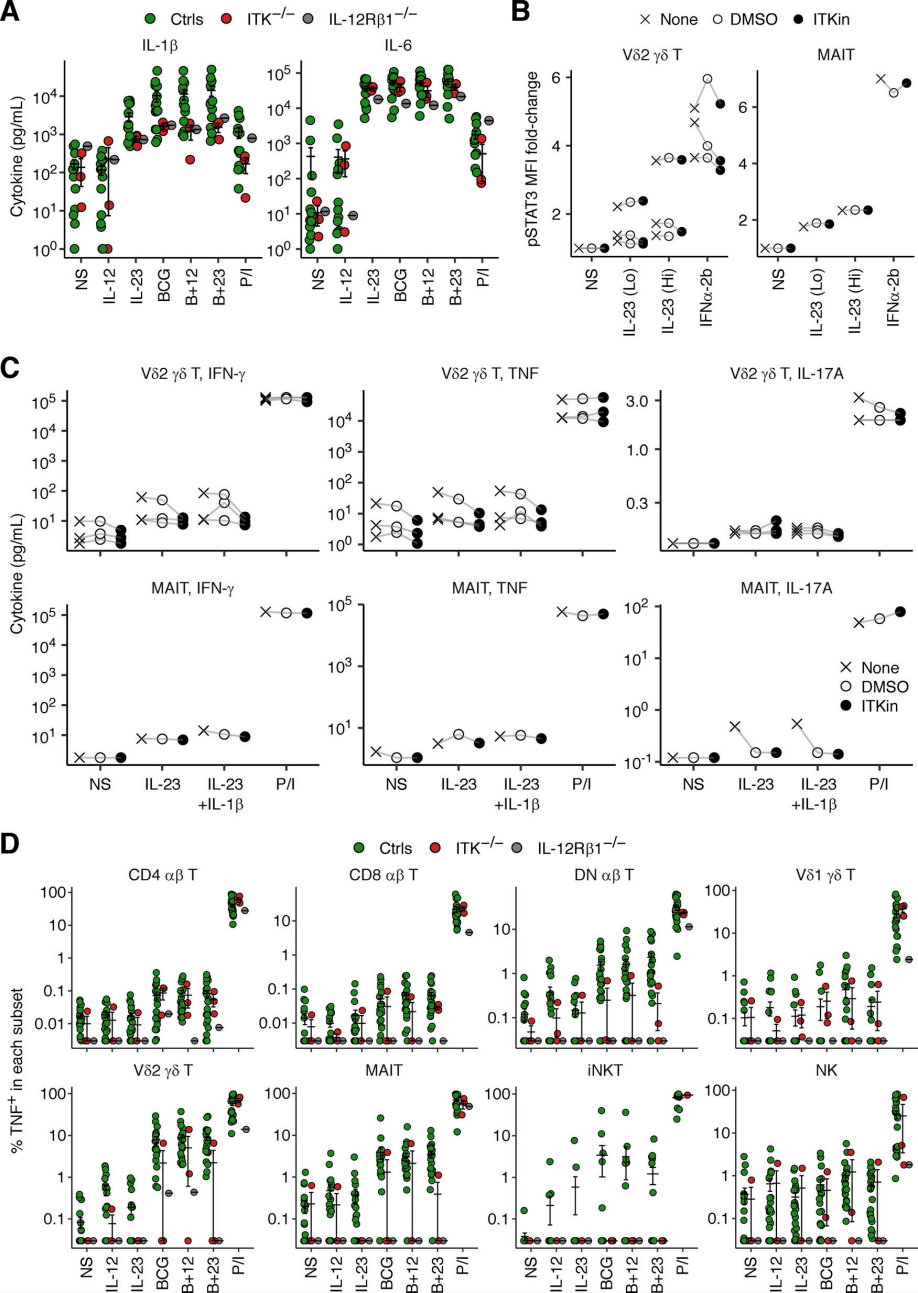
**Analysis of the role of ITK in IL-23-mediated antimycobacterial immunity. (A)** Freshly thawed PBMCs from P1 (age 19 yr), P2 (age 16 yr), P4, one IL-12Rβ1-deficient patient, and healthy local controls, were stimulated for 40 h with various combinations of IL-12, IL-23, and BCG, and were then incubated with a cytokine secretion inhibitor for 8 h. Secreted cytokine levels were determined with a LEGENDplex assay. **(B and C)** Analysis of IL-23 signaling. Vδ2^+^ γδ T and MAIT cells were sorted from healthy donors and subjected to pretreatment with DMSO or an ITK inhibitor (BMS509744). **(B)** STAT3 phosphorylation levels after stimulation with IL-23 or IFNα-2b for 30 min, as determined by flow cytometry. **(C)** Cytokine secretion, as measured by a LEGENDplex assay, after stimulation with IL-23, with or without IL-1β, for 48 h. **(D)** TNF production by lymphocyte subsets, as determined by flow cytometry, as in A. In A and D, bars represent the mean and SEM.

We tested the hypothesis that ITK deficiency impairs the production of IFN-γ in response to mycobacterial stimulation by studying whole-blood samples from P1 and P2 (obtained at the ages of 19 and 16 yr, respectively), their father, and a local control. Upon stimulation with live BCG alone or BCG plus IL-12, leukocytes from P2 displayed impaired IFN-γ secretion (Fig. 7 A), whereas no such impairment was observed for the leukocytes from P1. We then studied PBMCs from P1, P2, P4, one IL-12Rβ1-deficient patient, and local controls. Cells were stimulated with combinations of BCG, IL-12, and IL-23 for 40 h and then with a secretion inhibitor for 8 h. Multiplex ELISA revealed low levels of IFN-γ secretion in the three ITK-deficient patients upon stimulation with IL-23, BCG, or BCG plus IL-23, whereas the response to P/I was normal (Fig. 7 B). The secretion of other cytokines, including TNF, IL-10, IL-1β, and IL-6, was normal (Fig. 7 B and Fig. S5 A). Moreover, intracellular flow cytometry showed impaired IFN-γ production by Vδ2^+^ γδ T and MAIT cells upon stimulation with BCG, IL-23, or both (Fig. 7, C–E), suggesting that these innate-like adaptive T lymphocytes account for the impairment of IL-23–dependent IFN-γ production. By contrast, pharmacological ITK inhibition did not affect the phosphorylation of STAT3 or the secretion of IFN-γ, TNF, and IL-17A triggered by IL-23 in sorted Vδ2^+^ γδ T and MAIT cells from healthy donors, suggesting that inherited ITK deficiency disrupts the functions of these cells, but not their IL-23–responsive signaling pathway per se (Fig. S5, B and C). Furthermore, CD4^+^, CD8^+^, and DN αβ T lymphocytes displayed impaired IFN-γ production in response to BCG (Fig. 7 E). Vδ1^+^ γδ T lymphocytes displayed normal IFN-γ production in all conditions tested, possibly partly compensating for the overall reduction of IFN-γ production (Fig. 7, B and E). Conversely, TNF production was normal in response to IL-23, BCG, or BCG plus IL-23, consistent with the normal levels of total TNF secretion (Fig. 7 B and Fig. S5 D). Thus, inherited ITK deficiency impairs IFN-γ production by CD4^+^/CD8^+^/DN αβ T, Vδ2^+^ γδ T, and MAIT cells.

### Human ITK governs the cytotoxic functions of innate-like adaptive T lymphocytes

**Figure 8.**
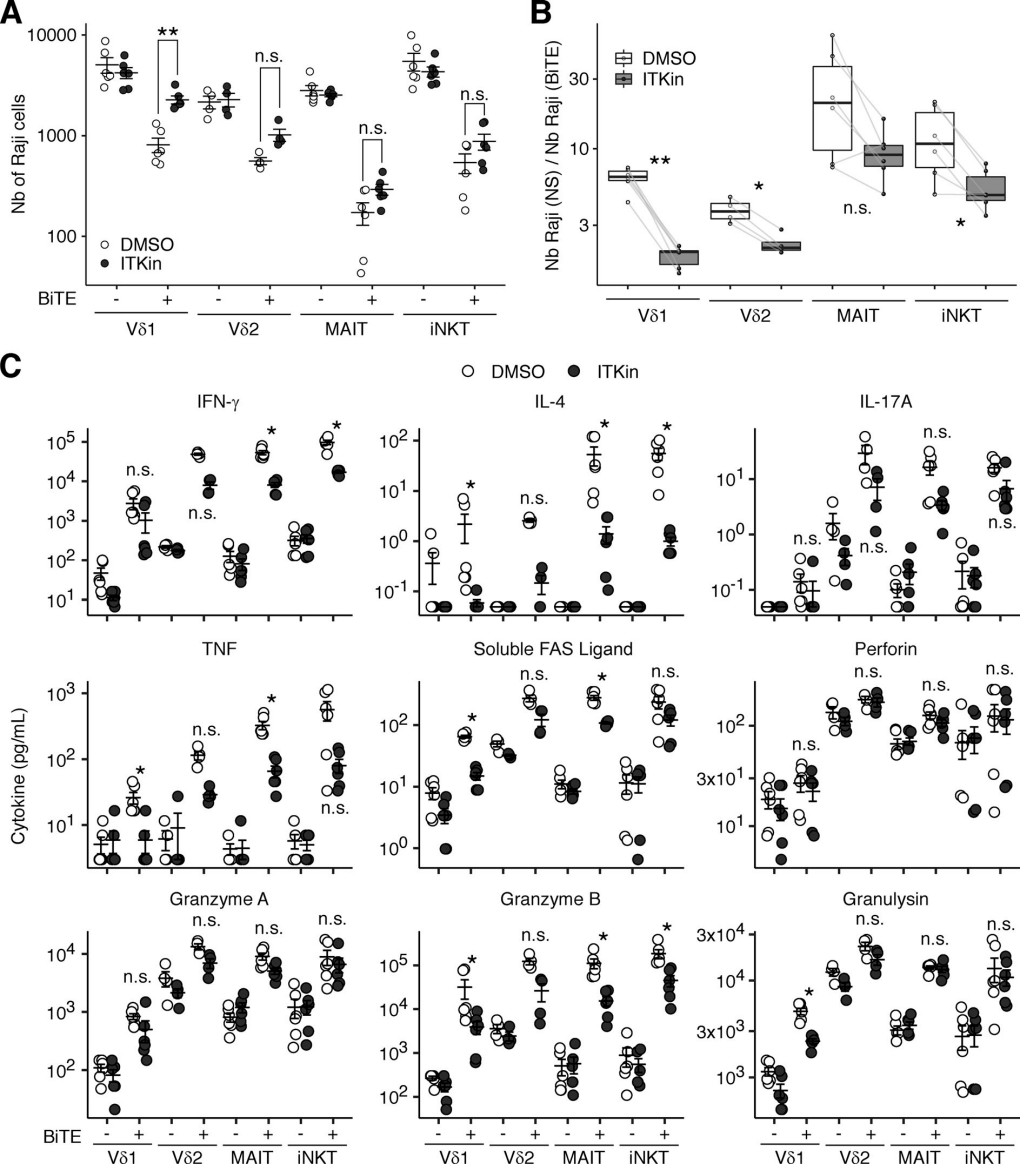
**Human ITK is required for optimal cytotoxicity in innate-like adaptive T lymphocytes.** Innate-like adaptive T lymphocyte subsets were sorted from two to three healthy donors, expanded for 2 wk, and cocultured with CFSE-stained Raji cells with or without BiTE in the presence of DMSO or an ITK inhibitor (BMS509744). After 24 h of coculture, the supernatants were collected and analyzed with a LEGENDplex assay. The cells were acquired with an Attune NxT automated flow cytometry instrument to quantify the remaining CFSE^+^ Raji cells in equal volumes of sample. **(A)** Absolute numbers of Raji cells per well. **(B)** The ratio of the numbers of Raji cells in wells with and without BiTE. A higher ratio indicates more potent BiTE-induced Raji cell killing. **(C)** Secreted cytokines and cytotoxic effector molecules. Bars represent the mean and SEM. Statistical significance was determined for comparisons between samples with or without ITK inhibition. n.s., not significant. *, P < 0.05; **, P < 0.01, and two-tailed Wilcoxon’s rank sum tests with FDR adjustment. Representative results from two independent experiments are shown.

ITK-deficient patients are susceptible to certain chronic viral infections, such as EBV and HPV infections, perhaps due to impaired T cell cytotoxicity. As we observed defective IFN-γ production, particularly by ITK-deficient Vδ2^+^ γδ T and MAIT cells in the BCG infection assay in vitro, we investigated whether human ITK was required for optimal cytotoxicity in innate-like adaptive T lymphocytes. We cocultured Raji cells (a human EBV-positive Burkitt's lymphoma cell line) and sorted Vδ1^+^ γδ T, Vδ2^+^ γδ T, MAIT, and iNKT lymphocytes from healthy donors with or without pharmacological ITK inhibition and BiTE-triggered synapse formation. Raji cells were prestained with CFSE for subsequent quantification by flow cytometry. By counting the Raji cells surviving after 24 h of coculture, we found that pharmacological ITK inhibition partially prevented BiTE-triggered Raji cell killing by Vδ1^+^ and Vδ2^+^ γδ T lymphocytes, but not by MAIT or iNKT cells (Fig. 8 A). However, when we calculated the ratio of the number of surviving Raji cells in wells with BiTE to that without BiTE to eliminate the between-donor variability, the impact of pharmacological ITK inhibition became statistically significant for Vδ1^+^ γδ T, Vδ2^+^ γδ T, and iNKT lymphocytes (Fig. 8 B). We also determined secreted cytokine and cytotoxic effector molecule levels by multiplex ELISA. Pharmacological ITK inhibition partially reversed the BiTE-triggered enhancement of IFN-γ secretion by Vδ2^+^ γδ T, MAIT, and iNKT lymphocytes (Fig. 8 C). The secretion of TNF, IL-4, soluble FAS ligand, granzyme A, and granzyme B by Vδ2^+^ γδ T and MAIT cells was also partially inhibited by pharmacological ITK inhibition. By contrast, ITK inhibition did not affect the secretion of perforin by any of the cell types tested. Thus, human ITK is required for the optimal killing of virus-transformed cells and the secretion of cytotoxic mediators by innate-like adaptive T lymphocytes, potentially accounting for the predisposition to chronic viral diseases in patients with ITK deficiency.

## Discussion

EBV-driven hematological disorders are characteristic of inherited ITK deficiency in humans (Huck et al., 2009; Stepensky et al., 2011; Linka et al., 2012; Mansouri et al., 2012; Ghosh et al., 2014; Ghosh et al., 2018; Alme et al., 2015; Bienemann et al., 2015; Cipe et al., 2015; Çaǧdaş et al., 2017; Mo et al., 2019; Youssefian et al., 2019; Eken et al., 2019; Fang et al., 2019; Howe et al., 2019). However, 4 of the 23 reported ITK-deficient patients from 16 kindreds had no overt EBV-related disease on presentation. Serwas et al. (2014) reported a 17-yr-old Turkish patient with persistent CD4^+^ T lymphocytopenia and recurrent pulmonary infections. However, this patient subsequently presented leiomyoma and a high EBV load in the blood. Çaǧdaş et al. (2017) reported a Turkish patient with recurrent febrile pneumonia who died from respiratory insufficiency at the age of 3 yr, with no documentation of EBV load. Our group reported a 44-yr-old Iranian patient with EV and EBV viremia (Youssefian et al., 2019). Finally, Wallace et al. (2020) reported a 13-yr-old Iraqi patient presenting with ALPS. The blood of this patient tested negative for EBV-DNA, but in situ hybridization revealed the presence of EBV-encoded small nuclear RNA in the lymph nodes. We describe here three more ITK-deficient patients without overt EBV-related disease on presentation. EBV-DNA was undetectable in P2 at the ages of 16 and 17 yr, 7 yr after her first TB episode. Hence, to our knowledge, P2 is the first ITK-deficient patient characterized immunologically in the absence of either uncontrolled EBV infection or overt EBV-related disease. All three patients live in regions of endemic TB (P1 and P2 in Iran and P3 in Turkey), and their exposure to and infection with TB-causing virulent mycobacteria seems to have prompted the genetic diagnosis of ITK deficiency. In hindsight, recurrent and unexplained respiratory tract infections have been documented in seven reported ITK-deficient patients, including the 3-yr-old Turkish patient who died from respiratory insufficiency at the age of 3 yr (Çaǧdaş et al., 2017). The penetrance of ITK deficiency for classical EBV-driven hematological disorders is, thus, incomplete, at least by the mid-20s, and it is crucial to consider infections other than EBV-related diseases, including TB, when confronted with patients with inherited ITK deficiency. Conversely, ITK deficiency should be considered in patients presenting with severe TB.

Our extensive immunophenotyping analysis of P2 revealed the defects of leukocyte development caused by ITK deficiency without confounding due to uncontrolled EBV infection or disease. All the patients reported here had several immunophenotypes in common: (1) CD4^+^ αβ T lymphocytopenia, (2) an expansion of CD4^−^CD8^−^ DN αβ T lymphocytes, and (3) an expansion of Vδ1^+/−^Vδ2^−^ γδ T lymphocytes. Consistently, CD4^+^ αβ T lymphocytopenia and an expansion of DN αβ T and γδ T lymphocytes have been reported in Itk-deficient mice (Liao and Littman, 1995; Felices et al., 2009). We found that thymic and splenic γδ T lymphocytes had a CD73^+^PLZF^+^ phenotype in Itk-deficient mice, suggesting enhanced PLZF-driven differentiation into the γδ lineage in the thymus (Kreslavsky et al., 2009). By contrast, no expansion of the Vδ2^+^ T lymphocyte population was observed in the ITK-deficient patients, suggesting that the development of this human-specific subset is regulated differently. Furthermore, our extensive phenotyping analysis showed that the two expanded subsets—DN αβ T and Vδ2^−^ γδ T lymphocytes—had the same CD38^+^CD45RA^+^T-bet^+^EOMES^−^ phenotype, different from the “FAS-controlled” CD38^bright^CD45RA^+^T-bet^−^EOMES^+^ DN αβ T lymphocytes seen in patients with FAS deficiency or *STAT3* GOF mutations (Maccari et al., 2020). Although one ITK-deficient patient with a phenotype mimicking ALPS has been reported (Wallace et al., 2020), our analysis suggests that the expansion of the DN αβ T lymphocyte population is driven by different pathophysiological mechanisms in these two IEI. We found that splenic DN αβ T lymphocytes in Itk-deficient mice were skewed toward a mature and activated (CD5^hi^CD44^+^CD122^+^) phenotype analogous to IELPs (Hanke et al., 1994; McDonald et al., 2014). In normal mice, thymic IELPs become TCRαβ^+^CD4^−^CD8^−^ cells before egressing (Gangadharan et al., 2006) and acquiring T-bet expression in an IL-15–dependent manner in the gut (Klose et al., 2014; McDonald et al., 2014). ITK deficiency impairs IRF4 expression in humans and mice and the IRF4-dependent gut-homing properties of T lymphocytes in mice (Gomez-Rodriguez et al., 2016; Cho et al., 2020). We, therefore, speculate that ITK-deficient IEL-like DN αβ T lymphocytes cannot stably reside in the gut and that they instead re-enter the bloodstream. Consistent with this notion, activated (CD38^+^) and gut-homing (CD103^+^) IELs re-enter the bloodstream upon gluten challenge in patients with celiac disease (Han et al., 2013). Our analysis, therefore, suggests that ITK deficiency prompts commitment to the Vδ2^−^ γδ lineage in the thymus and prevents IEL-like DN αβ T lymphocytes from residing in the gut.

The three patients with inherited ITK deficiency reported here suffered from disseminated TB but not MSMD, despite BCG vaccination. Consistently, Itk-deficient mice display impaired bacterial clearance and aggravated lung disease after intranasal *M. tb* infection (Huang et al., 2020). ITK deficiency does not impair the development of IFN-γ–producing lymphocyte subsets, including T_H_1, T_H_1*, Vδ2^+^ γδ T, MAIT, iNKT, and NK lymphocytes, unlike genetic etiologies of MSMD, such as T-bet and RORγ deficiencies (Okada et al., 2015; Yang et al., 2020). However, functionally, IFN-γ production by ITK-deficient T lymphocytes was impaired upon TCR crosslinking, mitogen, or artificial immunological synapse formation. This impairment was phenocopied by pharmacological ITK inhibition and at least partially rescued by lentiviral complementation with WT *ITK*. Consistently, impaired IFN-γ production has been observed in (i) Itk-deficient murine CD4^+^ αβ T lymphocytes upon TCR crosslinking or after 3 d of culture under T_H_1-polarizing conditions (Schaeffer et al., 2001) and (ii) naive CD4^+^ αβ T lymphocytes from mice transgenic for an allele-sensitive mutant of Itk (Itkas) cultured in T_H_1-polarizing conditions in the presence of a specific inhibitor or Itkas (Kannan et al., 2015). However, enhanced IFN-γ production has been reported in (i) P/I-stimulated total αβ T lymphocytes from a patient with a homozygous LOF mutation of *ITK* (Eken et al., 2019), (ii) *ITK*-silenced, anti-CD3/CD28 antibody-stimulated human peripheral blood T lymphocytes (Kannan et al., 2013), and (iii) Itk-deficient murine CD4^+^ αβ T lymphocytes 10 d after pulmonary infection with *Schistosoma mansoni* (Schaeffer et al., 2001). Thus, ITK appears to control IFN-γ production in a context-dependent manner. By challenging ITK-deficient leukocytes (obtained long after the clinical remission of TB) with BCG, we demonstrated profoundly impaired IL-23-dependent IFN-γ production by MAIT and Vδ2^+^ γδ T lymphocytes, two well-established IFN-γ–producing lymphocyte subsets (Le Bourhis et al., 2010; Chen, 2016), and partially impaired IFN-γ production by CD4^+^, CD8^+^, and DN αβ T lymphocytes. Indeed, the impairment of IL-23 signaling confers a predisposition to TB in humans (Boisson-Dupuis et al., 2018; Ogishi et al., 2022). Overall, inherited ITK deficiency functionally impedes multiple IFN-γ–producing lymphocyte subsets, leading to insufficient protection against TB, but sufficient immunity to BCG and environmental mycobacteria.

## Materials and methods

### Case reports

The Iranian proband (P1) was a 13-yr-old boy born to a consanguineous family. He suffered from 3 mo of spiking fever, weight loss, night sweats, abdominal pain, and left-sided tonic-clonic seizure. Diagnostic imaging revealed one abdominal abscess and multiple mesenteric lymphadenopathies. Magnetic resonance imaging (MRI) of the head (T2-weighted) also revealed a high-intensity lesion in the right temporal lobe, suggesting cerebral mycobacterial infection. Histological examination revealed granulomatous lesions with central caseation and the presence of acid-fast bacilli (detected by Ziehl–Neelsen staining) in a surgical lymph node biopsy specimen. Bacterial culture of the abdominal abscess drainage material revealed virulent *M. bovis* (different from the BCG substrain, as proved by species-specific PCR). A tuberculin skin test (TST) was negative (0 mm induration), and sputum culture results were unremarkable. 9 mo of antimycobacterial therapy (isoniazid, rifampicin, ethambutol, and streptomycin) with concomitant subcutaneous recombinant human IFN-γ (Imukin, 50 μg/m^2^, three times per week) treatment for 6 mo led to complete remission of the disease, including cerebral lesion. This patient completed 4 yr of treatment and has been off treatment ever since. He did not suffer any adverse events (including BCG-itis) following the BCG vaccination performed 3 d after birth. Key features of his medical history included recurrent sinopulmonary infections at the age of 2 yr and generalized tonic-clonic seizures at the age of 3 yr (treated with carbamazepine for 1 yr). The patient had also had scattered common warts on the forehead and extremities since the age of 12. Cryotherapy was effective, but frequent recurrence of the warts was observed. HPV genotyping was not performed. At the age of 19 yr, this patient had high levels (>750 U/ml, saturated) of EBV VCA-IgG but no detectable VCA-IgM or EBNA antibodies. He also had high titers of EBV-DNA in the blood from the age of 19 onwards. He is now 28 yr old and healthy, despite the persistence of cutaneous warts, but with no recurrence of TB or any signs suggestive of EBV disease.

P1’s younger sister (P2) suffered from pulmonary TB at the age of 10 yr; this case has been published elsewhere (Majzoobi et al., 2019). Sputum culture revealed *M. tb*. A TST gave an induration of 5 mm (weakly positive). P2 recovered after 6 mo of antimycobacterial therapy (isoniazid, rifampicin, ethambutol, and pyrazinamide). She completed 2 yr of treatment and has remained off treatment ever since. However, at the age of 23 yr, she developed scrofuloderma (cutaneous TB) and retinal TB. She received standard four-drug antimycobacterial therapy for 6 mo. After 8 mo, she presented with hypertension and impaired renal function. Renal biopsy revealed granulomatous interstitial nephritis, but PCR was negative for *M. tb*. Combination therapy (isoniazid, rifampicin, ethambutol, and pyrazinamide) for 6 mo and recombinant human IFN-γ (Gammarec, 50 μg/m^2^, three times per week) treatment resulted in disease stabilization, with residual renal impairment. P2 was vaccinated with BCG 3 d after birth with no adverse events. She had had cutaneous warts on her extremities since the age of 15 yr and had suffered from genital warts since the age of 17 yr. These warts responded to cryotherapy, but they recurred frequently. HPV genotyping on a genital wart sample showed the presence of HPV type 6. At the age of 16 yr, P2 had high titers of EBV-VCA-IgG (>750 U/ml, saturated) but no detectable VCA-IgM or EBNA antibodies. Her blood tested negative for EBV-DNA at the ages of 16 and 17 yr, suggesting that she developed TB when her immunity was still capable of controlling EBV infection. However, she currently has high titers of EBV-DNA in her blood at the age of 25 yr. She is currently treated with recombinant human IFN-γ (Gammarec, 50 μg/m^2^, twice weekly) and remains clinically free from any EBV disease or TB recurrence.

The Turkish patient (P3) was a 4-yr-old boy born to a consanguineous family. He suffered from an intermittent chronic cough that persisted for a year. His familial history was unremarkable. Physical examination was also unremarkable, and the patient had no common warts. His chest x ray had a nodular appearance, and his thoracic CT scan showed the presence of miliary nodules spreading bilaterally in the lung and mediastinal lymphadenopathy. Abdominal ultrasound and cranial MRI scans showed no lesions. Serological tests for HIV were negative. A TST was negative, despite the patient having been vaccinated with BCG at the age of 2 mo. An IFN-γ release assay, Ziehl–Neelsen staining, and mycobacterial culture of a sputum specimen were also negative. Despite these negative test results, the patient was diagnosed with miliary TB on the basis of his clinical history and radiological findings. Treatment with a four-drug anti-TB regimen (rifampicin, isoniazid, pyrazinamide, and ethambutol) for 1 yr led to radiological and clinical improvement. However, the patient presented with fever and toothache at the age of 5 yr. Physical examination revealed a 2-cm palpable spleen, but the peripheral lymph nodes were not palpable. Chest x ray and CT scan revealed an enlarged mediastinal area. Positron emission tomography (PET)-CT showed FDG-avid lymphadenopathies in the mediastinum and spleen. EBV-DNA PCR was positive on blood (5.55 log IU/ml), whereas CMV, HHV-6, and HHV-7 were undetectable. No blasts were observed in a blood smear. Immunohistochemical examination of a bone marrow biopsy specimen showed CD15^+^CD30^+^CD45^−^ cells, and histological examination revealed the presence of Reed–Sternberg cells. EBV-DNA was detected in the bone marrow aspiration fluid (4.92 log IU/ml). The patient was diagnosed with stage IV Hodgkin’s lymphoma and was treated with a standard chemotherapy regimen plus rituximab. His fever abated and blood EBV-DNA became undetectable after two courses of chemotherapy.

### Human subjects

The patients and their parents, together with patients with biallelic LOF mutations of *TYK2* and *IL12RB1*, were recruited at the Necker Hospital for Sick Children. A patient with a biallelic LOF mutation of *FAS* was recruited at the National Institute of Allergy and Infectious Diseases. Healthy volunteers were recruited at The Rockefeller University. Written informed consent was obtained from all patients, family members, and healthy volunteers enrolled in this study. The study was approved by the institutional ethics committees of The Rockefeller University, Necker Hospital for Sick Children, and Sidra Medicine and performed in accordance with the requirements of these bodies. Experiments on samples from human subjects were conducted in the United States, France, and Qatar in accordance with local regulations and with the approval of the Institutional Review Board of the corresponding institution.

### Phage immunoprecipitation sequencing for microbial antigens (VirScan)

The reactivity of circulating antibodies against common pathogens in plasma samples from the two Iranian patients (P1 and P2) and healthy controls was profiled with an expanded version of the original VirScan library, as previously described (Béziat et al., 2021). Pooled human plasma for intravenous immunoglobulin therapy (Privigen CSL Behring AG) and IgG-depleted human serum (Molecular Innovations, Inc.) were used as additional controls.

### WES and variant filtering

WES was performed, and homozygosity rates were estimated from the patients’ genomic DNA, as previously described (Belkadi et al., 2016). Variant blacklisting was performed, as previously described (Maffucci et al., 2019). Minor allele frequencies (MAFs) in the general population, as reported in gnomAD database v2.1.1, and precomputed combined annotation–dependent depletion (CADD v1.3) scores (Kircher et al., 2014) were used for variant filtering. The MSC was calculated as previously described (Itan et al., 2016).

### Sanger sequencing

Genomic DNA was extracted from whole-blood samples and expanded T cell blasts (T-blasts) from the patients and their healthy family members. PCR products were sequenced with the BigDye Terminator Cycle Sequencing Kit (Applied Biosystems). Sequencing products were purified with Sephadex G-50 Superfine Resin (GE Healthcare). Sequences were determined with an ABI 3730 DNA Analyzer (Applied Biosystems). Sequencing spectrum data were analyzed with Geneious software (https://www.geneious.com).

### Cells

PBMCs were isolated from venous blood samples by Ficoll–Hypaque density gradient centrifugation (GE Healthcare). Cells were cryopreserved and stored at −150°C until use. Thawed PBMCs were allowed to rest temporarily in RPMI-1640 medium with GlutaMAX supplemented with 10% FBS (lymphocyte medium). T-blasts were generated by culturing PBMCs in ImmunoCult-XF T Cell Expansion Medium (Cat: 10981; StemCell Technologies) supplemented with ImmunoCult Human CD3/CD28/CD2 T-Cell Activator (Cat: 10970, 1:100; StemCell Technologies) and recombinant human IL-2 (rIL-2; Cat: 11147528001; Roche, 10 ng/ml). During continuous cultures, rIL-2 was added to the culture every 48 or 72 h. T-blasts were reactivated with the CD3/CD28/CD2 tetramer once every 10–14 d. Raji B lymphoma cells were cultured in a lymphocyte medium.

### Lentiviral transduction

A full-length coding sequence (CDS) of the WT human *ITK* gene was inserted into the pTRIP-CMV-Puro-2A vector (a gift from Nicolas Manel [Institut Curie, PSL Research University, INSERM U932, Paris, France]; plasmid #102611; Addgene; Gentili et al., 2015). The two mutants, c.496C>T (R166*) and c.1772T>C (M591T), were constructed by site-directed mutagenesis. Sequencing was performed to confirm the identity of the insert. For lentivirus production, HEK293T cells were dispensed in a 6-well plate at a density of 8 ×10^5^ cells/well, in 2 ml/well DMEM supplemented with 10% FBS. The next day, cells were transfected with pCMV-VSV-G (0.2 µg), pHXB2-env (0.2 µg; National Institutes of Health [NIH]–AIDS Reagent Program; #1069), psPAX2 (1 µg; a gift from Didier Trono [School of Life Sciences, Ecole Polytechnique Fédérale de Lausanne, Lausanne, Switzerland]; plasmid #12260; Addgene), and either pTRIP-CMV-Puro-2A or a vector encoding the protein of interest (1.6 µg) in Opti-MEM (Gibco, 300 µl) containing Lipofectamine 2000 (Invitrogen, 10 µl) and PLUS Reagent (Invitrogen, 3 µl), according to the manufacturer’s protocol. After 6 h of transfection, the medium was replaced with 3 ml DMEM supplemented with 10% FBS and 150 µl 20% BSA/PBS (final concentration: 1% BSA), and the cells were incubated for a further 60 h for the production of lentiviral particles. The viral supernatant was harvested, centrifuged at 500 *g* for 10 min at 4°C to remove debris, and concentrated with the Lenti-X Concentrator (Cat: 631232; Takara Bio). Protamine sulfate (Sigma-Aldrich, 10 µg/ml) was added to the resuspended viral stock, which was then used immediately or stored at −80°C until use.

HEK293T cells were dispensed in a 6-well plate at a density of 2 × 10^5^ cells/well in 2 ml of DMEM with 10% FBS. The next day, the cells were infected with the concentrated lentiviruses and incubated for 48 h at 37°C. The medium was then replaced and puromycin was added (5 µg/ml).

T-blasts (restimulated with anti-CD3/CD28/CD2 tetramer 2 d before transduction) were dispensed into a U-bottomed 96-well plate at a density of 2 × 10^5^ cells/well in 100 µl of ImmunoCult-XF T Cell Expansion Medium. Viral supernatant was added (100 µl per well) and the cells were spinoculated for 2 h at 1,200 *g* at 25°C. The cells were further incubated for 48 h at 37°C. The medium was then replaced with ImmunoCult-XF T Cell Expansion Medium supplemented with rIL-2. Puromycin was added 96 h after spinoculation (2 µg/ml).

### Immunoblotting analysis of ITK expression and signaling

Immunoblotting was performed with HEK293T cells, as previously described (Ogishi et al., 2021). Briefly, total protein was extracted from HEK293T cells stably transduced with an EV, WT ITK, R166* ITK, or M591 ITK with or without transient transfection with the WT *PLCG1*-DDK plasmid or EV. The *PLCG1* plasmid, purchased from OriGene, harbors the full-length CDS of WT human *PLCG1* with a C-terminal DYKDDDDK-tag. PhosStop (Cat: 4906845001; Sigma-Aldrich) was included for PLCγ1 phosphorylation analysis. SDS-PAGE was performed in 12.5 and 7.5% polyacrylamide gels for ITK and PLCγ1, respectively. The resulting protein bands were transferred onto a polyvinylidene difluoride membrane. The membrane was probed with the following primary antibodies: anti-ITK mAb for the N-terminal epitope (Cat: ab32039; Abcam, Clone: Y401, 1:1,000, 4 °C overnight), anti-ITK mAb for the C-terminal epitope (Cat: 77215; Cell Signaling Technology, Clone: E4X7M, 1:1,000, 4 °C overnight), anti-phospho-PLCγ1 (Y783) rabbit polyclonal antibody (Cat: 2821, 1:1,000; Cell Signaling Technology, 4 °C overnight), HRP-conjugated anti-DDK mAb (Cat: 637311, Clone: L5, 1:2,000; BioLegend, room temperature for 1 h), and HRP-conjugated anti-vinculin mAb (Cat: sc-73614, Clone: 7F9, 1:1,000; Santa Cruz, 4 °C overnight).

Total T-blasts cultured for 10–14 d were analyzed by immunoblotting. ITK expression was analyzed with the following antibodies: anti-ITK mAb for the N-terminal epitope (Cat: 2380, Clone: 2F12, 1:1,000; Cell Signaling Technology, 90 min at room temperature), anti-ITK rabbit polyclonal antibody for the C-terminal epitope (Cat: ab3213; Abcam), and anti-KU mAb (Cat: 4103, Clone: D35, 1:1,000; Cell Signaling Technology). For PLCγ1 analysis, T-blasts were stimulated with the anti-CD3 mAb (Clone: OKT3, 1 mg/ml) followed by rabbit anti-mouse IgG (2 mg/ml) for the times indicated. Cells (4 × 10^6^ cells for each set of conditions) were then lysed in 1% NP40, 50 mM Tris pH 8, 150 mM NaCl, 20 mM EDTA, 1 mM Na_3_VO_4_, 1 mM NaF, and complete protease inhibitor cocktail (Roche). Protein concentrations were determined in a BCA assay (Bio-Rad). We then subjected 80 mg of protein to SDS–PAGE and transferred the resulting bands onto PVDF membranes (Millipore). The membranes were blocked with milk or BSA and then incubated with antibodies. The following antibodies were used for immunoblotting: anti-PLCγ1 polyclonal antibody (Cat: 2822, 1:1,000; Cell Signaling Technology, room temperature for 1 h), anti-phospho-PLCγ1 (Y783; Cat: 2821, 1:1,000; Cell Signaling Technology, room temperature for 1 h), anti-phosphorylated tyrosine (Cat: 96215, Clone: 4G10, 1:1,000; Cell Signaling Technology, room temperature for 1 h), anti-KU70 (Cat: 4103, 1:1,000; Cell Signaling Technology, room temperature for 1 h), anti-phospho-ZAP70 (Y319; Cat: 2701, 1:1,000; Cell Signaling Technology, 4 °C overnight), anti-ZAP70 (Cat: 3165, Clone: D1C10E, 1:1,000; Cell Signaling Technology, 4 °C overnight), and anti-α-tubulin (Cat: sc-5286, Clone: B-7, 1:200; Santa Cruz Biotechnology, 4 °C overnight) antibodies. The membranes were then washed and incubated with anti-mouse or anti-rabbit HRP-conjugated secondary antibodies. The Pierce ECL Western Blotting Substrate or SuperSignal West Femto Maximum Sensitivity Substrate was used for detection.

### Calcium influx assay

Calcium influx into total T lymphocytes from freshly thawed PBMCs was analyzed by real-time flow cytometry, as previously described (Hauck et al., 2012). Briefly, cells were loaded with indo-1 acetoxymethyl ester (Molecular Probes, 5 μM) in the presence of 2.5 mM probenecid (PowerLoad; Molecular Probes), washed, surface-stained for CD4 and CD8, washed, stimulated with anti-CD3 mAb (Clone: OKT3, 1 μg/ml) followed by F(ab′)_2_ rabbit–anti-mouse IgG (Jackson Immunoresearch, 10 μg/ml), and then further stimulated with ionomycin (Sigma-Aldrich, 1 μM). Cells were analyzed with a FACSAria flow cytometer (BD Biosciences). Intracellular Ca^2+^ levels were quantified by calculating the normalized ratio of fluorescence intensity (Ca^2+^-bound to Ca^2+^-free Indo-1) and plotted as a function of time.

### Flow cytometric immunophenotyping of primary leukocytes

Immunophenotyping analysis was performed as described previously (Ogishi et al., 2022). Briefly, freshly thawed PBMCs (1.2 × 10^6^ cells) were simultaneously stained with LIVE/DEAD Fixable Blue dye (Cat: L23105, 1:800; Thermo Fisher Scientific in PBS) and blocked by incubation with FcR blocking reagent (Miltenyi Biotec, 1:25) on ice for 15 min. After washing, cells were surface-stained with the following reagents on ice for 30 min: Brilliant Stain Buffer Plus (Cat: 566385; BD Biosciences, 1:5), anti-γδTCR-BUV661 (Cat: 750019; BD Biosciences, Clone: 11F2, 1:50), anti-CXCR3-BV750 (Cat: 746895, Clone: 1C6, 1:20; BD Biosciences), and anti-CCR4-BUV615 (Cat: 613000, Clone: 1G1, 1:20; BD Biosciences) antibodies. Cells were then washed and surface-stained with the following reagents on ice for 30 min: 5-OP-RU-loaded MR1 tetramer-BV421 (NIH Tetramer Core Facility, 1:100), anti-CD141-BB515 (Cat: 565084, Clone: 1A4, 1:40; BD Biosciences), anti-CD57-FITC (Cat: 347393, Clone: HNK-1, 3:250; BD Biosciences), anti-Vδ2-PerCP (Cat: 331410, Clone: B6, 3:500; BioLegend), anti-Vα7.2-PerCP-Cy5.5 (Cat: 351710, Clone: 3C10, 1:40; BioLegend), anti-Vδ1-PerCP-Vio700 (Cat: 130-120-441, Clone: REA173, 1:100; Miltenyi Biotec), anti-CD14-Spark Blue 550 (Cat: 367148, Clone: 63D3, 1:40; BioLegend), anti-CD1c-Alexa Fluor 647 (Cat: 331510, Clone: L161, 1:50; BioLegend), anti-CD66b-APC (Cat: 305118, Clone: G10F5, 1:50; BioLegend), anti-CD38-APC-Fire 810 (Cat: 356644, Clone: HB-7, 3:100; BioLegend), anti-CD27-APC H7 (Cat: 560222, Clone: M-T271, 1:50; BD Biosciences), anti-CD127-APC-R700 (Cat: 565185, Clone: HIL-7R-M21, 1:50; BD Biosciences), anti-CD19 Spark NIR 685 (Cat: 302270, Clone: HIB19, 3:250; BioLegend), anti-CD45RA-BUV395 (Cat: 740315, Clone: 5H9, 3:250; BD Biosciences), anti-CD16-BUV496 (Cat: 612944, Clone: 3G8, 3:500; BD Biosciences), anti-CD11b-BUV563 (Cat: 741357, Clone: ICRF44, 1:100; BD Biosciences), anti-CD56-BUV737 (Cat: 612767, Clone: NCAM16.2, 3:250; BD Biosciences), anti-CD4-cFluor 568 (Cytek, Clone: SK3, 3:250), anti-CD8-BUV805 (Cat: 612889, Clone: SK1, 3:250; BD Biosciences), anti-CD11c-BV480 (Cat: 566135, Clone: B-ly6, 1:40; BD Biosciences), anti-CD45-BV510 (Cat: 563204, Clone: HI30, 3:250; BD Biosciences), anti-CD33-BV570 (Cat: 303417, Clone: WM53, 3:250; BioLegend), anti-iNKT-BV605 (Cat: 743999, Clone: 6B11, 1:25; BD Biosciences), anti-CD161-BV650 (Cat: 563864, Clone: DX12, 1:25; BD Biosciences), anti-CCR6-BV711 (Cat: 353436, Clone: G034E3, 3:250; BioLegend), anti-CCR7-BV785 (Cat: 353230, Clone: G043H7, 1:40; BioLegend), anti-CD3-Pacific Blue (Cat: 344824, Clone: SK7, 3:250; BioLegend), anti-CD20-Pacific Orange (Cat: MHCD2030, Clone: HI47, 1:50; Invitrogen), anti-CD123-Super Bright 436 (Cat: 62-1,239-42, Clone: 6H6, 1:40; Invitrogen), anti-Vβ11-PE (Cat: 130-123-561, Clone: REA559, 3:500; Miltenyi Biotec), anti-CD24-PE-Alexa Fluor 610 (Cat: MHCD2422, Clone: SN3, 1:25; Invitrogen), anti-CD25-PE-Alexa Fluor 700 (Cat: MHCD2524, Clone: 3G10, 1:25; Invitrogen), anti-CRTH2-Biotin (Cat: 13-2949-82, Clone: BM16, 1:50; Invitrogen), anti-CD209-PE-Cy7 (Cat: 330114, Clone: 9E9A8, 1:25; BioLegend), anti-CD117-PE-Dazzle 594 (Cat: 313226, Clone: 104D2, 3:250; BioLegend), and anti-HLA-DR-PE-Fire 810 (Clone: L243, 1:50; BioLegend) antibodies. After washing, cells were further incubated with streptavidin-PE-Cy5 (Cat: 405205, 1:3,000; BioLegend) on ice for 30 min. Cells were then washed, fixed with 1% paraformaldehyde/PBS, washed again, and acquired with an Aurora cytometer (Cytek). Subsets were manually gated with FlowJo v10 (FlowJo, LLC) and further analyzed in R. The cellular composition was visualized with the data downsampled to 50,000 cells per sample through Uniform Manifold Approximation and Projection (UMAP) based on the expression levels of the following markers: CD3, CD4, CD8, CD11c, CD14, CD16, CD19, CD20, CD56, CD117, CD123, CD127, CD161, CRTH2, HLA-DR, γδTCR, Vδ1, Vδ2, iNKT, and MR1.

### Immunophenotyping of CD4^−^CD8^−^ DN αβ T lymphocytes

Freshly thawed PBMCs (1.0 × 10^6^ cells) were stained with Zombie NIR Fixable Viability dye (Cat: 423105, 1:1,000; BioLegend in PBS) at 4°C in the dark for 15 min. The cells were washed and surface-stained with the following reagents at 4°C for 45 min: FcR blocking reagent (Miltenyi Biotec, 1:50), 5-OP-RU-loaded MR1 tetramer-BV421 (NIH Tetramer Core Facility, 1:200), anti-FAS-BUV395 (Cat: 740306, Clone: DX2, 1:100; BD Biosciences), anti-CD4-BUV563 (Cat: 612912, Clone: SK3, 1:200; BD Biosciences), anti-FASLG-BUV615 (Cat: 752306, Clone: NOK-1, 1:100; BD Biosciences), anti-γδTCR-BUV661 (Cat: 750019, Clone: 11F2, 1:50; BD Biosciences), anti-CD8-BUV737 (Cat: 612755, Clone: SK1, 1:450; BD Biosciences), anti-Vδ2-BUV805 (Cat: 748580, Clone: B6, 1:450; BD Biosciences), anti-iNKT-BV480 (Cat: 746788, Clone: 6B11, 1:50; BD Biosciences), anti-CD34-BV510 (Cat: 343527, Clone: 581, 1:100; BioLegend), anti-CD1a-BV711 (Cat: 300139, Clone: HI149, 1:100; BioLegend), anti-CD38-BV785 (Cat: 303530, Clone: HIT2, 1:100; BioLegend), anti-CD66b-FITC (Cat: 305104, Clone: G10F5, 1:100; BioLegend), anti-CD45-Alexa Fluor 532 (Cat: 58-0459-41, Clone: HI30, 1:100; eBioscience), anti-Vα7.2-PerCP-Cy5.5 (Cat: 351710, Clone: 3C10, 1:40; BioLegend), anti-PD-1-BB700 (Cat: 566461, Clone: EH12.1, 1:100), anti-Vδ1-PerCP-Vio 700 (Miltenyi Biotec, Cat: 130-120-441, Clone: REA173, 1:100; BD Biosciences), anti-CD45RA-PE-Cy5 (Cat: 552888, Clone: 5H9, 1:100; BD Biosciences), anti-CCR7-Alexa Fluor 647 (Cat: 353218, Clone: G043H7, 1:50; BioLegend), anti-CD20-Alexa Fluor 700 (Cat: 560631, Clone: 2H7, 1:100; BD Biosciences) and anti-CD56-Alexa Fluor 700 (Cat: 561902, Clone: B159, 1:50; BD Biosciences), anti-Vβ11-APC-Vio 770 (Cat: 130-127-292, Clone: REA559, 1:50; Miltenyi Biotec), and anti-HLA-DR-APC-Fire 810 (Cat: 307674, Clone: L243, 1:100; BioLegend) mAbs. The cells were washed, fixed at room temperature in the dark for 45 min, and permeabilized by incubation at room temperature in the dark for 5 min with the True-Nuclear Transcription Factor Buffer Set (Cat: 424401; BioLegend). The cells were then intracellularly stained by incubation with the following reagents in permeabilization buffer at 4°C overnight: FcR Blocking Reagent (Miltenyi Biotec, 1:50), anti-CD3-V450 (Cat: 560366, Clone: UCHT1, 1:200; BD Biosciences), anti-T-bet-PE-Cy7 (Cat: 644824, Clone: 4B10, 1:1,000; BioLegend), anti-RORγT-PE (Cat: 563081, Clone: Q21-559, 1:50; BD Biosciences), and anti-EOMES-PE-Cy5.5 (Cat: 35-4877-41, Clone: WD1928, 1:100; eBioscience) mAbs. The next day, the cells were washed three times with FACS buffer and acquired with an Aurora cytometer (Cytek). Subsets were manually gated with Flowjo and further analyzed in R. For FlowSOM analysis, CD4^−^CD8^−^ DN αβ T lymphocytes were manually gated with FlowJo and imported into R. The expression levels of the following markers were used for FlowSOM-guided unsupervised clustering: CD4, CD8, CCR7, CD45RA, CD1a, CD34, CD38, FAS, FASLG, HLA-DR, PD-1, T-bet, RORγT, and EOMES. Clusters were manually identified based on their marker expression pattern. The cellular composition was visualized with the data downsampled to 2,500 cells per sample with UMAP based on the expression levels of the same markers.

### Immunophenotyping analysis in Itk-deficient mice

Itk knockout (Liao and Littman, 1995) and WT mice were backcrossed for at least 12 generations onto the C57BL/6 background. All mice used were 6- to 8-wk-old. The animals were euthanized, and their thymus and spleen were dissected. Single-cell suspensions were prepared by homogenizing tissues in complete RPMI media by passage through filters with 70 μm pores. After dead cell staining with a Live/Dead Aqua dye (Invitrogen), surface staining was performed in FACS buffer (PBS supplemented with 1% FBS and 1 mM EDTA) for 30 min at 4°C with the following reagents: anti-CD16/CD32 (Cat: BE0307, Clone: 2.4G2, 1:200; BioXCell), anti-CD4-BUV737 (Cat: 612761, Clone: GK1.5, 1:200; BD Biosciences), anti-CD8α-BUV395 (Cat: 563786, Clone: 53-6.7, 1:200; BD Biosciences), anti-CD44-PE-Cy7 (Cat: 103030, Clone: IM7, 1:200; BioLegend), anti-CD25-Alexa Fluor 700 (Cat: 56-0251-82, Clone: PC61.5, 1:200; Invitrogen), PBS-57-loaded CD1d tetramer-Alexa Fluor 488 (NIH Core Tetramer Facility, 1:200), 5-OP-RU-loaded MR1 tetramer-BV421 (NIH Core Tetramer Facility, 1:100), anti-PD-1-BV785 (Cat: 135225, Clone: 29F.1A12, 1:200; BioLegend), anti-TCRβ-BV650 (Cat: 742483, Clone: H57-597, 1:200; BD Biosciences), anti-CD5-APC-eFluor 780 (Cat: 47-0051-82, Clone: 53-7.3, 1:200; Invitrogen), anti-CD122-biotin (Cat: 13-1,222-85, Clone: TM-b1, 1:200; Invitrogen), streptavidin-BV711 (Cat: 405241, 1:200; BioLegend), anti-γδTCR-BV711 (Cat: 563994, Clone: GL3, 1:400; BD Biosciences), anti-CD3-PE-Cy7 (Cat: 25-0031-82, Clone: 145-2C11, 1:200; Invitrogen), anti-CD24-biotin (Cat: 553260, Clone: M1/69, 1:400; BD Biosciences), streptavidin-BV421 (Cat: 405226, 1:200; BioLegend), anti-CD73-PerCP-Cy5.5 (Cat: 127213, Clone: TY/11.8, 1:200; BioLegend), and anti-CD27-APC-eFluor 780 (Cat: 47-0271-80, Clone: LG.7F9, 1:200; Invitrogen). Cells were fixed and permeabilized with the Foxp3/Transcription Factor staining kit (Cat: 00-5523-00; eBioscience). Intracellular staining was performed for 1 h at 4°C with the following reagents: anti-CD16/CD32 (BioXCell, 1:200), anti-T-bet-BV605 (Cat: 644817, Clone: 4B10, 1:100; BioLegend), anti-RORγT-PE-CF594 (Cat: 562684, Clone: Q31-378, 1:200; BD Biosciences), anti-PLZF-Alexa Fluor 488 (Cat: 53-9320-82, Clone: Mags.21F7, 1:200; Invitrogen), and anti-EOMES-PerCP-eFluor 710 (Cat: 46-4875-82, Clone: Dan11mag, 1:400; Invitrogen) antibodies. Stained samples were acquired on a BD LSRFortessa and analyzed with FlowJo software. Animals were reared, and the experiments were performed in accordance with protocol G98.3 approved by the National Human Genome Research Institute’s Animal Use and Care Committee, National Institutes of Health.

### Preparation of antibody-conjugated beads

Antibody-conjugated beads were prepared as previously described (Patsoukis et al., 2012; Ogishi et al., 2021). Briefly, Dynabeads M-450 Epoxy beads (Cat: 14011; Invitrogen, 8 × 10^7^ beads per reaction) were covalently conjugated with Ultra-LEAF-purified anti-CD3 antibody (Cat: 317236, Clone: OKT3; BioLegend, 3.2 μg) and Ultra-LEAF-purified anti-CD28 antibody (Cat: 302934, Clone: CD28.2; BioLegend, 4 μg). The beads were washed twice with 0.1% BSA in PBS supplemented with 2 mM EDTA and stored at 4°C in the dark for subsequent experiments.

### Analysis of cytokine production by primary leukocytes

Freshly thawed PBMCs (3.0 × 10^5^ cells per well) were dispensed into a U-bottomed 96-well plate in 100 µl of RPMI with 10% non-heat-inactivated human serum (Cat: H4522-20Ml; Sigma-Aldrich) and left unstimulated or they were stimulated by incubation with the following reagents for 18 h: Dynabeads Human T-Activator CD3/CD28 beads (Cat: 11131D; Gibco, bead:cell = 1:1), ImmunoCult Human CD3/CD28/CD2 T-Cell Activator (Cat: 10970; StemCell Technologies, final dilution 1:100), PHA-M (Cat: 10576015; Gibco, final dilution 1:100), and Cell Stimulation Cocktail (Cat: 00-4970-93; eBioscience, final 1:1,000). For blinatumomab-mediated T cell activation, freshly thawed PBMCs (1.0 × 10^5^ cells per well) were dispensed into a U-bottomed 96-well plate in 100 µl of RPMI with 10% of non-heat-inactivated human serum and rhIL-2 (1:500). They were left unstimulated or were stimulated with blinatumomab (BPS Biosciences, Cat: 100441-2, final concentration 10 ng/ml) for 3 or 5 d. Supernatants were harvested and analyzed with the LEGENDplex HU Th Cytokine Panel V02 kit (Cat: 741027; BioLegend). Beads were acquired with an Attune NxT Flow Cytometer with the CytKick MAX Autosampler (Invitrogen).

### Analysis of cytokine production by T-blasts

T-blasts were dispensed into a U-bottomed 96-well plate at a density of 1 × 10^5^ cells/well in 100 μl/well lymphocyte medium without rIL-2 supplementation. Cells were incubated with unconjugated Dynabeads or Dynabeads conjugated with anti-CD3 and anti-CD28 mAbs for 2 h at 37°C. We then added anti-CD107a-Alexa Fluor 647 antibody (BioLegend, Cat: 328612, Clone: H4A3, 1:200) and GolgiStop (Cat: 554724, 1:1,600; BD Biosciences) and incubated for an additional 5 h at 37°C. Cells were then harvested, stained with Live/Dead Fixable Aqua dye (Cat: L34957; Invitrogen, 1:1,000 in PBS) for 15 min at 4°C in the dark, and surface-stained by incubation with anti-CD3-BV421 (Cat: 300434, Clone: UCHT1, 1:200; BioLegend), anti-CD4-redFluor 700 (Cat: 80-0048, Clone: OKT4, 3:200; Tonbo Biosciences), and anti-CD8-FITC (Cat: 301060, Clone: RPA-T8, 1:200; BioLegend) mAbs at 4°C in the dark for 30 min. The cells were washed with FACS buffer, fixed by incubation at room temperature for 45 min in the dark, and permeabilized by incubation at room temperature in the dark for 5 min with the True-Nuclear Transcription Factor Buffer Set (BioLegend). Cells were then subjected to intracellular staining overnight at 4°C in the dark with the following panel of antibodies in the permeabilization buffer: FcR Blocking Reagent (Miltenyi Biotec, 1:50), anti-IFN-γ-PE-Dazzle 594 (Cat: 502546, Clone: 4S.B3, 1:100; BioLegend), anti-TNF-BV711 (Cat: 502940, Clone: MAb11, 1:100; BioLegend), anti-IL-17A-APC-Cy7 (Cat: 512320, Clone: BL168, 1:100; BioLegend), anti-T-bet-PE-Cy7 (Cat: 644824, Clone: 4B10, 1:500; BioLegend), and anti-RORγT-PerCP/eFluor 710 (Cat: 46-6988-80, Clone: AFKJS-9, 1:500; eBioscience) antibodies. The next day, the cells were washed three times with FACS buffer and acquired with a BD LSR II Flow Cytometer (BD Biosciences).

For pharmacological ITK inhibition, CD4^+^ T-blasts from healthy donors were magnetically sorted with the CD4^+^ T-Cell Isolation Kit (Cat: 130-096-533; Miltenyi Biotec). Cells (2 × 10^5^ cells/well) were incubated in 50 μl/well lymphocyte medium supplemented with dimethyl sulfoxide (DMSO) with or without BMS509744 (Cat: 5009; Tocris, Batch: 2B/247519) for 1 h. The stock solution (10 mM) was added at a dilution of 1:1,000 (vol/vol). The stimuli were then added in 50 μl lymphocyte medium, giving a final BMS509744 concentration of 5 μM. Cells were then stimulated by incubation with the following reagents for 6 h: unconjugated Dynabeads or Dynabeads conjugated with in-house anti-CD3 and anti-CD28 mAbs (bead:cell ratio = 1:1), ImmunoCult Human CD3/CD28/CD2 T-Cell Activator (StemCell Technologies, final 1:100), PHA-M (Gibco, final 1:100), and Cell Stimulation Cocktail (eBioscience, final 1:1,000). The levels of secreted cytokines were determined with the LEGENDplex HU Th Cytokine Panel V02 kit (BioLegend).

### Whole-blood BCG stimulation assay

Venous blood samples were drawn from one local control, one travel control, the two Iranian patients, and their father. Samples were collected in heparin-containing tubes and processed as previously described (Boisson-Dupuis et al., 2018). Briefly, blood samples were diluted 1:2 in RPMI-1640 medium (Gibco) and supplemented with penicillin (100 U/ml) and streptomycin (Gibco, 100 μg/ml). The samples were then dispensed into a 48-well plate (1 ml/well) and were either left non-stimulated or were stimulated with live *M. bovis* BCG Pasteur substrain at a multiplicity of infection of 20 BCG cells per leukocyte or with BCG plus recombinant human IL-12 (Cat: 219-IL; R&D Systems, 20 ng/ml). As a positive control, cells in separate wells were stimulated with phorbol 12-myristate 13-acetate (PMA, Cat: P8139; Sigma-Aldrich, 40 ng/ml) and ionomycin (Cat: I9657; Sigma-Aldrich, 10 μM). After 48 h of stimulation, the levels of secreted cytokines in the supernatant were determined with the human IFN-γ ELISA kit (Cat: 50-173-27; Invitrogen) and the human IL-12p40 ELISA kit (Cat: KAC1561; Invitrogen), according to the manufacturer’s instructions.

### PBMC BCG stimulation assay

Freshly thawed PBMCs were dispensed into a U-bottomed 96-well plate at a density of 2 × 10^5^ cells/well in 200 μl/well lymphocyte medium. Cells were incubated in the presence or absence of live BCG at a multiplicity of infection of 1, with or without recombinant human IL-12 (R&D Systems, 500 pg/ml) or recombinant human IL-23 (Cat: 1290-IL; R&D Systems, 10 ng/ml). After 40 h of stimulation, GolgiPlug (Cat: 555029; BD Biosciences, 1:1,000) was added to each well to inhibit cytokine secretion. After another 8 h of incubation, supernatants were collected and stored at −20°C for the determination of cytokine levels, and the cells were harvested by centrifugation for flow cytometry analysis. The cells were stimulated simultaneously with PMA (Sigma-Aldrich, 25 ng/ml) and ionomycin (Sigma-Aldrich, 500 nM) for 8 h without GolgiPlug (for the determination of secreted cytokines) or for 1 h without GolgiPlug followed by 7 h with GolgiPlug (for intracellular cytokine staining) as a positive control. The levels of secreted cytokines in the supernatants were determined with the LEGENDplex Human Inflammation Panel 1 13-plex kit (Cat: 740809; BioLegend). Data were analyzed in R. For flow cytometric analysis, the cells were first stained with the Zombie NIR dye (BioLegend, 1:2,000 in PBS) at room temperature for 15 min and were then incubated on ice for 30 min with a surface staining panel containing FcR blocking reagent (Miltenyi Biotec, 1:50), 5-OP-RU-loaded MR1 tetramer-BV421 (NIH Tetramer Core Facility, 1:200), anti-CD3-V450 (Cat: 560365, Clone: UCHT1, 1:450; BD Biosciences), anti-CD4-BUV563 (Cat: 612912, Clone: SK3, 1:450; BD Biosciences), anti-CD8-BUV737 (Cat: 612754, Clone: SK1, 1:450; BD Biosciences), anti-CD20-BV785 (Cat: 302356, Clone: 2H7, 1:150; BioLegend), anti-CD56-BV605 (Cat: 362538, Clone: 5.1H11, 1:50; BioLegend), anti-γδTCR-Alexa Fluor 647 (Cat: 331214, Clone: B1, 1:50; BioLegend), anti-Vδ1-FITC (Cat: 130-118-362, Clone: REA173, 1:150; Miltenyi Biotec), anti-Vδ2-APC-Fire 750 (Cat: 331420, Clone: B6, 1:1,350; BioLegend), anti-Vα7.2-Alexa Fluor 700 (Cat: 351728, Clone: 3C10, 1:50; BioLegend), anti-iNKT-BV480 (Cat: 746788, Clone: 6B11, 1:50; BD Biosciences), and anti-Vβ11-APC (Cat: 130-125-529, Clone: REA559, 1:150; Miltenyi Biotec) antibodies. Cells were fixed by incubation with 2% paraformaldehyde/PBS on ice for 15 min and were then permeabilized/stained by incubation overnight at −20°C in the perm buffer from the True-Nuclear Transcription Factor Buffer Set (BioLegend) with an intracellular cytokine panel containing FcR blocking reagent (Miltenyi Biotec, 1:50), anti-IFN-γ-BV711 (Cat: 502540, Clone: 4S.B3, 1:450; BioLegend), anti-TNF-BV510 (Cat: 502950, Clone: MAb11, 1:150; BioLegend), anti-IL-17A-PerCP-Cy5.5 (Cat: 512314, Clone: BL168, 1:1,350; BioLegend), anti-T-bet-PE-Cy7 (Cat: 644823, Clone: 4B10, 1:1,350; BioLegend), and anti-RORγT-PE (Cat: 563081, Clone: Q21-559, 1:50; BD Biosciences) antibodies. Cells were acquired with an Aurora cytometer (Cytek). Data were manually gated with FlowJo and then imported into R for further analysis. The cellular composition was visualized with UMAP based on the expression levels of CD3, CD4, CD8, CD20, CD56, γδTCR, Vδ1, Vδ2, Vα7.2, MR1, T-bet, and RORγT, with the data downsampled to 10,000 cells per sample.

### Analysis of IL-23 signaling

Freshly thawed PBMCs from one to three healthy donors were stained with Live/Dead Aqua dye (Invitrogen) and then with a surface staining panel containing anti-CD3-BV786 (Cat: 563800, Clone: SK7; BD Biosciences), anti-CD4-APC (Cat: 555349, Clone: RPA-T4; BD Biosciences), anti-CD8-BV650 (Cat: 563821, Clone: RPA-T8; BD Biosciences), anti-CD56-PE-CF594 (Cat: 562289, Clone: NCAM-1; BD Biosciences), anti-CD161-BV711 (Cat: 563865, Clone: DX12; BD Biosciences), anti-Vδ2-PE (Cat: 331408, Clone: B6; BioLegend), and anti-Vα7.2-PerCP-Vio 700 (Cat: 130-124-075, Clone: REA179; Miltenyi Biotec) antibodies. MAIT cells and Vδ2^+^ γδ T cells were sorted on a FACS Aria II SORP (BD Biosciences) and cultured for 2 wk with allogeneic PBMCs irradiated at 35 Gy (T cell:feeder cell ratio of 1:10) in ImmunoCult-XF T Cell Expansion Medium (StemCell Technologies) supplemented with 8% FBS, 10 ng/ml recombinant human IL-2 (Gibco), and 100 U/ml penicillin/streptomycin (Thermo Fisher Scientific). Phosphorylated STAT3 levels were determined in 5 × 10^5^ expanded serum-starved T cells incubated with or without DMSO alone or DMSO containing BMS509744 (Cat: HY-11092; CliniSciences, 5 μM) for 1 h. Cells were then incubated for 30 min with recombinant human IL-23 (Cat: 1290-IL; R&D Systems, 1 or 10 ng/ml) or IFNα-2b (Merck, Intron A, 10^4^ IU/ml), stained with Live/Dead Aqua dye (Invitrogen) for 5 min at 4°C, fixed with Fix buffer I (1:1 volume, BD Biosciences), permeabilized in Perm buffer III (BD Biosciences), and stained with anti-STAT3 (pY705)-PE antibody (Cat: 612569, Clone: 4/P-STAT3, 1:25; BD Biosciences) at 4°C overnight. Cells were acquired with a Gallios flow cytometer (Beckman Coulter). For the assessment of cytokine secretion, 2 × 10^5^ expanded T cells resuspended in RPMI-1640 medium supplemented with 10% human serum (Sigma-Aldrich) were stimulated with recombinant IL-23 (100 ng/ml) with or without IL-1β (Cat: 201-LB; R&D Systems, 2.5 ng/ml) for 48 h or with PMA (Sigma-Aldrich, 8 ng/ml) and ionomycin (Sigma-Aldrich, 10 μM) for 12 h in the presence or absence of DMSO alone or DMSO containing BMS509744 (Cat: HY-11092; CliniSciences, 5 μM). Secreted cytokine levels were determined with the LEGENDplex Human Inflammation Panel 1 (Cat: 740809; BioLegend).

### Raji cell killing assay

Freshly thawed PBMCs from two or three healthy donors were stained for 1 h at 4°C in the dark with a surface-staining panel containing FcR blocking reagent (Miltenyi Biotec, 1:50), anti-CD3-APC (Tonbo Biosciences, Cat: 20-0038-T100, Clone: UCHT1, 1:100), anti-γδTCR-BV421 (Cat: 744870, Clone: 11F2, 1:100; BD Biosciences), anti-Vδ1-PerCP-Vio 700 (Cat: 130-120-441, Clone: REA173, 1:100; Miltenyi Biotec), anti-Vδ2-APC-Fire 750 (Cat: 331420, Clone: B6, 1:100; BioLegend), anti-CD161-Alexa Fluor 488 (Cat: 339923, Clone: HP-3G10, 1:100; BioLegend), anti-Vα7.2-BV711 (Cat: 351731, Clone: 3C10, 1:100; BioLegend), anti-iNKT-BV480 (Cat: 746788, Clone: 6B11, 1:100; BD Biosciences), and anti-Vβ11-PE-Vio 770 (Cat: 130-126-319, Clone: REA559, 1:100; Miltenyi Biotec) antibodies. Dead cells were then stained with 7-aminoactinomycin D (7-AAD; Tonbo Biosciences, Cat: 13-6993-T500, 1:200). Vδ1^+^ γδ T, Vδ2^+^ γδ T, MAIT, and iNKT cells were sorted with a FACSAria cell sorter (BD Biosciences). Sorted innate-like adaptive T lymphocytes were expanded by incubation for ∼2 wk with the ImmunoCult reagents and IL-2 plus Raji cells irradiated at 90 Gy as the feeder cells.

After expansion, T cells were incubated with DMSO with or without 10 μM BMS509744 (Tocris, Cat: 5009, Batch: 2B/247519) for 90 min and were dispensed into a U-bottomed 96-well plate at a density of 1 × 10^5^ cells/well in 50 μl lymphocyte medium/well. Raji cells were irradiated at 90 Gy and stained with a CellTrace CFSE dye (Cat: C34554; Invitrogen, 1:1,000 in PBS, 10 min in the incubator) before being dispensed into the plate at a density of 2 × 10^5^ cells/well in 50 μl/well lymphocyte medium. The cells were cocultured in the presence or absence of blinatumomab (Cat: 100441-2; BPS Biosciences, 10 ng/ml) for 24 h. The levels of secreted cytokines and cytotoxic effector molecules were determined with the LEGENDplex Human CD8/NK Panel kit (Cat: 740267; BioLegend). Cell pellets were stained with Live/Dead Aqua dye (Invitrogen) and fixed with BD Cytofix Fixation Buffer (Cat: 554655; BD Biosciences). LEGENDplex beads and fixed cells were acquired with an Attune NxT Flow Cytometer with the CytKick MAX Autosampler (Invitrogen). CFSE^+^ Raji cells were counted with FlowJo.

### Statistical analysis

All statistical analyses were performed in R v.4 (http://www.R-project.org/; R Core Team, 2021). The statistical significance of quantitative differences between groups was assessed in two-tailed non-paired Wilcoxon’s rank-sum tests unless otherwise stated. False discovery rate (FDR) adjustment was performed by the Benjamini and Hochberg method (Benjamini and Hochberg, 1995). P values below 0.05 were considered statistically significant.

### Online supplemental material

The online supplementary information describes the clinical manifestations of P1–P3 in greater depth (Fig. S1) and the virological analysis of P1 and P2 (Fig. S2). Additional analyses on the phosphorylation of ZAP70 in the expanded T lymphocytes from P1 and P2 (Fig. S3), the induction of IL-9, IL-17, and IL-22 and IRF4 in the expanded T lymphocytes from P1 and P2 (Fig. S4), and the cellular response to BCG mycobacteria are also provided. Tables S1 and S2 describe the results of immunological studies for the Iranian (P1 and P2) and Turkish (P3) patients, respectively.

## Supplementary Material

Table S1 describes immunological studies of the Iranian siblings (P1 and P2).

Table S2 describes immunological studies of the Turkish patient (P3). Abnormally low values are shown in red.

SourceData F2 contains original blots for Fig. 2.

SourceData FS3 contains original blots for Fig. S3.

## Data Availability

All raw and processed data are available from the corresponding authors upon request.

## References

[bib1] Abel, L., J. El-Baghdadi, A.A. Bousfiha, J.L. Casanova, and E. Schurr. 2014. Human genetics of tuberculosis: A long and winding road. Philos. Trans. R. Soc. Lond. B: Biol. Sci. 369:20130428. 10.1098/rstb.2013.0428 24821915 PMC4024222

[bib2] Abel, L., J. Fellay, D.W. Haas, E. Schurr, G. Srikrishna, M. Urbanowski, N. Chaturvedi, S. Srinivasan, D.H. Johnson, and W.R. Bishai. 2018. Genetics of human susceptibility to active and latent tuberculosis: Present knowledge and future perspectives. Lancet Infect. Dis. 18:e64–e75. 10.1016/S1473-3099(17)30623-0 29111156 PMC8903186

[bib3] Alme, C., P. Satwani, B. Alobeid, G. Bhagat, and K.M. Kelly. 2015. Atypical clinical course in pediatric Hodgkin lymphoma: Association with germline mutations in interleukin-2-inducible T-cell kinase. J. Pediatr. Hematol. Oncol. 37:507–508. 10.1097/MPH.0000000000000366 26056787

[bib4] Altare, F., A. Ensser, A. Breiman, J. Reichenbach, J.E. Baghdadi, A. Fischer, J.F. Emile, J.L. Gaillard, E. Meinl, and J.L. Casanova. 2001. Interleukin-12 receptor β1 deficiency in a patient with abdominal tuberculosis. J. Infect. Dis. 184:231–236. 10.1086/321999 11424023

[bib5] Belkadi, A., V. Pedergnana, A. Cobat, Y. Itan, Q.B. Vincent, A. Abhyankar, L. Shang, J. El Baghdadi, A. Bousfiha, Exome/Array Consortium, . 2016. Whole-exome sequencing to analyze population structure, parental inbreeding, and familial linkage. Proc. Natl. Acad. Sci. USA. 113:6713–6718. 10.1073/pnas.1606460113 27247391 PMC4914194

[bib6] Bellamy, R. 1998. Genetics and pulmonary medicine. 3. Genetic susceptibility to tuberculosis in human populations. Thorax. 53:588–593. 10.1136/thx.53.7.588 9797760 PMC1745258

[bib7] Benjamini, Y., and Y. Hochberg. 1995. Controlling the false discovery rate: A practical and powerful approach to multiple testing. J. R. Stat. Soc. Ser. B Methodol. 57:289–300. 10.2307/2346101

[bib8] Berg, L.J., L.D. Finkelstein, J.A. Lucas, and P.L. Schwartzberg. 2005. Tec family kinases in T lymphocyte development and function. Annu. Rev. Immunol. 23:549–600. 10.1146/annurev.immunol.22.012703.104743 15771581

[bib9] Béziat, V., F. Rapaport, J. Hu, M. Titeux, M. Bonnet des Claustres, M. Bourgey, H. Griffin, E. Bandet, C.S. Ma, R. Sherkat, . 2021. Humans with inherited T cell CD28 deficiency are susceptible to skin papillomaviruses but are otherwise healthy. Cell. 184:3812–3828.e30. 10.1016/j.cell.2021.06.004 34214472 PMC8329841

[bib10] Bienemann, K., A. Borkhardt, W. Klapper, and I. Oschlies. 2015. High incidence of Epstein-Barr virus (EBV)-positive Hodgkin lymphoma and Hodgkin lymphoma-like B-cell lymphoproliferations with EBV latency profile 2 in children with interleukin-2-inducible T-cell kinase deficiency. Histopathology. 67:607–616. 10.1111/his.12677 25728094

[bib11] Boisson-Dupuis, S., J. Bustamante, J. El-Baghdadi, Y. Camcioglu, N. Parvaneh, S. El Azbaoui, A. Agader, A. Hassani, N. El Hafidi, N.A. Mrani, Z. Jouhadi, . 2015. Inherited and acquired immunodeficiencies underlying tuberculosis in childhood. Immunol. Rev. 264:103–120. 10.1111/imr.12272 25703555 PMC4405179

[bib12] Boisson-Dupuis, S., N. Ramirez-Alejo, Z. Li, E. Patin, G. Rao, G. Kerner, C.K. Lim, D.N. Krementsov, N. Hernandez, C.S. Ma, . 2018. Tuberculosis and impaired IL-23-dependent IFN-γ immunity in humans homozygous for a common TYK2 missense variant. Sci. Immunol. 3:eaau8714. 10.1126/sciimmunol.aau8714 30578352 PMC6341984

[bib13] Boisson-Dupuis, S., and J. Bustamante. 2021. Mycobacterial diseases in patients with inborn errors of immunity. Curr. Opin. Immunol. 72:262–271. 10.1016/j.coi.2021.07.001 34315005 PMC9172628

[bib14] Le Bourhis, L., E. Martin, I. Peguillet, A. Guihot, N. Froux, M. Core, E. Levy, M. Dusseaux, V. Meyssonnier, V. Premel, . 2010. Antimicrobial activity of mucosal-associated invariant T cells. Nat. Immunol. 11:701–708. 10.1038/ni.1890 20581831

[bib15] Broussard, C., C. Fleischacker, C. Fleischecker, R. Horai, M. Chetana, A.M. Venegas, L.L. Sharp, S.M. Hedrick, B.J. Fowlkes, and P.L. Schwartzberg. 2006. Altered development of CD8^+^ T cell lineages in mice deficient for the tec kinases Itk and Rlk. Immunity. 25:93–104. 10.1016/j.immuni.2006.05.011 16860760

[bib16] Bustamante, J. 2020. Mendelian susceptibility to mycobacterial disease: Recent discoveries. Hum. Genet. 139:993–1000. 10.1007/s00439-020-02120-y 32025907 PMC7275902

[bib17] Çaǧdaş, D., B. Erman, D. Hanoglu, B. Tavil, B. Kuşkonmaz, B. Aydin, C. Akyüz, D. Uçkan, Ö. Sanal, and I. Tezcan. 2017. Course of IL-2-inducible T-cell kinase deficiency in a family: Lymphomatoid granulomatosis, lymphoma and allogeneic bone marrow transplantation in one sibling; and death in the other. Bone Marrow Transplant. 52:126–129. 10.1038/bmt.2016.185 27454071

[bib82] Casanova, J.-L., and L. Abel. 2022. From rare disorders of immunity to common determinants of infection: Following teh mechanistic thread. Cell. 185:3086–3103. 10.1016/j.cell.2022.07.004 35985287 PMC9386946

[bib18] Chen, Z.W. 2016. Protective immune responses of major Vγ2Vδ2 T-cell subset in M. tuberculosis infection. Curr. Opinion Immunol. 42:105–112. 10.1016/j.coi.2016.06.005 PMC5754217 27491008

[bib19] Cho, H.-S., S. Ha, H.M. Shin, A. Reboldi, J.A. Hall, J.R. Huh, E.J. Usherwood, and L.J. Berg. 2020. CD8 + T cells Require ITK-mediated TCR signaling for migration to the intestine. ImmunoHorizons. 4:57–71. 10.4049/immunohorizons.1900093 32034085 PMC7521019

[bib20] Cipe, F.E., C. Aydogmus, N.K. Serwas, D. Tuğcu, M. Demirkaya, F.A. Biçici, A.B. Hocaoglu, F. Doğu, and K. Boztuğ. 2015. ITK deficiency: How can EBV be treated before lymphoma? Pediatr. Blood Cancer. 62:2247–2248. 10.1002/pbc.25648 26174447

[bib21] Coffey, F., S.Y. Lee, T.B. Buus, J.P.H. Lauritsen, G.W. Wong, M.L. Joachims, L.F. Thompson, J.C. Zuniga-Pflucker, D.J. Kappes, and D.L. Wiest. 2014. The TCR ligand-inducible expression of CD73 marks γδ lineage commitment and a metastable intermediate in effector specification. J. Exp. Med. 211:329–343. 10.1084/jem.20131540 24493796 PMC3920555

[bib22] Comstock, G.W. 1978. Tuberculosis in twins: A re-analysis of the prophit survey. Am. Rev. Respir. Dis. 117:621–624. 10.1164/arrd.1978.117.4.621 565607

[bib23] Dik, W.A., K. Pike-Overzet, F. Weerkamp, D. de Ridder, E.F. de Haas, M.R. Baert, P. van der Spek, E.E. Koster, M.J. Reinders, J.J. van Dongen, . 2005. New insights on human T cell development by quantitative T cell receptor gene rearrangement studies and gene expression profiling. J. Exp. Med. 201:1715–1723. 10.1084/jem.20042524 15928199 PMC2213269

[bib24] Van Der Eijk, E.A., E. van de Vosse, J.P. Vandenbroucke, and J.T. van Dissel. 2007. Heredity versus environment in tuberculosis in twins: The 1950s United Kingdom prophit survey - simonds and Comstock revisited. Am. J. Respir. Crit. Care Med. 176:1281–1288. 10.1164/rccm.200703-435OC 17823356

[bib25] Eken, A., M. Cansever, I. Somekh, Y. Mizoguchi, N. Zietara, F.Z. Okus, S. Erdem, H. Canatan, S. Akyol, A. Ozcan, . 2019. Genetic deficiency and biochemical inhibition of ITK affect human Th17, treg, and innate lymphoid cells. J. Clin. Immunol. 39:391–400. 10.1007/s10875-019-00632-5 31025232

[bib26] Fang, M., H. Abolhassani, Q. Pan-Hammarström, E. Sandholm, X. Liu, and L. Hammarström. 2019. Compound heterozygous mutations of IL2-inducible T cell kinase in a Swedish patient: The importance of early genetic diagnosis. J. Clin. Immunol. 39:131–134. 10.1007/s10875-019-00598-4 30756212

[bib27] Felices, M., C.C. Yin, Y. Kosaka, J. Kang, and L.J. Berg. 2009. Tec kinase Itk in gammadeltaT cells is pivotal for controlling IgE production in vivo. Proc. Natl. Acad. Sci. USA. 106:8308–8313. 10.1073/pnas.0808459106 19416854 PMC2688853

[bib28] Gangadharan, D., F. Lambolez, A. Attinger, Y. Wang-Zhu, B.A. Sullivan, and H. Cheroutre. 2006. Identification of pre- and postselection TCRalphabeta+ intraepithelial lymphocyte precursors in the thymus. Immunity. 25:631–641. 10.1016/j.immuni.2006.08.018 17045820

[bib29] Gentili, M., J. Kowal, M. Tkach, T. Satoh, X. Lahaye, C. Conrad, M. Boyron, B. Lombard, S. Durand, G. Kroemer, . 2015. Transmission of innate immune signaling by packaging of cGAMP in viral particles. Science. 349:1232–1236. 10.1126/science.aab3628 26229115

[bib30] Ghosh, S., K. Bienemann, K. Boztug, and A. Borkhardt. 2014. Interleukin-2-Inducible T-cell kinase (ITK) deficiency—clinical and molecular Aspects. J. Clin. Immunol. 34:892–899. 10.1007/s10875-014-0110-8 25339095 PMC4220104

[bib31] Ghosh, S., I. Drexler, S. Bhatia, H. Adler, A.R. Gennery, and A. Borkhardt. 2018. Interleukin-2-inducible T-cell kinase deficiency-new patients, new insight? Front. Immunol. 9:979. 10.3389/fimmu.2018.00979 29867957 PMC5951928

[bib32] Gomez-Rodriguez, J., F. Meylan, R. Handon, E.T. Hayes, S.M. Anderson, M.R. Kirby, R.M. Siegel, and P.L. Schwartzberg. 2016. Itk is required for Th9 differentiation via TCR-mediated induction of IL-2 and IRF4. Nat. Commun. 7:10857. 10.1038/ncomms10857 26936133 PMC4782063

[bib33] Haapaniemi, E.M., M. Kaustio, H.L.M. Rajala, A.J. van Adrichem, L. Kainulainen, V. Glumoff, R. Doffinger, H. Kuusanmaki, T. Heiskanen-Kosma, L. Trotta, . 2015. Autoimmunity, hypogammaglobulinemia, lymphoproliferation, and mycobacterial disease in patients with activating mutations in STAT3. Blood. 125:639–648. 10.1182/blood-2014-04-570101 25349174 PMC4304109

[bib34] Han, A., E.W. Newell, J. Glanville, N. Fernandez-Becker, C. Khosla, Y.H. Chien, and M.M. Davis. 2013. Dietary gluten triggers concomitant activation of CD4 + and CD8 + αβ T cells and γδ T cells in celiac disease. Proc. Natl. Acad. Sci. USA. 110:13073–13078. 10.1073/pnas.1311861110 23878218 PMC3740842

[bib35] Hanke, T., R. Mitnacht, R. Boyd, and T. Hünig. 1994. Induction of interleukin 2 receptor beta chain expression by self-recognition in the thymus. J. Exp. Med. 180:1629–1636. 10.1084/jem.180.5.1629 7964450 PMC2191755

[bib36] Hauck, F., C. Randriamampita, E. Martin, S. Gerart, N. Lambert, A. Lim, J. Soulier, Z. Maciorowski, F. Touzot, D. Moshous, . 2012. Primary T-cell immunodeficiency with immunodysregulation caused by autosomal recessive LCK deficiency. J. Allergy Clin. Immunol. 130:1144–1152.e11. 10.1016/j.jaci.2012.07.029 22985903

[bib37] Houben, R.M., and P.J. Dodd. 2016. The global burden of latent tuberculosis infection: A Re-estimation using mathematical modelling. PLoS Med. 13:e1002152. 10.1371/journal.pmed.1002152 PMC5079585 27780211

[bib38] Howe, M.K., K. Dowdell, A. Roy, J.E. Niemela, W. Wilson, J.J. McElwee, J.D. Hughes, and J.I. Cohen. 2019. Magnesium Restores activity to peripheral blood cells in a patient with functionally impaired interleukin-2-inducible T cell kinase. Front. Immunol. 10:2000. 10.3389/fimmu.2019.02000 31507602 PMC6718476

[bib39] Huang, L., K. Ye, M.C. McGee, N.F. Nidetz, J.P. Elmore, C.B. Limper, T.L. Southard, D.G. Russell, A. August, and W. Huang. 2019. Interleukin-2-Inducible T-cell kinase deficiency impairs early pulmonary protection against Mycobacterium tuberculosis infection. Front. Immunol. 10:3103. 10.3389/fimmu.2019.03103 32038633 PMC6993117

[bib40] Huck, K., O. Feyen, T. Niehues, F. Ruschendorf, N. Hubner, H.J. Laws, T. Telieps, S. Knapp, H.H. Wacker, A. Meindl, . 2009. Girls homozygous for an IL-2-inducible T cell kinase mutation that leads to protein deficiency develop fatal EBV-associated lymphoproliferation. J. Clin. Invest. 119:1350–1358. 10.1172/JCI37901 19425169 PMC2673872

[bib41] Itan, Y., L. Shang, B. Boisson, M.J. Ciancanelli, J.G. Markle, R. Martinez-Barricarte, E. Scott, I. Shah, P.D. Stenson, J. Gleeson, . 2016. The mutation significance cutoff: Gene-level thresholds for variant predictions. Nat. Methods. 13:109–110. 10.1038/nmeth.3739 26820543 PMC4980758

[bib42] Iyer, A.S., and A. August. 2008. The tec family kinase, IL-2-inducible T cell kinase, differentially controls mast cell responses. J. Immunol. 180:7869–7877. 10.4049/jimmunol.180.12.7869 18523250 PMC2583454

[bib43] Kallmann, F.J., and D. Reisner. 1943. Twin studies on the significance of genetic factors in tuberculosis. Am. Rev. Tuberc. Pulm. Dis. 47:549–571. 10.1164/art.1943.47.6.549

[bib44] Kannan, A., Y. Lee, Q. Qi, W. Huang, A.R. Jeong, S. Ohnigian, and A. August. 2015. Allele-sensitive mutant, Itkas, reveals that Itk kinase activity is required for Th1, Th2, Th17, and iNKT-cell cytokine production. Eur. J. Immunol. 45:2276–2285. 10.1002/eji.201445087 25989458 PMC5730406

[bib45] Kannan, A.K., N. Sahu, S. Mohanan, S. Mohinta, and A. August. 2013. IL-2–inducible T-cell kinase modulates TH2-mediated allergic airway inflammation by suppressing IFN-γ in naive CD4^+^ T cells. J. Allergy Clin. Immunol. 132:811–820.e1-5. 10.1016/j.jaci.2013.04.033 23768572 PMC4033298

[bib46] Kerner, G., N. Ramirez-Alejo, Y. Seeleuthner, R. Yang, M. Ogishi, A. Cobat, E. Patin, L. Quintana-Murci, S. Boisson-Dupuis, J.L. Casanova, and L. Abel. 2019. Homozygosity for TYK2 P1104A underlies tuberculosis in about 1% of patients in a cohort of European ancestry. Proc. Natl. Acad. Sci. USA. 116:10430–10434. 10.1073/pnas.1903561116 31068474 PMC6534977

[bib47] Kerner, G., J. Rosain, A. Guerin, A. Al-Khabaz, C. Oleaga-Quintas, F. Rapaport, M.J. Massaad, J.Y. Ding, T. Khan, F.A. Ali, . 2020. Inherited human IFN-γ deficiency underlies mycobacterial disease. J. Clin. Invest. 130:3158–3171. 10.1172/jci135460 32163377 PMC7260033

[bib48] Kerner, G., G. Laval, E. Patin, S. Boisson-Dupuis, L. Abel, J.L. Casanova, and L. Quintana-Murci. 2021. Human ancient DNA analyses reveal the high burden of tuberculosis in Europeans over the last 2, 000 years. Am. J. Hum. Genet. 108:517–524. 10.1016/j.ajhg.2021.02.009 33667394 PMC8008489

[bib49] Khurana, D., L.N. Arneson, R.A. Schoon, C.J. Dick, and P.J. Leibson. 2007. Differential regulation of human NK cell-mediated cytotoxicity by the tyrosine kinase Itk. J. Immunol. 178:3575–3582. 10.4049/jimmunol.178.6.3575 17339454

[bib50] Kircher, M., D.M. Witten, P. Jain, B.J. O'Roak, G.M. Cooper, and J. Shendure. 2014. A general framework for estimating the relative pathogenicity of human genetic variants. Nat. Genet. 46:310–315. 10.1038/ng.2892 24487276 PMC3992975

[bib51] Klose, C.S.N., K. Blatz, Y. d'Hargues, P.P. Hernandez, M. Kofoed-Nielsen, J.F. Ripka, K. Ebert, S.J. Arnold, A. Diefenbach, E. Palmer, and Y. Tanriver. 2014. The transcription factor T-bet is induced by IL-15 and thymic agonist selection and controls CD8αα+ intraepithelial lymphocyte development. Immunity. 41:230–243. 10.1016/j.immuni.2014.06.018 25148024

[bib52] Kreins, A.Y., M.J. Ciancanelli, S. Okada, X.F. Kong, N. Ramírez-Alejo, S.S. Kilic, J. El Baghdadi, S. Nonoyama, S.A. Mahdaviani, F. Ailal, . 2015. Human TYK2 deficiency: Mycobacterial and viral infections without hyper-IgE syndrome. J. Exp. Med. 212:1641–1662. 10.1084/jem.20140280 26304966 PMC4577846

[bib53] Kreslavsky, T., A.K. Savage, R. Hobbs, F. Gounari, R. Bronson, P. Pereira, P.P. Pandolfi, A. Bendelac, and H. von Boehmer. 2009. TCR-inducible PLZF transcription factor required for innate phenotype of a subset of γδ T cells with restricted TCR diversity. Proc. Natl. Acad. Sci. USA. 106:12453–12458. 10.1073/pnas.0903895106 19617548 PMC2718370

[bib54] Liao, X.C., and D.R. Littman. 1995. Altered T cell receptor signaling and disrupted T cell development in mice lacking Itk. Immunity. 3:757–769. 10.1016/1074-7613(95)90065-9 8777721

[bib55] Linka, R.M., S.L. Risse, K. Bienemann, M. Werner, Y. Linka, F. Krux, C. Synaeve, R. Deenen, S. Ginzel, R. Dvorsky, . 2012. Loss-of-function mutations within the IL-2 inducible kinase ITK in patients with EBV-associated lymphoproliferative diseases. Leukemia. 26:963–971. 10.1038/leu.2011.371 22289921

[bib56] Maccari, M.E., S. Fuchs, P. Kury, G. Andrieux, S. Volkl, B. Bengsch, M.R. Lorenz, M. Heeg, J. Rohr, S. Jagle, . 2020. A distinct CD38^+^CD45RA^+^ population of CD4^+^, CD8^+^, and double-negative T cells is controlled by FAS. J. Exp. Med. 218:e20192191. 10.1084/jem.20192191 PMC7658692 33170215

[bib57] Maffucci, P., B. Bigio, F. Rapaport, A. Cobat, A. Borghesi, M. Lopez, E. Patin, A. Bolze, L. Shang, M. Bendavid, . 2019. Blacklisting variants common in private cohorts but not in public databases optimizes human exome analysis. Proc. Natl. Acad. Sci. USA. 116:950–959. 10.1073/pnas.1808403116 30591557 PMC6338851

[bib58] Majzoobi, M.M., S. Akbarzadeh, G. Ebrahimi, H.R. Ghasemibasir, and P. Alirezaei. 2019. Tuberculous Uveitis, erythema induratum, and persistent genital warts in a female patient: A rare case report. Adv. Biomed. Res. 8:70. 10.4103/abr.abr_154_19 31897408 PMC6909546

[bib59] Mansouri, D., S.A. Mahdaviani, S. Khalilzadeh, S.A. Mohajerani, M. Hasanzad, S. Sadr, S.A. Nadji, S. Karimi, A. Droodinia, N. Rezaei, . 2012. IL-2-inducible T-cell kinase deficiency with pulmonary manifestations due to disseminated epstein-barr virus infection. Int. Arch. Allergy Immunol. 158:418–422. 10.1159/000333472 22487848

[bib83] Martin-Fernandez, M., S. Buta, T. Le Voyer, Z. Li, L.T. Dynesen, F. Vuillier, F. Ailal, A.M. Amancio, L. Malle, C. Gruber, . 2022. A partial form of inherited human USP18 deficiency underlies infection and inflammation. J. Exp. Med. 219(4):e20211273. . 10.1084/jem.20211273 35258551 PMC8908790

[bib60] McDonald, B.D., J.J. Bunker, I.E. Ishizuka, B. Jabri, and A. Bendelac. 2014. Elevated T cell receptor signaling identifies a thymic precursor to the TCRαβ^+^CD4^−^CD8β^−^ intraepithelial lymphocyte lineage. Immunity. 41:219–229. 10.1016/j.immuni.2014.07.008 25131532 PMC4188477

[bib61] McHenry, M.L., J. Bartlett, R.P. Igo Jr, E.M. Wampande, P. Benchek, H. Mayanja-Kizza, K. Fluegge, N.B. Hall, S. Gagneux, S.A. Tishkoff, . 2020. Interaction between host genes and Mycobacterium tuberculosis lineage can affect tuberculosis severity: Evidence for coevolution? PLoS Genet. 16:e1008728. 10.1371/journal.pgen.1008728 PMC7217476 32352966

[bib62] Mo, W., W. Wei, Y. Sun, Y. Yang, Z. Guan, M. Li, P. Zhu, and Z. Chi. 2019. Application of blood and immunodeficiency gene detection in the diagnosis of hemophagocytic lymphohistiocytosis patients. Exp. Hematol. 78:62–69. 10.1016/j.exphem.2019.09.024 31562900

[bib63] Ogishi, M., R. Yang, C. Aytekin, D. Langlais, M. Bourgey, T. Khan, F.A. Ali, M. Rahman, O.M. Delmonte, M. Chrabieh, . 2021. Inherited PD-1 deficiency underlies tuberculosis and autoimmunity in a child. Nat. Med. 27:1646–1654. 10.1038/s41591-021-01388-5 34183838 PMC8446316

[bib64] Ogishi, M., A.A. Arias, R. Yang, J.E. Han, P. Zhang, D. Rinchai, J. Halpern, J. Mulwa, N. Keating, M. Chrabieh, . 2022. Impaired IL-23-dependent induction of IFN-γ underlies mycobacterial disease in patients with inherited TYK2 deficiency. J. Exp. Med. 219:e20220094. 10.1084/jem.20220094 PMC9472563 36094518

[bib65] Okada, S., J.G. Markle, E.K. Deenick, F. Mele, D. Averbuch, M. Lagos, M. Alzahrani, S. Al-Muhsen, R. Halwani, C.S. Ma, . 2015. IMMUNODEFICIENCIES. Impairment of immunity to Candida and Mycobacterium in humans with bi-allelic RORC mutations. Science. 349:606–613. 10.1126/science.aaa4282 26160376 PMC4668938

[bib66] Patsoukis, N., J. Brown, V. Petkova, F. Liu, L. Li, and V.A. Boussiotis. 2012. Selective effects of PD-1 on Akt and Ras pathways regulate molecular components of the cell cycle and inhibit T cell proliferation. Sci. Signal. 5:ra46. 10.1126/scisignal.2002796 22740686 PMC5498435

[bib67] Qiu, L., Y. Wang, W. Tang, Q. Yang, T. Zeng, J. Chen, X. Chen, L. Zhang, L. Zhou, Z. Zhang, . 2022. Activated phosphoinositide 3-kinase δ syndrome: A large pediatric cohort from a single center in China. J. Clin. Immunol. 42:837–850. 10.1007/s10875-022-01218-4 35296988

[bib68] R Core Team. 2021. R: A Language and Environment for Statistical Computing. R Foundation for Statistical Computing, Vienna, Austria.

[bib69] Rapaport, F., B. Boisson, A. Gregor, V. Beziat, S. Boisson-Dupuis, J. Bustamante, E. Jouanguy, A. Puel, J. Rosain, Q. Zhang, . 2021. Negative selection on human genes underlying inborn errors depends on disease outcome and both the mode and mechanism of inheritance. Proc. Natl. Acad. Sci. USA. 118:e2001248118. 10.1073/pnas.2001248118 33408250 PMC7826345

[bib70] Rensing-Ehl, A., S. Völkl, C. Speckmann, M.R. Lorenz, J. Ritter, A. Janda, M. Abinun, H. Pircher, B. Bengsch, R. Thimme, . 2014. Abnormally differentiated CD4^+^ or CD8^+^ T cells with phenotypic and genetic features of double negative T cells in human Fas deficiency. Blood. 124:851–860. 10.1182/blood-2014-03-564286 24894771

[bib71] Rigby, S., Y. Huang, B. Streubel, A. Chott, M.Q. Du, S.D. Turner, and C.M. Bacon. 2009. The lymphoma-associated fusion tyrosine kinase ITK-SYK requires pleckstrin homology domain-mediated membrane localization for activation and cellular transformation. J. Biol. Chem. 284:26871–26881. 10.1074/jbc.M109.034272 19535334 PMC2785375

[bib72] Schaeffer, E.M., G.S. Yap, C.M. Lewis, M.J. Czar, D.W. McVicar, A.W. Cheever, A. Sher, and P.L. Schwartzberg. 2001. Mutation of Tec family kinases alters T helper cell differentiation. Nat. Immunol. 2:1183–1188. 10.1038/ni734 11702066

[bib73] Schoch, C.L., S. Ciufo, M. Domrachev, C.L. Hotton, S. Kannan, R. Khovanskaya, D. Leipe, R. Mcveigh, K. O'Neill, B. Robbertse, . 2020. NCBI Taxonomy: A comprehensive update on curation, resources and tools. Database. 2020:baaa062. 10.1093/database/baaa062 32761142 PMC7408187

[bib74] Serwas, N.K., D. Cagdas, S.A. Ban, K. Bienemann, E. Salzer, I. Tezcan, A. Borkhardt, O. Sanal, and K. Boztug. 2014. Identification of ITK deficiency as a novel genetic cause of idiopathic CD4^+^ T-cell lymphopenia. Blood. 124:655–657. 10.1182/blood-2014-03-564930 25061172 PMC4110665

[bib75] Stepensky, P., M. Weintraub, A. Yanir, S. Revel-Vilk, F. Krux, K. Huck, R.M. Linka, A. Shaag, O. Elpeleg, A. Borkhardt, and I.B. Resnick. 2011. IL-2-inducible T-cell kinase deficiency: Clinical presentation and therapeutic approach. Haematologica. 96:472–476. 10.3324/haematol.2010.033910 21109689 PMC3046282

[bib76] Le Voyer, T., A.L. Neehus, R. Yang, M. Ogishi, J. Rosain, F. Alroqi, M. Alshalan, S. Blumental, F. Al Ali, T. Khan, . 2021. Inherited deficiency of stress granule ZNFX1 in patients with monocytosis and mycobacterial disease. Proc. Natl. Acad. Sci. USA. 118:e2102804118. 10.1073/pnas.2102804118 33876776 PMC8053974

[bib77] Vynnycky, E., and P.E. Fine. 2000. Lifetime Risks, incubation period, and serial interval of tuberculosis. Am. J. Epidemiol. 152:247–263. 10.1093/aje/152.3.247 10933272

[bib78] Wallace, J.G., M.F. Alosaimi, C.D. Khayat, F. Jaber, A. Almutairi, S. Beaussant-Cohen, G. Pinkus, M. Fleming, C. Mehawej, J. Chou, and R.S. Geha. 2020. ITK deficiency presenting as autoimmune lymphoproliferative syndrome. J. Allergy Clin. Immunol. 147:743–745.e1. 10.1016/j.jaci.2020.06.019 32628964 PMC7779661

[bib79] WHO. 2020. Global tuberculosis Report 2020. World Health Organization.

[bib80] Yang, R., F. Mele, L. Worley, D. Langlais, J. Rosain, I. Benhsaien, H. Elarabi, C.A. Croft, J.M. Doisne, P. Zhang, . 2020. Human T-bet governs innate and innate-like adaptive IFN-γ immunity against mycobacteria. Cell. 183:1826–1847.e31. 10.1016/j.cell.2020.10.046 33296702 PMC7770098

[bib81] Youssefian, L., H. Vahidnezhad, M. Yousefi, A.H. Saeidian, A. Azizpour, A. Touati, N. Nikbakht, K.K. Hesari, M.M. Adib-Sereshki, S. Zeinali, . 2019. Inherited interleukin 2-inducible T-cell (ITK) kinase deficiency in siblings with epidermodysplasia verruciformis and Hodgkin lymphoma. Clin. Infect. Dis. 68:1938–1941. 10.1093/cid/ciy942 30778533 PMC7317279

