# Supplementary material for: Inherited human ITK deficiency impairs IFN-γ immunity and underlies tuberculosis
Source: J Exp Med. 2022 Nov 3;220(1):e20220484. doi: 10.1084/jem.20220484 (PMC9641312; doi:10.1084/jem.20220484)
Supplement: Table S1 — describes immunological studies of the Iranian siblings (P1 and P2). [file JEM_20220484_TableS1.docx]

Table S1. Immunological studies of the Iranian siblings (P1 and P2)

|  | **Reference range**  **(adult)** | **Father** | | **Reference range**  **(age-matched)** | **P1** | | **P2** |
| --- | --- | --- | --- | --- | --- | --- | --- |
| Age (yr) |  | 43 | 45 |  | 18 | 19 | 16 |
| Lymphocytes/mm^3^ | 1,500–3,000 | 6,800 | 2,700 | 1,500–3000 | 7,800 | 3,800 | 1,400 |
| T lymphocytes/mm^3^ (%)  CD3^+^  CD4^+^  CD8^+^  αβTCR^+^  γδTCR^+^  CD4^+^CD45RA^+^  CD4^+^CD45RA^+^CD31^+^  CD4^+^CD45RO^+^  CD8^+^CD45RA^+^CCR7^+^  CD8^+^CD45RA^−^CCR7^+^  CD8^+^CD45RA^−^CCR7^−^  CD8^+^CD45RA^+^CCR7^−^ | 807–1,844 (64–85%)  460–1,232 (34–62%)  187–844 (14–42%)  85–99%  1–15%  20–28%  29–57%  3–14%  20–41%  11–26% | 5,984 (88%)  2,788 (41%)  2,992 (44%) | 2,268 (84%)  1,161 (43%)  972 (36%)  94%  6%  41%  17%  59%  18%  6%  26%  50% | 807–1,844 (64–85%)  460–1,232 (34–62%)  187–844 (14–42%)  85–99%  1–15%  58–70%  43–55%  27–50%  52–68%  3–4%  11–20%  16–28% | 5,850 (75%)  546 (7%)  3,198 (41%) | 2,470 (65%)  684 (18%)  950 (25%)  75%  24%  9%  2%  91%  2%  1%  30%  68% | 910 (65%)  406 (29%)  238 (17%)  77%  23%  19%  10%  81%  8%  9%  70%  17% |
| B lymphocytes/mm^3^ (%)  CD19^+^  CD19^+^CD27^+^ | 92–420 (6–17%)  >30% | 544 (8%) | 297 (11%)  41% | 92–420 (6–17%)  10–20% | 468 (6%) | 912 (24%)  8% | 294 (21%)  6% |
| NK lymphocytes/mm^3^ (%) | 89–362 (5–20%) | 272 (4%) | 135 (5%) | 89–362 (5–20%) | 1,014 (13%) | 418 (11%) | 196 (14%) |
| MAIT/10^6^ CD3^+^ T | >8,000 |  | 12,543 | >8,000 |  | 4,880 |  |
| NKT/10^6^ CD3^+^ T | >200 |  | 1,680 | >200 |  | 211 |  |
| T cell proliferation (cpm/10^3^)  PHA  OKT3 (50 ng/ml)  Tetanus Ag  *Candida* Ag  PPD | >50  >30  >10  >10  >10 |  | 80  25  6  26  10 | >50  >30  >10  >10  >10 |  | 13  7  5  14  14 | 23  2.5  1.3  23.5  47 |
