# Supplementary material for: Inherited human ITK deficiency impairs IFN-γ immunity and underlies tuberculosis
Source: J Exp Med. 2022 Nov 3;220(1):e20220484. doi: 10.1084/jem.20220484 (PMC9641312; doi:10.1084/jem.20220484)
Supplement: Table S2 — describes immunological studies of the Turkish patient (P3). Abnormally low values are shown in red. [file JEM_20220484_TableS2.docx]

Table S2. Immunological studies of the Turkish patient (P3)

|  | **1st admission** | **2nd** | **3rd** | **4th** |
| --- | --- | --- | --- | --- |
|  | (4 yr & 4 mo) | (4 yr & 5 mo) | (4 yr & 9 mo) | (4 yr & 10 mo) |
| **WBC (cells/ml)** | 14,700 | 9,570 | 8,760 | 3,130 |
| **Neutrophils (cells/ml)** | 8,930 | 5,270 | 4,350 | 980 |
| **Lymphocytes (cells/ml)** | 4,460 | 3,480 | 2,710 | 1,530 |
| **Eosinophils (cells/ml)** | 350 | 30 | 600 | 10 |
| **Hemoglobin (g/dl)** | 11 | 10 | 8.6 | 7.3 |
| **Platelets (cells/ml)** | 340,000 | 331,000 | 244,000 | 83,000 |
| **IgG (mg/dl)** | 1,150 | 996 | 748 |  |
| **IgA (mg/dl)** | 81 | 45 | 33 |  |
| **IgM (mg/dl)** | 49 | 32 | 7 |  |
| **IgE (IU/ml)** | <16 | <16 | <16 |  |
| **Tetanus Ab titer (IU/ml)** | 0.4 |  |  |  |
| **Pneumococcal Ab titer (µg/ml)** | 3.0 |  |  |  |
| **Isohemagglutinin titer** | 1/4 |  |  |  |
| **HBs Ab (IU/liter)** | 0.96 |  |  |  |
| **HSV Ab** | Negative |  |  |  |
| **VZV Ab** | Negative |  |  |  |
| **Parvovirus B19 IgM** |  |  | Negative |  |
| **Parvovirus B19 IgG** |  |  | Negative |  |
| **EBV-VCA IgM** |  |  | Negative |  |
| **EBV-VCA IgG** |  |  | Negative |  |
| **EBNA** |  |  | Negative |  |
| **CMV IgM** |  |  | Negative |  |
| **CMV IgG** |  |  | Positive |  |
| **CMV DNA** |  |  | Negative |  |
| **CMV Ab (IU/ml)** | 155 |  |  |  |
| **Measles Ab (IU/ml)** | 21 |  |  |  |
| **Mumps Ab (IU/ml)** | 13 |  |  |  |
| **Rubella Ab (IU/ml)** | 144 |  |  |  |
| **CD3^+^ %** | 53 | 62 | 57 | 52 |
| **CD3^+^CD4^+^ %** | 17 | 21 | 26 | 17 |
| **CD3^+^CD8^+^ %** | 24 | 30 | 20 | 27 |
| **CD19^+^ %** | 21 | 15 | 29 | 16 |
| **CD16^+^CD56^+^ %** | 28 | 24 | 13 | 28 |
| **CD4^+^CD45RA^+^CD31^+^ %** | 34 | 28 | 36 | 28 |
| **CD19^+^CD27^+^IgD^–^ %** | 0.7 | 0.4 | 0.2 | 0.1 |

Abnormally low values are shown in red.
