# Supplementary material for: Inherited human ITK deficiency impairs IFN-γ immunity and underlies tuberculosis
Source: J Exp Med. 2022 Nov 3;220(1):e20220484. doi: 10.1084/jem.20220484 (PMC9641312; doi:10.1084/jem.20220484)
Supplement: SourceData F2 — contains original blots for Fig. 2. [file JEM_20220484_SourceDataF2.pdf]

Figure 2A

ITK (Nter)

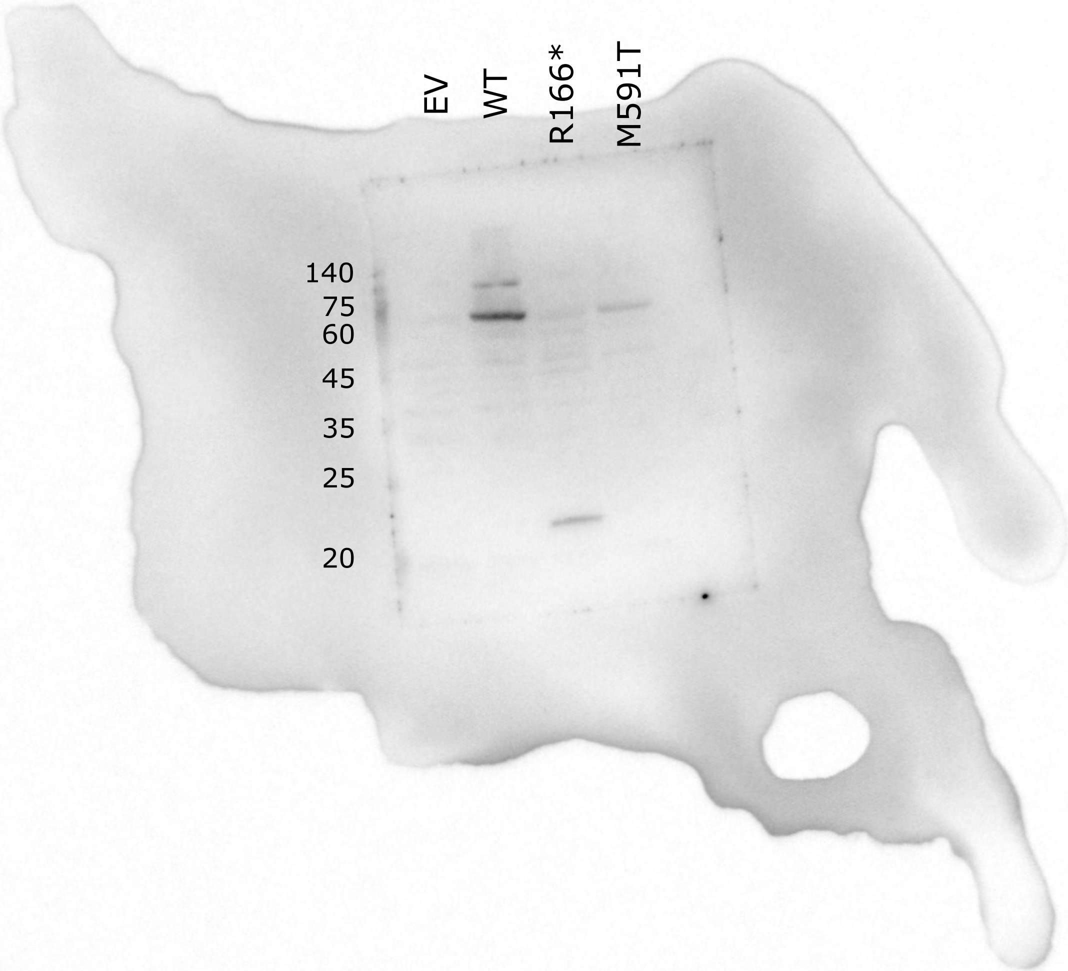

Figure 2B

ITK (Cter)

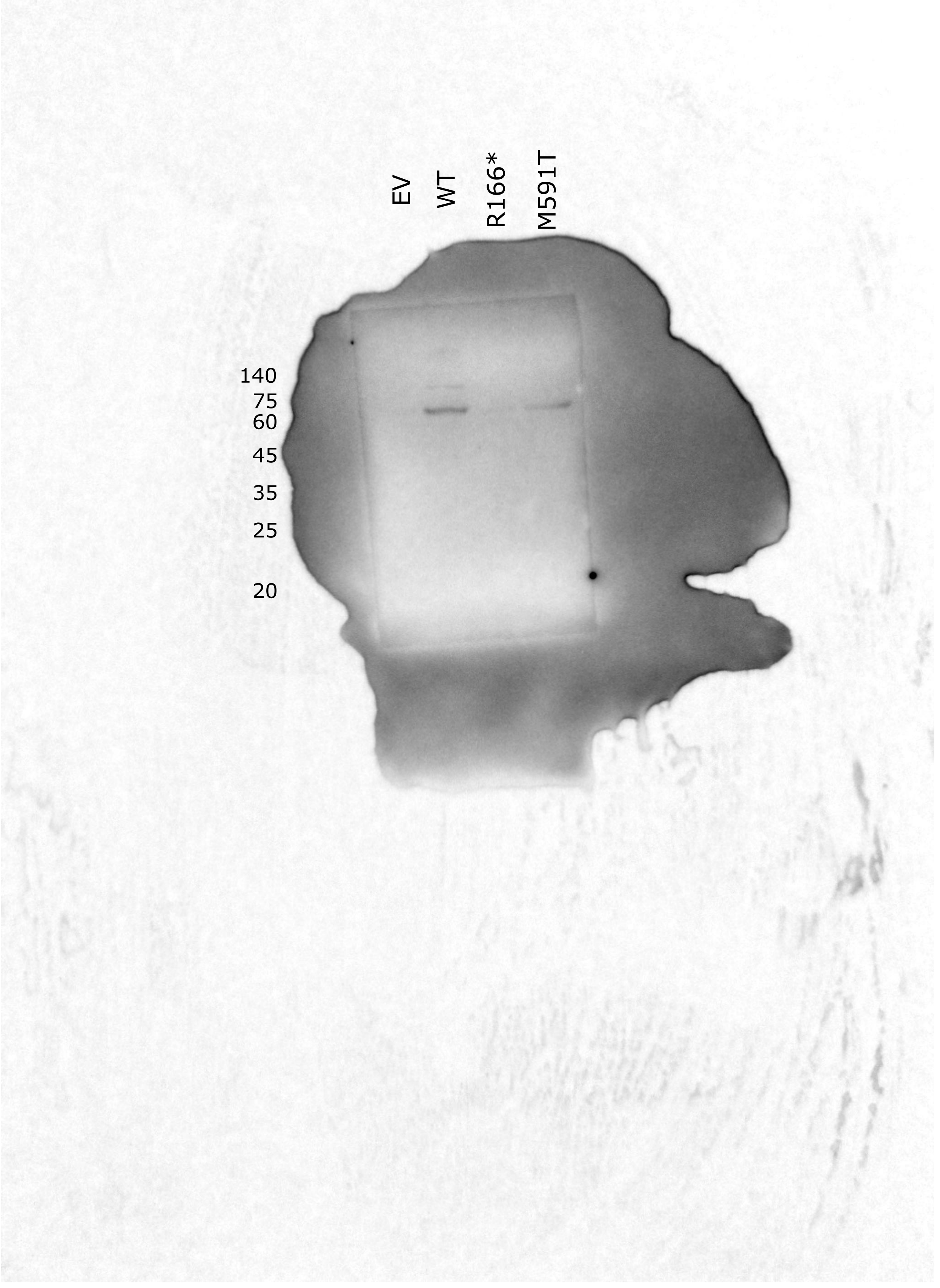

Figure 2A and B

Vinculin

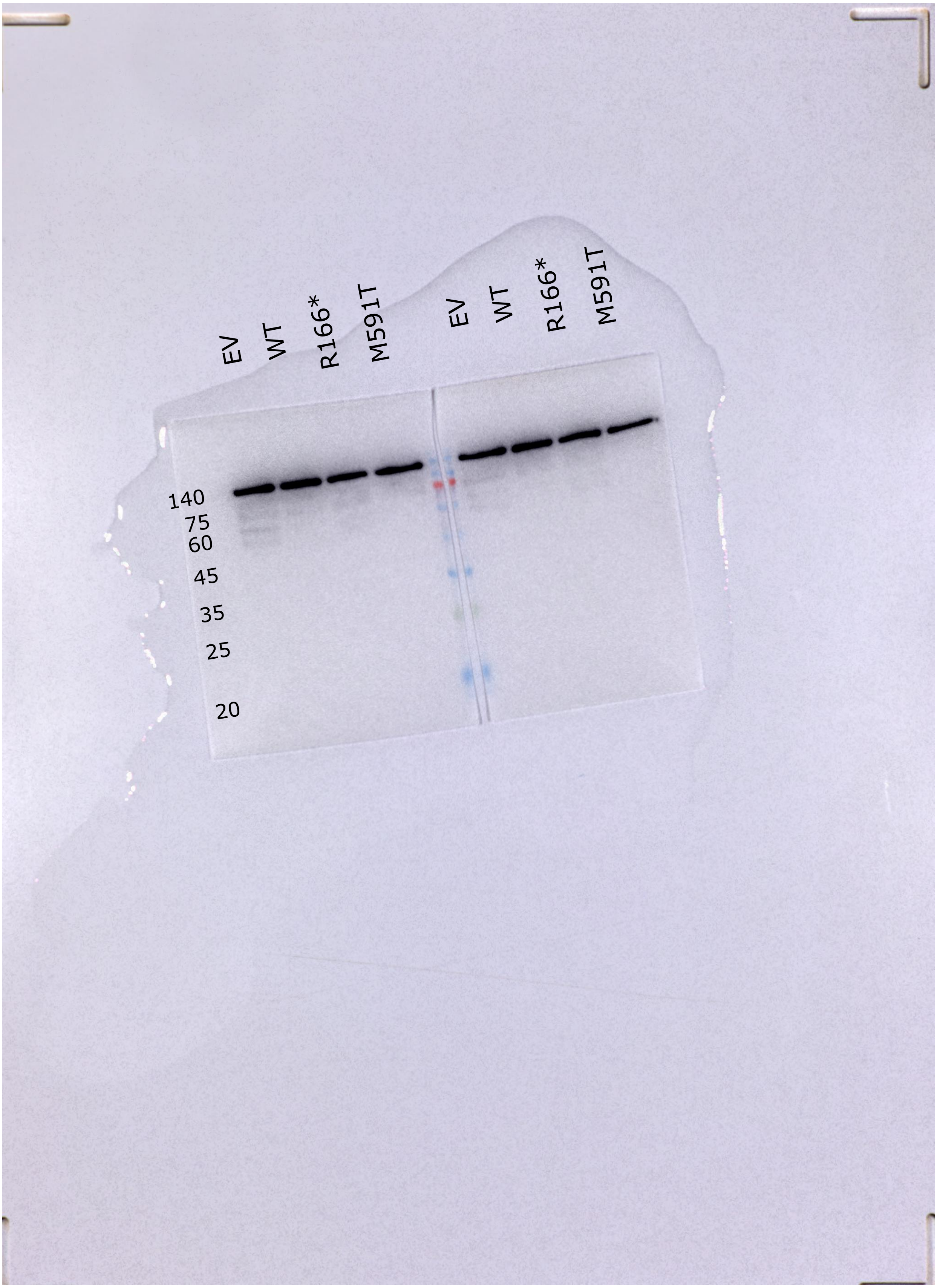

Figure 2C

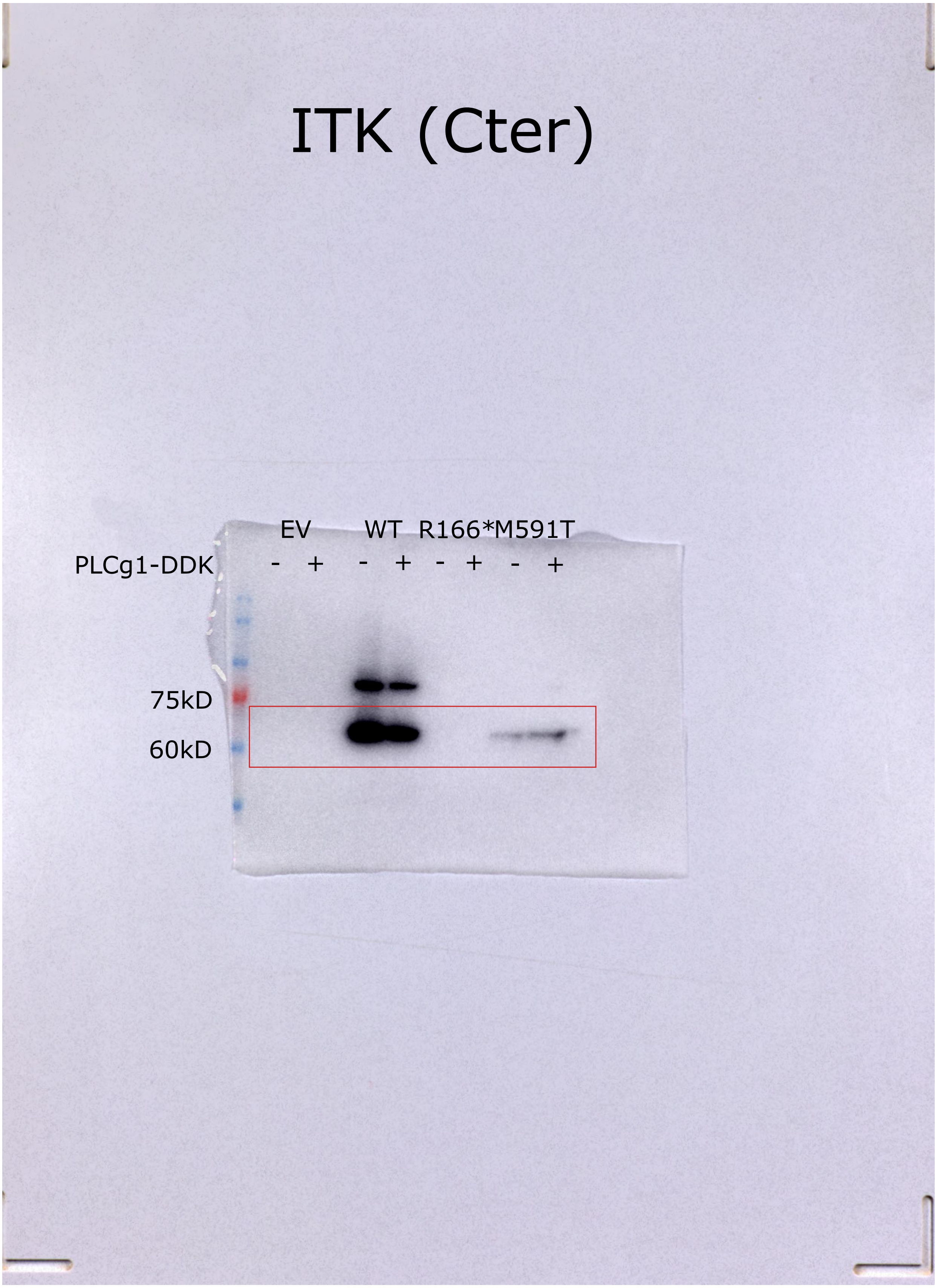

p-PLCg1

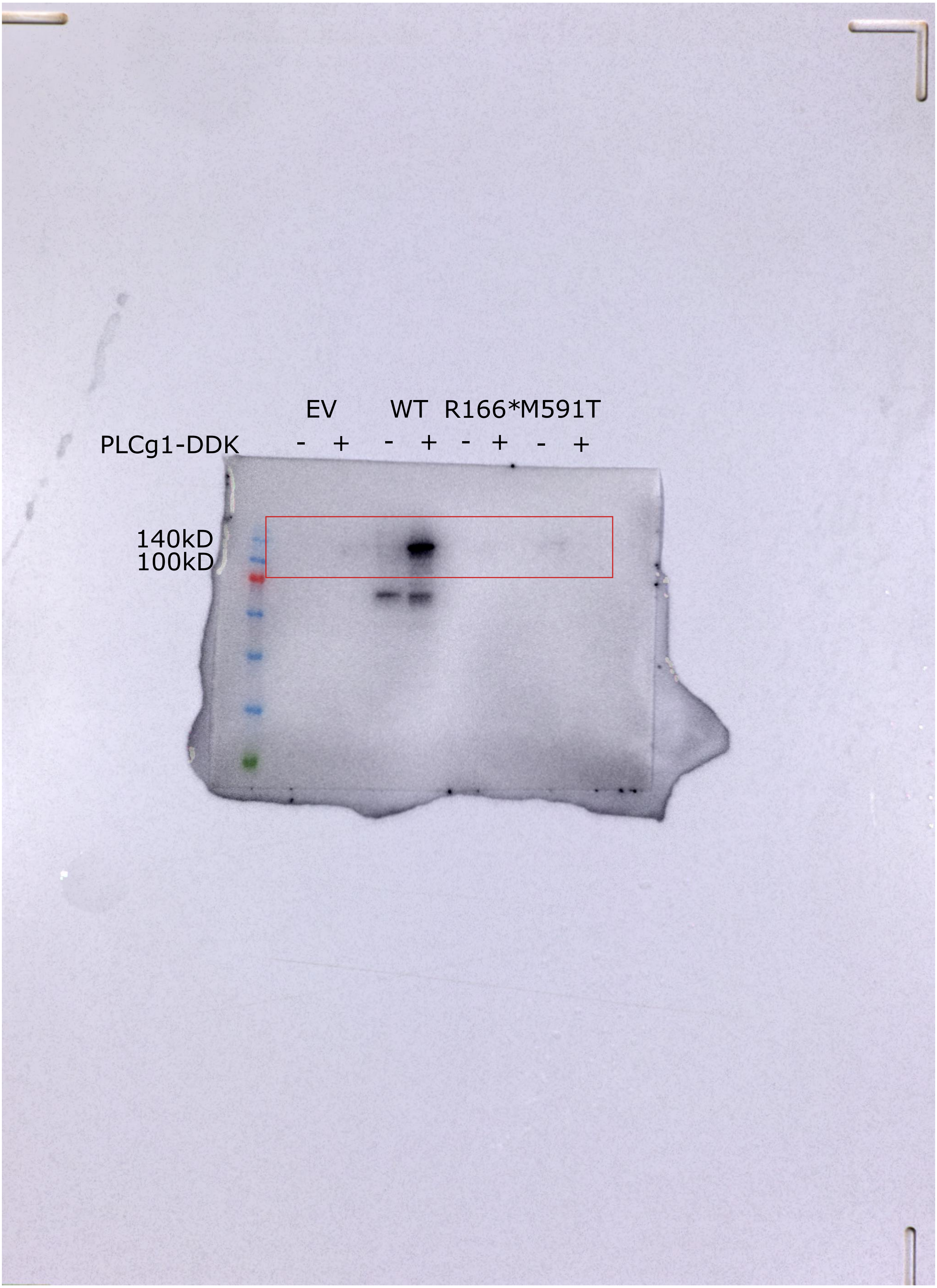

Vinculin

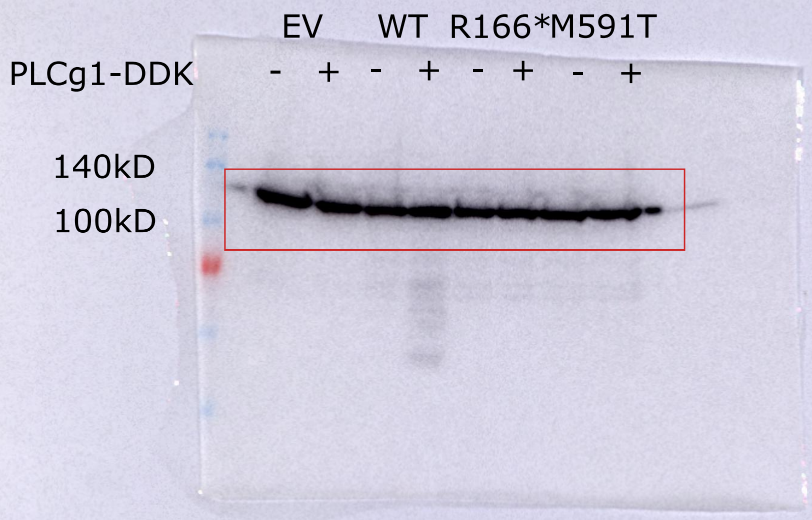

Anti-DDK (PLCg1-DDK)

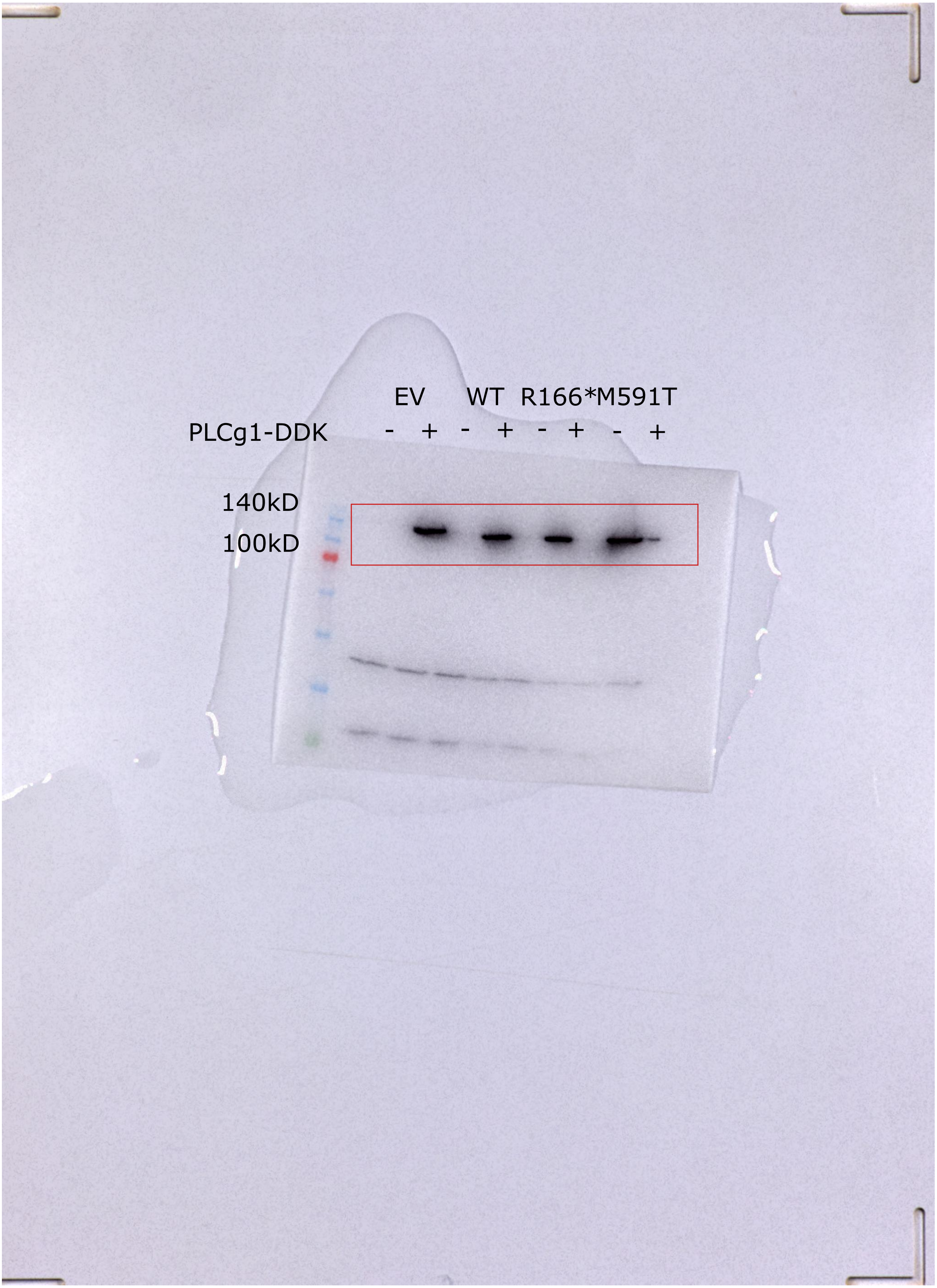

Figure 2D

ITK (Cter) KU70

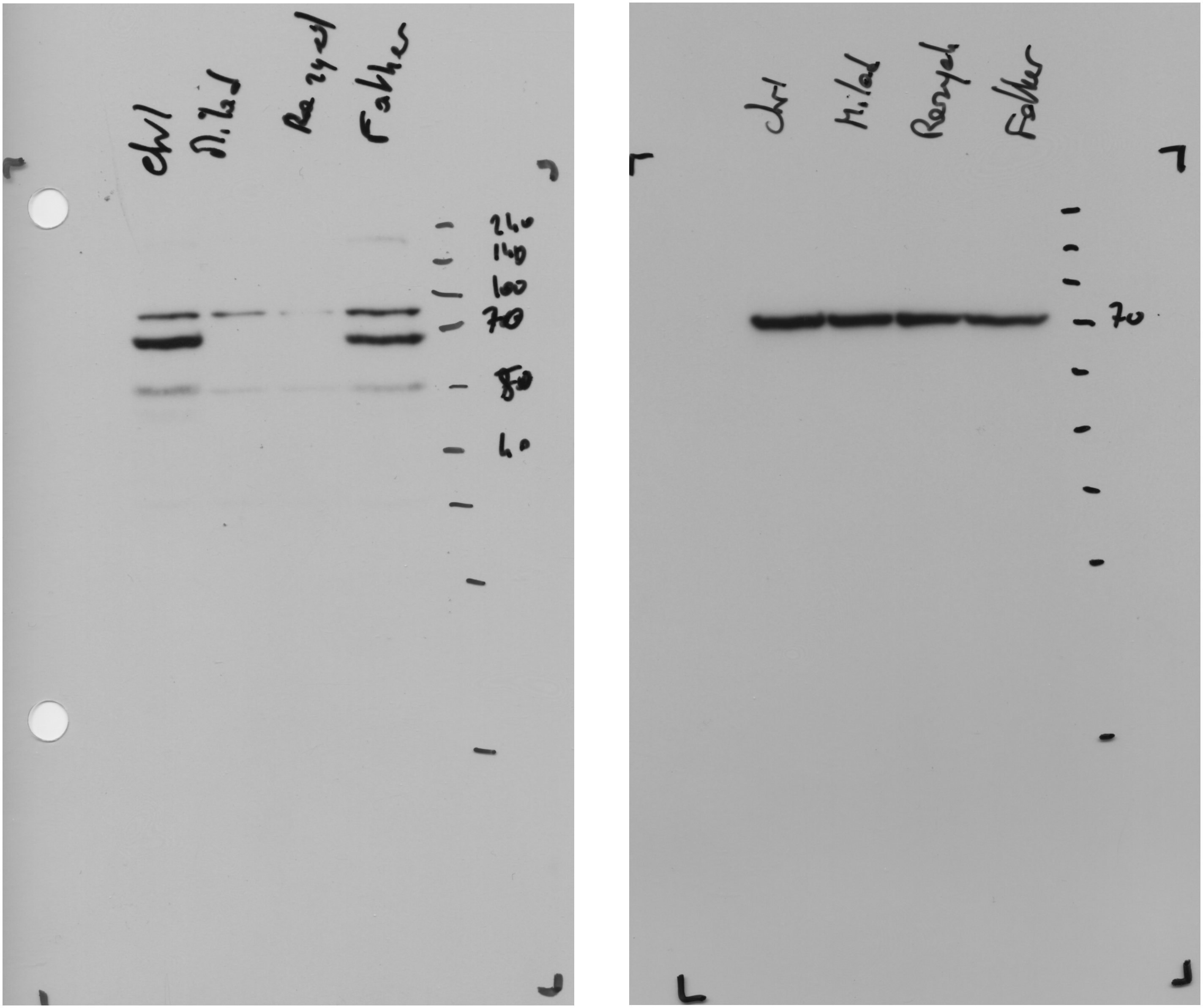

Figure 2E

ITK (Nter)

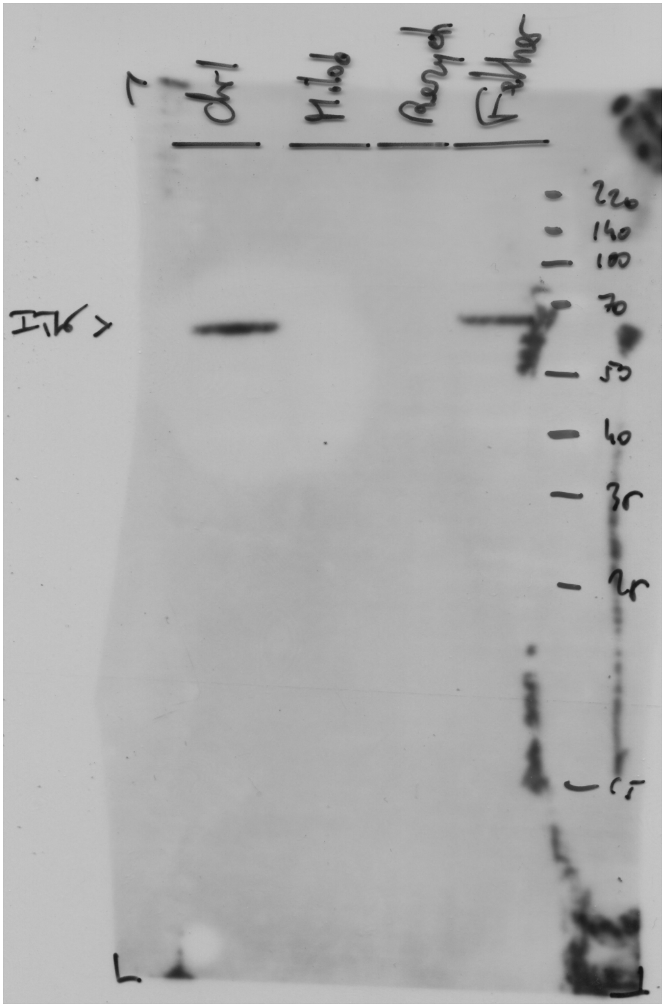

KU70

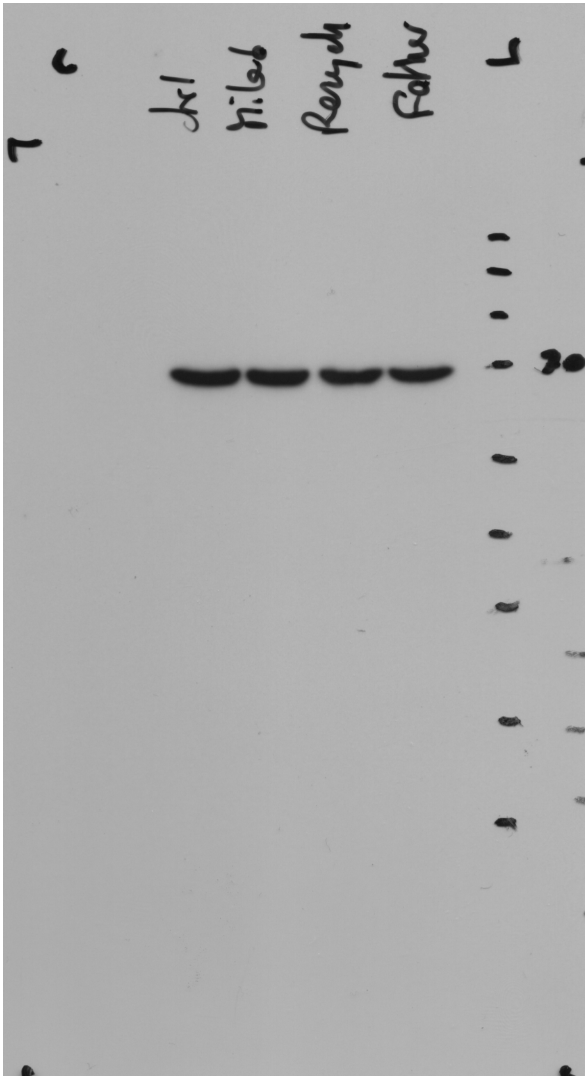

Figure 2F

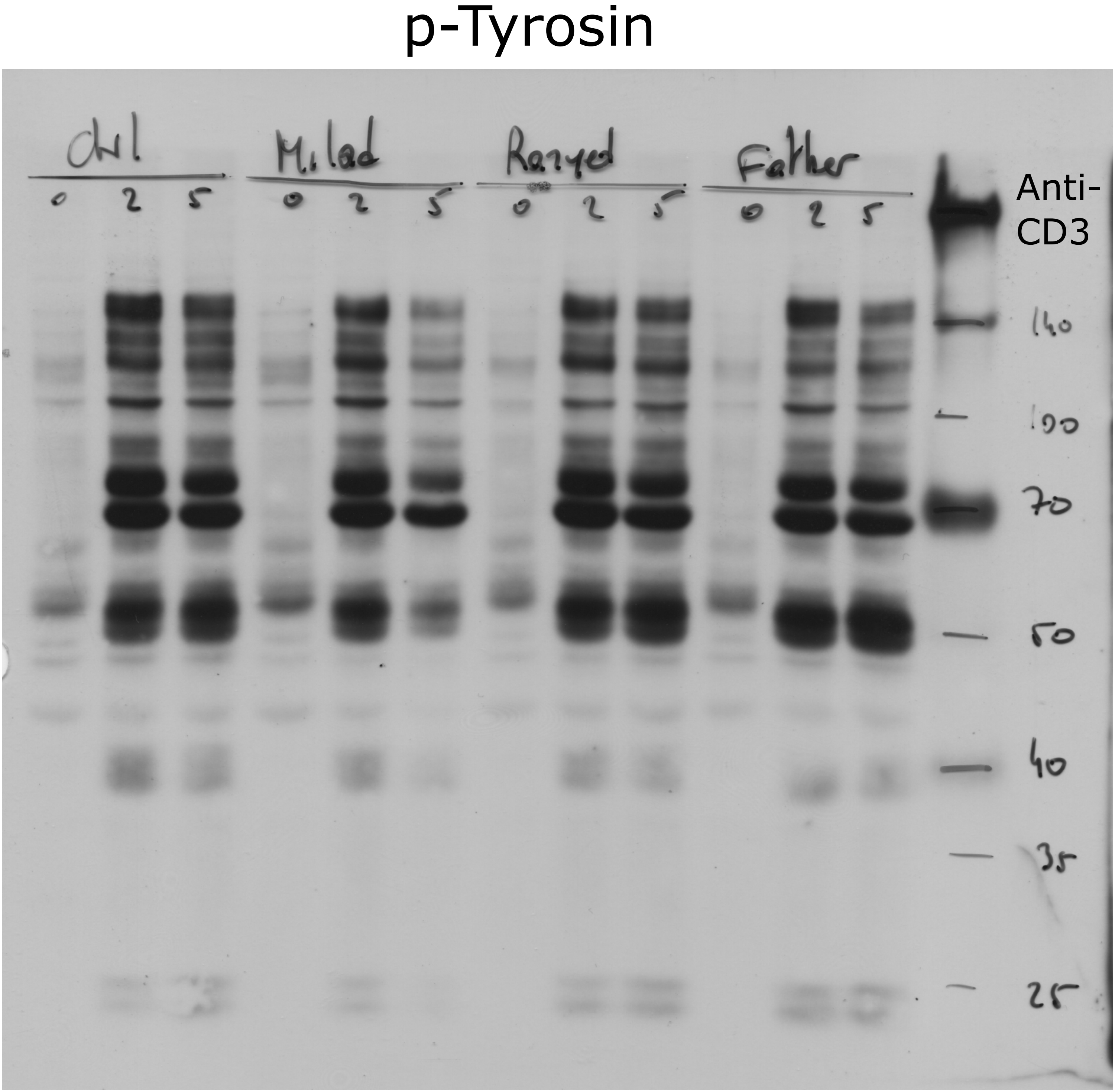
p-Tyrosin
Ctrl
Milad
Father
0 2 5
0 2 5
0 2 5
0 2 5
Anti-CD3
140
100
70
50
40
35
25

p-PLCg1

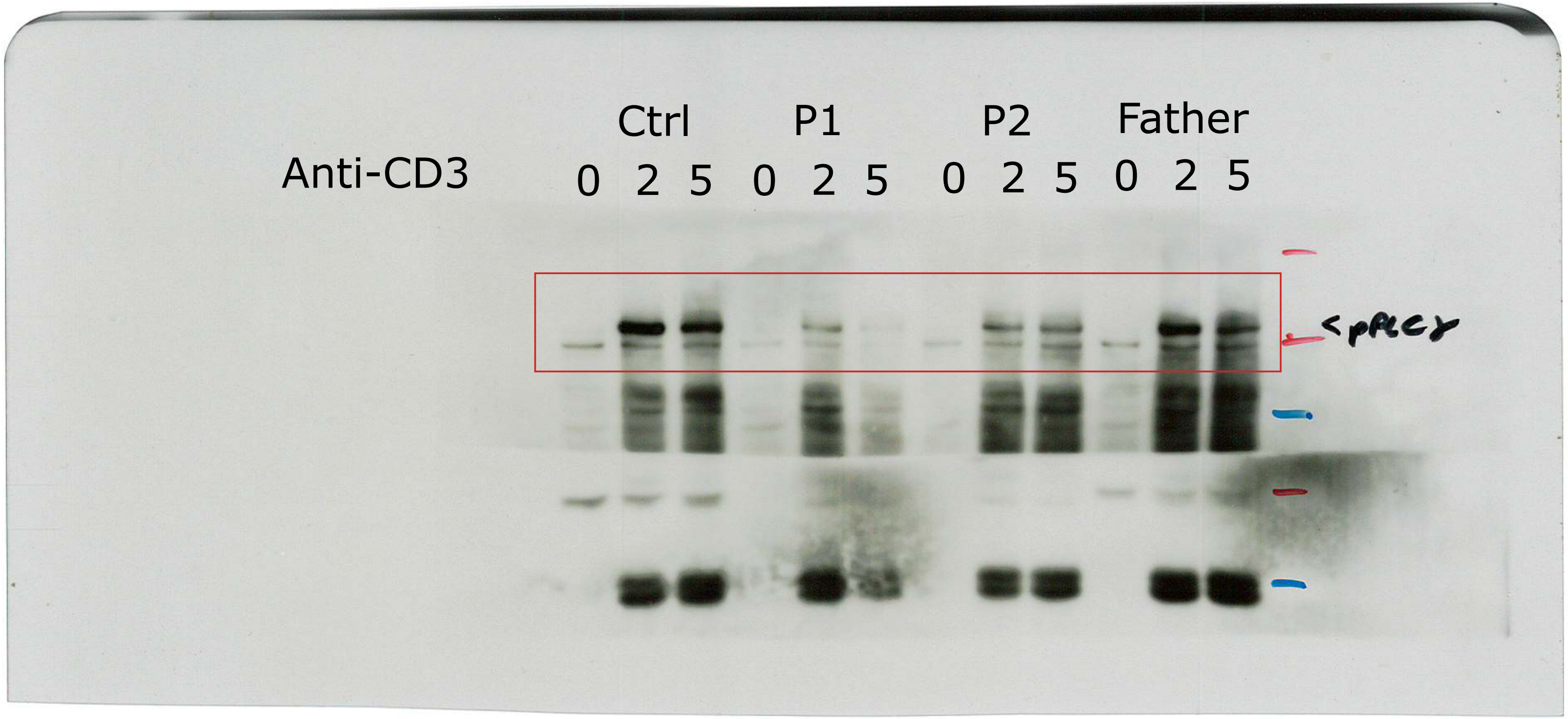

PLCg1

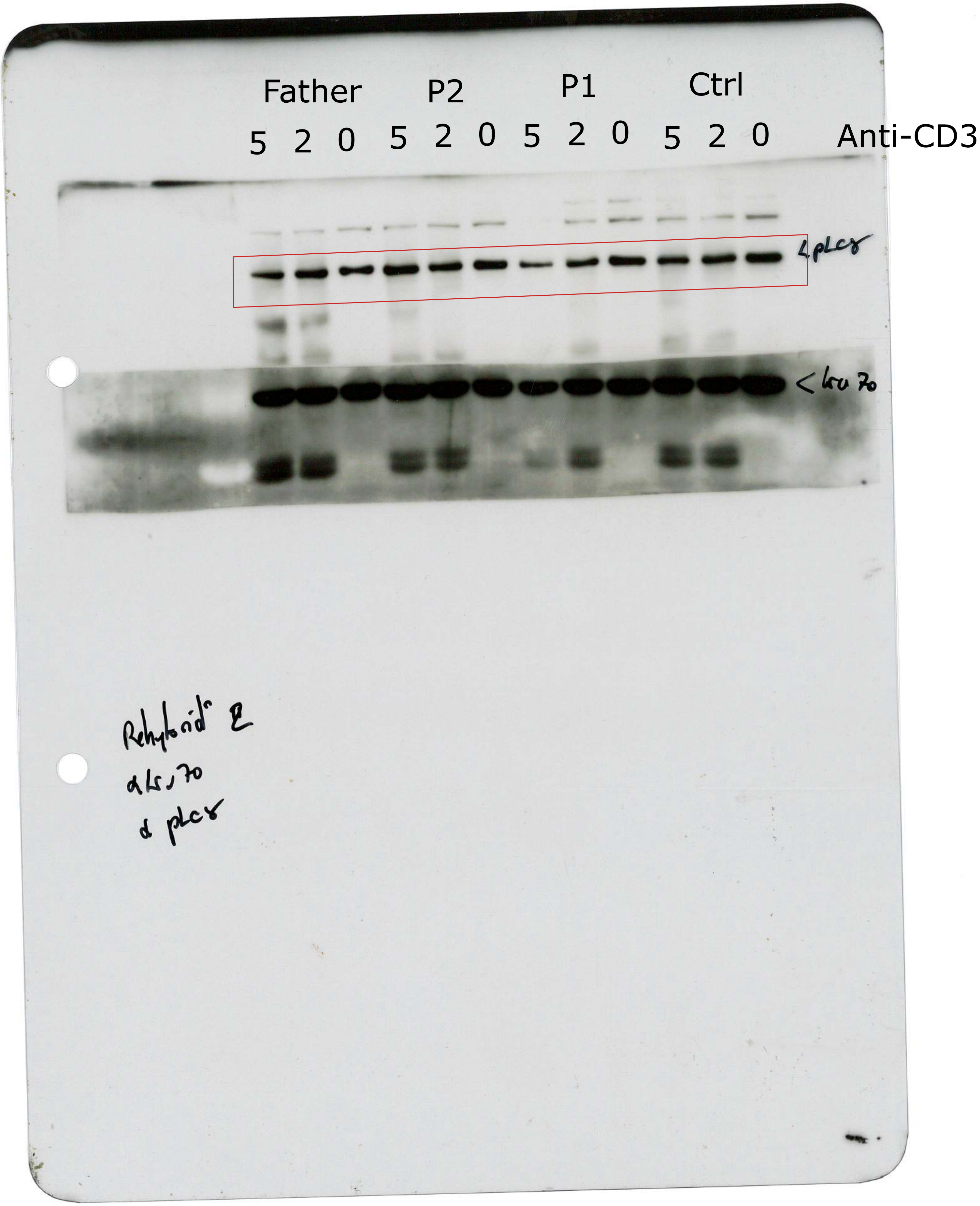

KU70

The written annotation is actually inverted.
The correct annotation is shown in red.

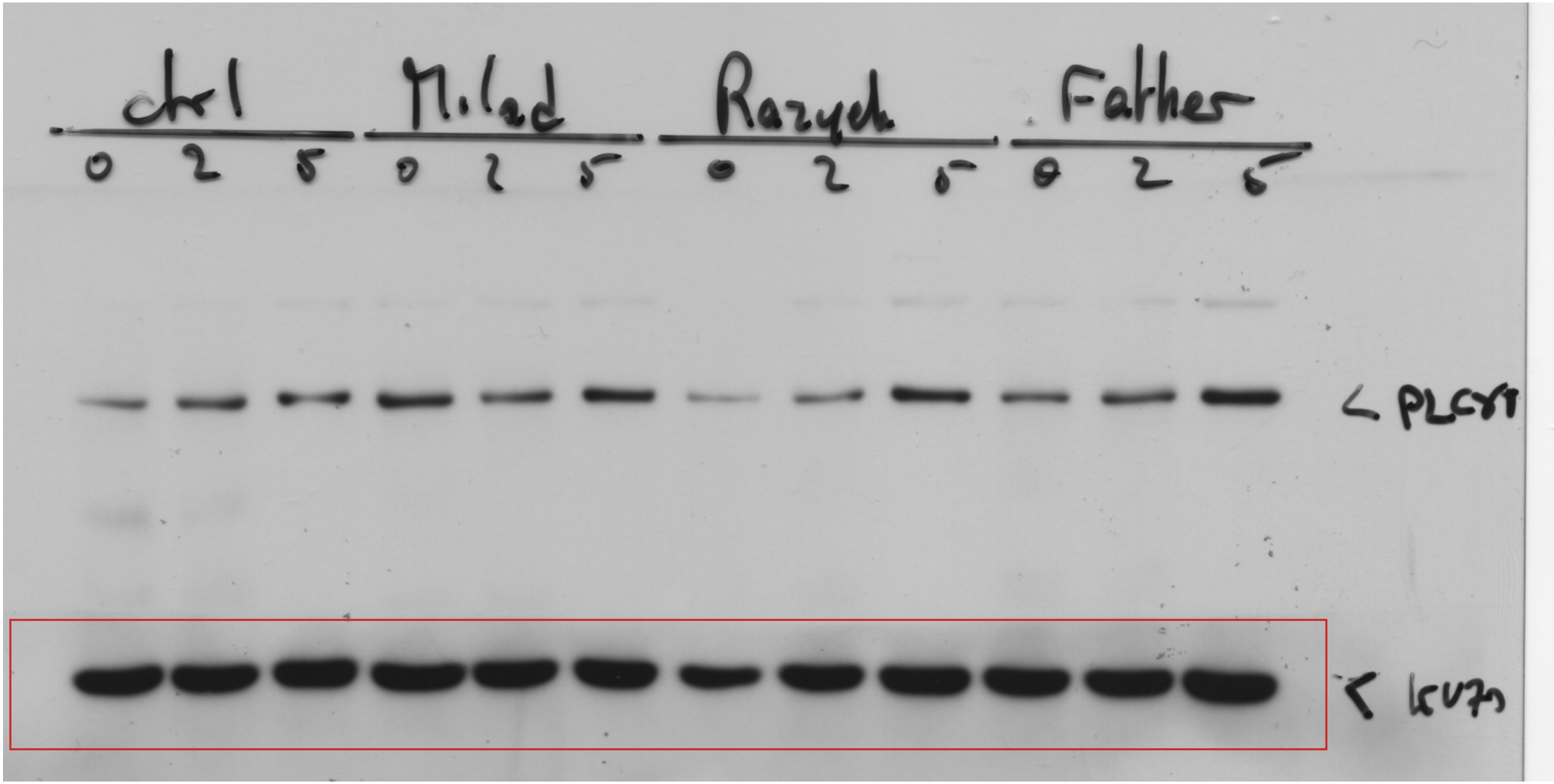

5 2 0 5 2 0 5 2 0 5 2 0 Anti-CD3

Father P2 P1 Ctrl
