# Supplementary figures and images for: Inherited human ITK deficiency impairs IFN-γ immunity and underlies tuberculosis
Source: J Exp Med. 2022 Nov 3;220(1):e20220484. doi: 10.1084/jem.20220484 (PMC9641312; doi:10.1084/jem.20220484)

# p-ZAP70

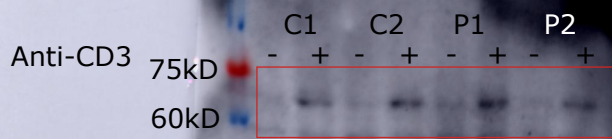

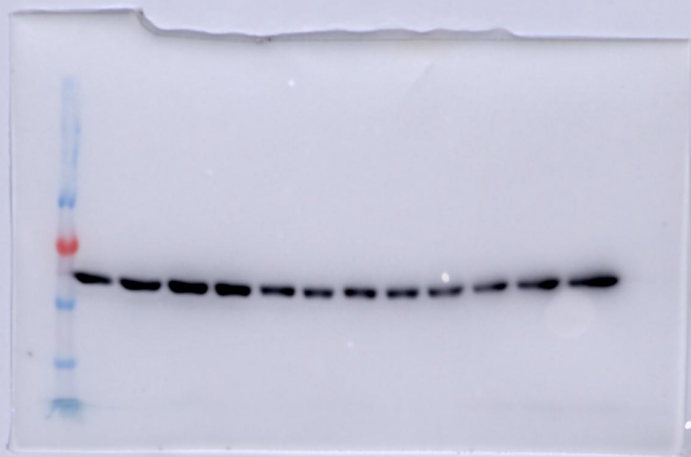

# ZAP70

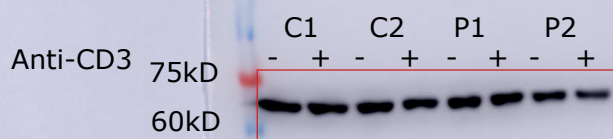

Supplement: SourceData FS3 — contains original blots for Fig. S3. [file JEM_20220484_SourceDataFS3.pdf]
